# Carfilzomib Induces Cardiotoxicity by Blocking Autophagic Flux Through the cGAS-STING Signaling Pathway

**DOI:** 10.3390/biom16060854

**Published:** 2026-06-11

**Authors:** Shizhong Liu, Xianghong Hou, Daiqianhui Li, Zhenli Guo, Xin Zhou, Yan Wang, Ketao Ma, Rui Yang, Xinzhi Li

**Affiliations:** 1 The Key Laboratory of Xinjiang Endemic and Ethnic Diseases, Ministry of Education, Shihezi University Medical College, Shihezi 832002, China; 13345463523@163.com (S.L.); 15299949265@163.com (X.H.); ld129714@163.com (D.L.); zl_guo2025@163.com (Z.G.); zx19730804@163.com (X.Z.); wangyan_9011@shzu.edu.cn (Y.W.); maketao@shzu.edu.cn (K.M.); 2 Department of Physiology, Shihezi University Medical College, Shihezi 832002, China; 3 Department of Pathophysiology, Shihezi University Medical College, Shihezi 832002, China; 4 State Key Laboratory of Vascular Homeostasis and Remodeling, Peking University Health Science Center, Beijing 100191, China

**Keywords:** carfilzomib, autophagy, cGAS-STING, SNARE proteins

## Abstract

Carfilzomib (CFZ) is a proteasome inhibitor primarily used to treat relapsed and refractory multiple myeloma. However, its clinical application is limited by significant cardiotoxicity, the underlying mechanisms of which remain incompletely understood. In this study, we aimed to elucidate the pathogenic pathways involved. In vitro, CFZ induced mitochondrial dysfunction and apoptosis in AC16 cardiomyocytes in a concentration- and time-dependent manner. Transcriptomic analysis revealed enrichment in pathways related to autophagy and endoplasmic reticulum stress. Mechanistically, CFZ promoted autophagosome formation but downregulated the SNARE proteins STX17, SNAP29, and VAMP8, thereby impairing autophagosome–lysosome fusion and blocking autophagic flux. This disruption was associated with the activation of the cGAS-STING signaling pathway. In vivo, CFZ administration resulted in cardiac dysfunction and apoptosis in mice, both of which were attenuated by the STING inhibitor C-176. Consistently, *STING* knockdown restored autophagic flux and reduced cardiomyocyte injury in vitro. In conclusion, CFZ induces cardiotoxicity by activating the cGAS-STING pathway, which disrupts the autophagic clearance of damaged mitochondria and promotes cardiomyocyte apoptosis. Targeting *STING* may represent a promising therapeutic strategy to mitigate CFZ-induced cardiotoxicity.

## 1. Introduction

Carfilzomib is a novel epoxide proteasome inhibitor that can irreversibly bind to structural and immune proteasomes, thereby preventing the growth and development of tumor cells [1]. Clinically, carfilzomib is mainly used to treat patients with relapsed and refractory multiple myeloma (RRMM) and has shown significant therapeutic effects [2]. Clinically, the incidence of cardiovascular adverse events (CVAEs) in patients treated with carfilzomib has been reported to be as high as 18–22%, which is notably higher than that observed with bortezomib (approximately 5–8%) [3]. At present, the main clinical approaches to addressing carfilzomib cardiotoxicity involve reducing the dose of carfilzomib or discontinuing the drug. There is a lack of effective anticarfilzomib cardiotoxicity treatment methods.

Autophagy is a process through which cells utilize lysosomes to break down damaged or excess cellular components, playing a crucial role in maintaining cellular homeostasis, responding to stress, and eliminating pathogens [4,5]. In the autophagy process, the fusion barrier between autophagosomes and lysosomes is the most common cause of autophagic flux obstruction. In recent years, the role of SNARE complexes in autophagy and related diseases has received increasing attention, gradually becoming an important research target in regulating autophagy. Previous studies have shown that in mammalian cells, this process is driven by two sets of SNARE protein complexes, STX17-SNAP29-VAMP8 and STX7-SNAP29-YKT6 [6]. The composition of SNARE complexes mainly includes three different types of proteins: vesicle-associated membrane protein (VAMP), synaptic fusion protein (STX), and synaptic vesicle-associated protein (SNAP), which together are referred to as SNARE proteins. In the autophagy process, SNARE proteins that play key roles include VAMP8, STX17, and SNAP29 [7]. VAMP8 is an important member of the SNARE protein family and is mainly responsible for membrane fusion activities within the cell, playing a crucial role in the binding of autophagosomes and lysosomes [8]. SNAP29, owing to its lack of transmembrane domains, is guided to the autophagosome only after STX17 is transferred to the autophagosome and then interacts with STX17 to be directed to the autophagosome [9] and further interacts with VAMP8 on the lysosomal membrane to promote the fusion of autophagosomes and lysosomes, thereby regulating the autophagy process. To date, no study has investigated the relationship between carfilzomib-induced cardiotoxicity and SNARE complexes; thus, we investigated how CFZ affects SNARE complexes.

In recent years, numerous experiments have shown that the cGAS-STING signaling pathway plays a significant role in the cardiovascular system. Hu et al. reported that inhibiting the expression of cGAS after acute myocardial infarction promotes the transformation of macrophages to the M2 phenotype, thereby alleviating myocardial injury after myocardial infarction and promoting myocardial repair [10]. Additionally, Hu et al. reported that in a mouse model of heart failure induced by pressure overload, inhibiting the cGAS-STING signaling pathway could reduce inflammatory factor levels and inflammatory cell infiltration and inhibit cardiomyocyte apoptosis, thereby improving myocardial remodeling and protecting cardiac function [11]. Moreover, STING can control the autophagy and energy metabolism induced by energy stress through STX17 and inhibit the assembly of the STX17-SNAP29-VAMP8 complex [8,9]. Finally, RNA sequencing analysis showed that it was closely related to endoplasmic reticulum stress, protein processing in the endoplasmic reticulum, and autophagy-related pathways. Therefore, we investigated the relationships among carfilzomib, the cGAS-STING signaling pathway, and SNARE proteins.

## 2. Materials and Methods

### 2.1. Materials

Carfilzomib (CFZ; S51335) was obtained from Shanghai Yuanye Biotechnology Co. (Shanghai, China). The STING inhibitor C-176 (C-176; S88645) was obtained from Shanghai Yuanye Biotechnology Co. (Shanghai, China). Primary antibodies against Bax (ab32503), Bcl-2 (ab182858) and caspase-3 (ab184787) were obtained from Abcam (Cambridge, UK). LC3 (14600-1-AP), P62/SQSTM1 (18420-1-AP), STX17 (17815-1-AP), SNAP29 (12704-1-AP), VAMP8 (15546-1-AP), cGAS (26416-1-AP), TMEM173/STING (19851-1-AP), and β-actin (66009-1-Ig) antibodies were obtained from Proteintech Biotechnology Co. (Wuhan, China). ATG5 (T55766), Beclin-1 (T55092), and ULK1 (T56902) were obtained from Abmart (Shanghai, China). The secondary antibodies, goat anti-rabbit IgG (ZB-2306) and goat anti-mouse IgG (ZB-2305), were acquired from Zhong Shan-Golden Bridge Biological Technology Co. (Beijing, China). Kits for lactate dehydrogenase (LDH; A020-2-2) were obtained from Nanjing Jiancheng Biotechnology Co., Ltd. (Nanjing, China). Creatine kinase isoenzyme (CK-MB; JL12422), brain natriuretic peptide (BNP; JL12884), and cardiac troponin T (cTnT; JL40538) kits were purchased from Shanghai Jianglai Industry Co., Ltd. (Shanghai, China). Cell viability was assessed via Cell Counting Kit 8 (CCK-8) detection kits from APExBIO Technology LLC (Shanghai, China). The Annexin V-FITC/PI Apoptosis Kit was procured from Multisciences Biotech Co. (Hangzhou, China). Dimethyl sulfoxide (DMSO), RIPA buffer, and phenylmethylsulfonyl fluoride (PMSF) were purchased from Solarbio Science & Technology Co., Ltd. (Beijing, China).

### 2.2. In Vivo Experiments

#### 2.2.1. Animals and Experimental Protocols

Male C57 mice weighing 20–25 g were obtained from Hunan Slake Jingda Laboratory Animal Co., Ltd. (Wuhan, China). The mice were housed at the Animal Experimental Breeding Center of Shihezi University, where they were provided with suitable living conditions. All animal experiments and procedures were approved by the Biology Ethics Committee of Shihezi University. The Approval Number is A2025-624, and the specific date of ethics approval is 17 February 2025. A total of four intraperitoneal injections of CFZ (8 mg/kg each) were administered, with each injection spaced 48 h apart. Using Km factors (mouse Km = 3, human Km = 37), the HED for our mouse dose was calculated as HED = 8 mg/kg × (3/37) ≈ 0.65 mg/kg. The animal experiments were divided into two batches:

1. First batch

The control group (control) was treated with 8 mg/kg solvent (the solvent was PEG300 + TWEEN80 + DMSO + normal saline).

The CFZ group (CFZ) received 8 mg/kg CFZ solution (the solvent was PEG300 + TWEEN80 + DMSO + normal saline).

2. Second batch

The control group (control) received 8 mg/kg of solvent (the solvent was PEG300 + TWEEN80 + DMSO + normal saline) + the same amount of corn oil.

In the CFZ group (CFZ), 8 mg/kg CFZ solution (the solvent was PEG300 + TWEEN80 + DMSO + normal saline) + the same amount of corn oil was administered.

The CFZ + C-176 group (CFZ + C-176) received 8 mg/kg CFZ solution (the solvent was PEG300 + TWEEN80 + DMSO + normal saline) + 750 nmol of C-176 (the solvent was corn oil).

Within 24 h after the last intraperitoneal injection, the mice were anesthetized. Blood was subsequently collected from the orbital cavity, and the chest cavity was quickly exposed. The heart was then removed. One part was treated with 4% paraformaldehyde, and the other part was treated with liquid nitrogen, which was used for HE, Masson, TUNEL, and WB assays.

#### 2.2.2. Echocardiographic Examination

Echocardiographic examinations were conducted at two time points: on day 0 and 24 h after the last administration (on the 8th day). The mice were anesthetized and fixed on the VINNO6VET (VINNO Technology Co., Ltd., Suzhou, China) small animal-specific detection platform preset in a warm bed. The fur on the chest was removed, and a thin layer of coupling agent was applied. A left ventricular short-axis M-mode echocardiogram was obtained using the MX550D (VINNO Technology Co., Ltd., Suzhou, China) probe (frequency 30 MHz) at the 3rd and 4th intercostal spaces on the left sternal border through the chest wall. A distance of 10 mm was set to ensure a clear display of the interventricular septum and the left ventricular posterior wall within the left ventricular short axis. One left ventricular short-axis image was selected from each of the three stable cardiac cycles for the segmentation of the interventricular septum and the left ventricular posterior wall. The left ventricular ejection fraction (EF%) and short-axis shortening rate (FS%) were analyzed via VevoLAB (v5.9.0) analysis software. The average value of three measurements was taken for each mouse.

#### 2.2.3. Cardiac Injury-Associated Enzymes

To assess cardiac function and myocardial injury in mice, we measured the activities of BNP (brain natriuretic peptide), cTnT (cardiac troponin T), and CK-MB (creatine kinase isoenzyme) in blood samples using an ELISA kit, which were acquired from Jianglai Biotechnology Co., Ltd. (Shanghai, China). The samples were prepared according to the kit instructions. The samples were added to a 96-well plate. After the reaction was completed, the absorbance was measured using an enzyme-linked immunosorbent assay (ELISA) instrument to evaluate the enzymatic activities of BNP, cTnT, and CK-MB.

#### 2.2.4. Histopathological Study

To evaluate the degree of pathological change and degree of fibrosis in the mouse heart tissue, the heart tissue was fixed with 4% paraformaldehyde, embedded in paraffin, and cut into 5-micron-thick sections. These sections were stained with H&E and Masson’s trichrome. The stained sections were analyzed and photographed under an optical microscope.

#### 2.2.5. Terminal Deoxynucleotidyl Transferase dUTP Nick End Labeling (TUNEL)

To determine the percentage of apoptotic cells, TUNEL staining was performed on cardiac tissue samples to detect apoptotic cells. The procedure followed the manufacturer’s instructions. TUNEL were acquired from Servicebio Biotechnology Co., Ltd. (Wuhan, China). The degree of cardiomyocyte death was quantified by calculating the ratio of TUNEL-positive nuclei to the total number of cardiac cells in the field of view.

### 2.3. In Vitro Experiments

#### 2.3.1. Cell Culture and Treatments

AC16 cells were purchased from Servicebio Biotechnology Co., Ltd. (Wuhan, China), located in Wuhan (STCC13101P). These cells were cultured in DMEM supplemented with 10% fetal bovine serum and 1% penicillin–streptomycin. The cells were divided into the control group and the CFZ group, with concentration gradients (0.01, 0.1, 1, and 10 μM) and time gradients (12 h and 24 h). In the experiment, if not specified otherwise, the CFZ group received a 1 μM intervention for 24 h.

#### 2.3.2. Cell Viability Assay

The cells were added to culture media supplemented with different concentrations (0, 0.01, 0.1, 1 and 10 μM) of the CFZ solution for 12 h, 18 h, 24 h, 30 h, or 36 h. After the culture was completed, the culture medium was replaced with new medium containing the CCK-8 reagent (APExBio). After an additional 2–3 h of further cultivation, the absorbance at 450 nm was measured using a microplate reader from Thermo Fisher Scientific (Waltham, MA, USA). By comparing the measured values with the absorbance values of the untreated control cells, we determined the cell viability.

#### 2.3.3. Lactate Dehydrogenase Assay

The supernatant from the cell culture was collected, and the release of lactate dehydrogenase (LDH) was assessed according to the kit instructions to determine whether the cells were damaged. The absorbance was measured at 450 nanometers.

#### 2.3.4. Flow Cytometry Assay and Annexin-V/PI Assay for Apoptosis

An apoptosis assay kit (Annexin V-FITC/PI) was used to evaluate the apoptosis status of the cells. We added 5 microliters of Annexin V-FITC and 10 microliters of PI solution to the cells, which were then analyzed via flow cytometry. The percentage of apoptotic cells was calculated via FlowJo V10 software.

#### 2.3.5. Immunofluorescence Experiment

CFZ was diluted to concentrations of 0, 0.01, 0.1, 1, and 10 μM, and the original medium was replaced with medium containing CFZ. One milliliter was added to each well, and the cells were further cultured in the incubator for 24 h. The cells were subsequently fixed, permeabilized, blocked, incubated with antibodies, and mounted. Subsequently, fluorescence microscopy was used for observation, and images were captured.

#### 2.3.6. JC-1 Staining Combined with Flow Cytometry

The cell treatment was the same as that described in Section 2.3.5. Using the JC-1 kit, AC16 cells were treated according to the instructions, and fluorescence microscopy observations and flow cytometry analysis were carried out.

#### 2.3.7. RNA Knockdown

The small interfering RNA (siRNA) targeting *STING* was provided by Gemaco Gene Pharmaceuticals (Suzhou, China). A nonspecific siRNA was used as a negative control. The cells were transfected with the transfection reagent provided by Gemaco Gene Pharmaceuticals and cultured according to the manufacturer’s protocol for 48 h. The transfection efficiency was evaluated via Western blotting. The specific sequence of *STING* is 5′-CUGGCAUGGUCAUAUUACATT-3′, and its complementary sequence is 3′-UGUAAUAUGACCAUGCCAGTT-5′.

#### 2.3.8. Electron Microscopy

The AC16 cells were evenly distributed into five 60 mm dishes. When the cell density reached approximately 60%, 0, 0.01, 0.1, 1, or 10 μM carfilzomib was added, and the cells were incubated in an incubator for 24 h. After the intervention was complete, the samples were washed twice with PBS, 1 mL of PBS was added to each dish, and then a cell scraper was used to collect the AC16 cells, which were subsequently centrifuged. The samples were washed twice with precooled PBS and centrifuged; the supernatants were discarded, and the cells were placed in a 2.5% glutaraldehyde solution for transmission electron microscopy (Hitachi, Ltd., Tokyo, Japan) fixation overnight at 4 °C. Afterwards, the samples were processed according to the requirements of transmission electron microscopy and observed.

#### 2.3.9. mCherry-GFP-LC3 Adenovirus Transfection for Detecting Autophagic Flux

The cells were digested and centrifuged according to the cell passage procedure to obtain the cell precipitate. After the AC16 cells were counted, they were inoculated into a twelve-well plate at a density of 1 × 10^5^ cells/well (24 h), and 0.5 mL of volume was added to each well. The plate was then placed in a 37 °C incubator with 5% CO_2_ for cultivation. 100 MOI viruses were added to each well, and the twelve-well plate was returned to the incubator for further cultivation. The twelve-well plate was removed from the incubator, the plate was rinsed three times with PBS, and a fluorescence quenching agent containing DAPI was added to each well. Next, forceps were used to lift the cover glass, the cells were placed downward on the slide, and the slide was sealed with nail polish. Finally, the samples were stored in a 4 °C environment under light protection. Images were obtained via confocal fluorescence microscopy.

#### 2.3.10. Quantitative Real-Time Polymerase Chain Reaction (qRT–PCR)

Total RNA was extracted from cells via E.Z.N.A (Norcross, GA, USA). A whole-cell RNA kit from Omega (Omega Bio-tek, Norcross, GA, USA) was used, and the concentration of RNA was measured with a NanoDrop spectrophotometer (Thermo Fisher Scientific, USA). One microgram of RNA was used to synthesize cDNA with the First-Strand DNA Synthesis Kit with Reverse Transcriptase I (Thermo Fisher Scientific) according to the manufacturer’s instructions. Amplification was performed using the QuantiNova SYBR Green PCR Kit (Käger, Dietzenbach, Germany) and the LightCycler 96 Real-Time Fluorescence Quantitative PCR Instrument (Roche, Rotkreuz, Switzerland) according to the manufacturer’s instructions. Relative expression levels were calculated via the 2−ΔΔCt method. *β*-actin was used as the control. The primers were synthesized by Shanghai Sanggene Biotechnology Company (Shanghai, China), and their sequences are as follows:

*cGAS*-FP: 5′-CGGGAGCTACTATGAGCACG-3′

*cGAS*-RP: 5′-AAGTGTTACAGCAGGGCTCC-3′

*STING*-FP: 5′-TACAACAACCTGCTACGGGG-3′

*STING*-RP: 5′-TCATCTGCAGGTTCCGCTG-3′

*β-ACTIN*-FP: 5′-AACCGCGAGAAGATGACCCAG-3′

*β-ACTIN*-RP: 5′-GGATAGCACAGCCTGGATAGCA-3′

### 2.4. Western Blot Analysis

RIPA buffer, protease inhibitor, and phosphatase inhibitor were mixed at a ratio of 100:1:1 to prepare the lysis buffer, which was used to extract proteins from cardiac tissue or cells, and the protein concentration was determined using a BCA assay kit. The proteins were separated via 10% SDS–PAGE and transferred onto a PVDF membrane. The membrane was blocked at room temperature with 5% nonfat milk for 2 h and then incubated with the primary antibody at 4 °C overnight. Afterwards, the membrane was incubated with a horseradish peroxidase-labeled secondary antibody (diluted 1:20,000) for 2 h. The chemiluminescence signal was detected using an enhanced chemiluminescence (ECL) kit, and images were captured via a chemiluminescence imaging system. Original figures can be found in Supplementary Materials.

### 2.5. RNA Extraction and RNA Sequencing Analysis

The AC16 cells were divided into a control group and a CFZ group. RNA was extracted from AC16 cells using TRIzol (Thermo Fisher Scientific, Waltham, MA, USA) reagent, and the samples were then sent to NovelBio (Shanghai, China) for further analysis. The transcriptome data were processed via the OmicShare platform (https://www.omicshare.com/, accessed on 1 April 2025). The dynamic GSEA (https://www.gsea-msigdb.org/gsea/index.jsp, accessed on 10 April 2025) enrichment tool was utilized, and “human (GRCh38.p13)” was selected as the species. The Molecular Feature Database (MSigDB) was also employed to identify characteristic gene sets. By analyzing the expression patterns of differentially expressed genes (DEGs), it was possible to determine the key targets and mechanism of action of carfilzomib-induced myocardial injury.

### 2.6. KEGG and GO Analysis

We applied the DESeq2 algorithm to filter the differentially expressed genes. After the significant analysis, *p*-value and FDR analysis were subjected to the following criteria: (i) Fold Change >2 or <0.5; (ii) *p*-value < 0.05, FDR < 0.05. KEGG and GO enrichment analyses were performed on the transcriptome data via the OmicShare platform (https://www.omicshare.com/, accessed on 28 March 2025).

### 2.7. Statistical Analysis

Statistical analysis was performed using the GraphPad Prism 8.0 software. When quantitative data follow a normal distribution, they are described by mean ± standard deviation. The one-way analysis of variance (ANOVA) method is used for comparison among multiple groups. If the ANOVA results show significant differences and satisfy homogeneity of variance, the Least Significant Difference (LSD) method is used for multiple comparisons between groups. If the results of the analysis of variance are significantly different and do not satisfy homogeneity of variance, Dunn’s multiple comparisons should be used. When the quantitative data do not follow a normal distribution, the median and interquartile range are used for description, and non-parametric tests are used for comparison among multiple groups. Statistical significance was defined as *p* < 0.05.

## 3. Results

### 3.1. Carfilzomib Can Cause Myocardial Damage and Promote the Occurrence of Fibrosis

#### 3.1.1. Intraperitoneal Injection of Carfilzomib Decreased the Survival Rate of Mice and Impaired Their Cardiac Function

**Figure 1:**
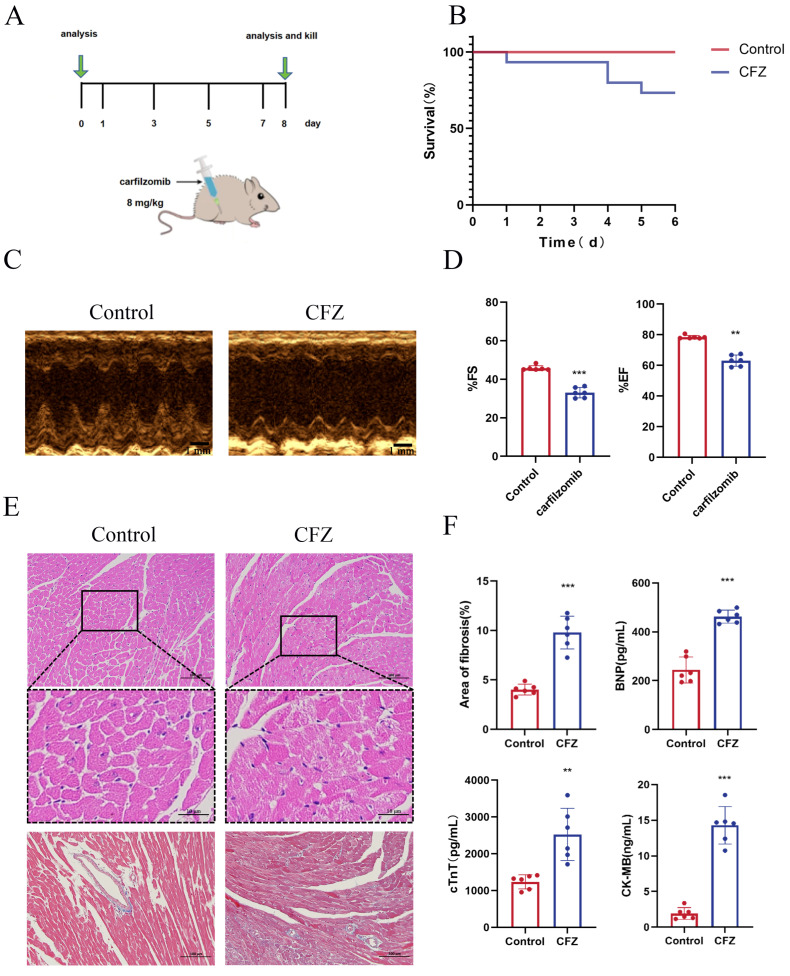
Carfilzomib can induce cardiac dysfunction in C57BL/6 mice. (**A**) Model establishment method for C57BL/6J mice. (**B**) Survival curves of C57BL/6J mice after carfilzomib intervention. (*n* = 15) (**C**) Echocardiogram of C57BL/6J mice (scale bar = 1 mm). (**D**) Statistical graph of the shortening fraction and ejection fraction. (**E**) H&E staining and Masson staining (scale bar = 100 μm or 10 μm). (**F**) Statistical graph of Masson staining and serum contents of BNP, cTNT, and CK-MB. Data represent the mean ± SD, *n* = 6; ** *p* < 0.01, *** *p* < 0.001 vs. the control group.

As shown in Figure 1A, we administered 8 mg/kg carfilzomib via intraperitoneal injection to C57BL/6 mice once every other day. This resulted in survival curves for the mice in the control group and the carfilzomib group, as shown in Figure 1B and Supplementary Figure S1C. The survival rate in the control group of mice was 100%, while four mice in the CFZ group died, with a survival rate of 66.67%. After the last injection of carfilzomib in the treatment group, echocardiography was performed on the mice, as shown in Figure 1C. Compared with those in the control group, the left ventricular internal diameter at end-diastole (LVIDd) and the left ventricular internal dimension at end-systole (LVIDs) in the CFZ group were significantly reduced, indicating a decrease in myocardial contractility. The cardiac contraction and ejection functions of the control group and the CFZ group were subsequently statistically analyzed, as were the shortening fraction (FS%) and ejection fraction (EF%). Compared with those of the control group, the FS% and EF% of the CFZ group significantly decreased (Figure 1D), and the differences were statistically significant (*p* < 0.01 or *p* < 0.001). These results indicate that carfilzomib has significant myocardial toxicity and can cause a decrease in myocardial contraction and ejection.

#### 3.1.2. Pathological Morphological Changes in the Myocardial Tissues of Each Group of Mice

HE staining was used to observe the effect of carfilzomib on the pathological tissue structure of the heart in mice. The results revealed that in the myocardial tissue of the control group, the morphology of the myocardial cells was normal, with clear longitudinal and transverse stripes, and that the muscle fibers were arranged in an orderly manner. In the CFZ group, the cardiomyocytes of the mice presented varying degrees of edema, vacuolated degeneration, disordered arrangement of cardiomyocytes, loss of cytoplasm, nuclear consolidation, inflammatory cell infiltration, and even hemorrhage (Figure 1E).

#### 3.1.3. Changes in the Levels of cTnT, CK-MB, and BNP in the Serum of Each Group of Mice

We used ELISA to detect changes in serum cTnT, CK-MB, and BNP levels in the CFZ-induced mouse heart model. As shown in Figure 1F, the serum CK-MB and BNP levels in the CFZ group of mice significantly increased, indicating that the heart was damaged after the administration of carfilzomib (*p* < 0.001); at the same time, the cTnT level significantly increased (*p* < 0.01).

### 3.2. Carfilzomib Can Induce Apoptosis in Mouse Cardiac Muscle Cells

**Figure 2:**
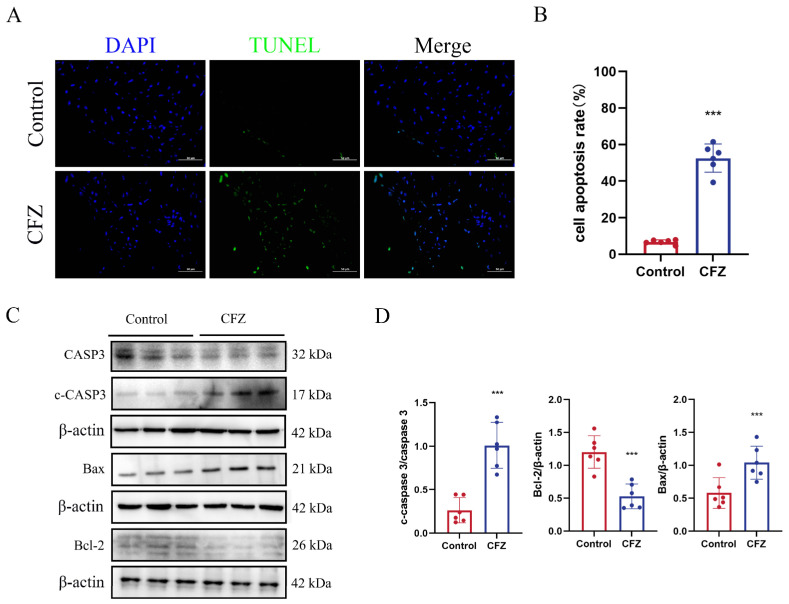
Carfilzomib can induce apoptosis in the cardiac tissue of C57BL/6J mice. (**A**,**B**) Fluorescence images and statistical graphs of TUNEL staining of heart tissue from C57BL/6J mice (scale bar = 50 μm). (**C**,**D**) Western blot results and statistical graphs of the heart tissue from C57BL/6J mice. Data represent the mean ± SD, *n* = 6; *** *p* < 0.001 vs. the control group.

The effect of carfilzomib on the apoptosis of mouse cardiomyocytes was evaluated via TUNEL staining. As shown in Figure 2A,B, compared with that in the control group, the amount of green fluorescence in the cardiomyocytes of the CFZ group was significantly greater, indicating that the number of apoptotic cardiomyocytes was significantly higher. These findings suggest that CFZ can induce the apoptosis of mouse cardiomyocytes, and the difference was statistically significant (*p* < 0.001). To further verify the effect of carfilzomib on apoptosis in mouse myocardial tissue, we used protein immunoblotting to detect the expression of apoptosis-related proteins in mouse myocardial tissue. As shown in Figure 2C,D, compared with that in the control group, the expression of the proapoptotic protein Bax in the CFZ group increased, while the expression of the antiapoptotic protein Bcl-2 decreased; at the same time, the expression of cleaved caspase-3 in the CFZ group was significantly greater than that in the control group, and the difference was statistically significant (*p* < 0.001).

### 3.3. Carfilzomib Intervention Can Decrease the Viability of AC16 Cells and Induce Apoptosis

#### 3.3.1. Establishment of a Cell Damage Model in AC16 Cells Induced with Carfilzomib

**Figure 3:**
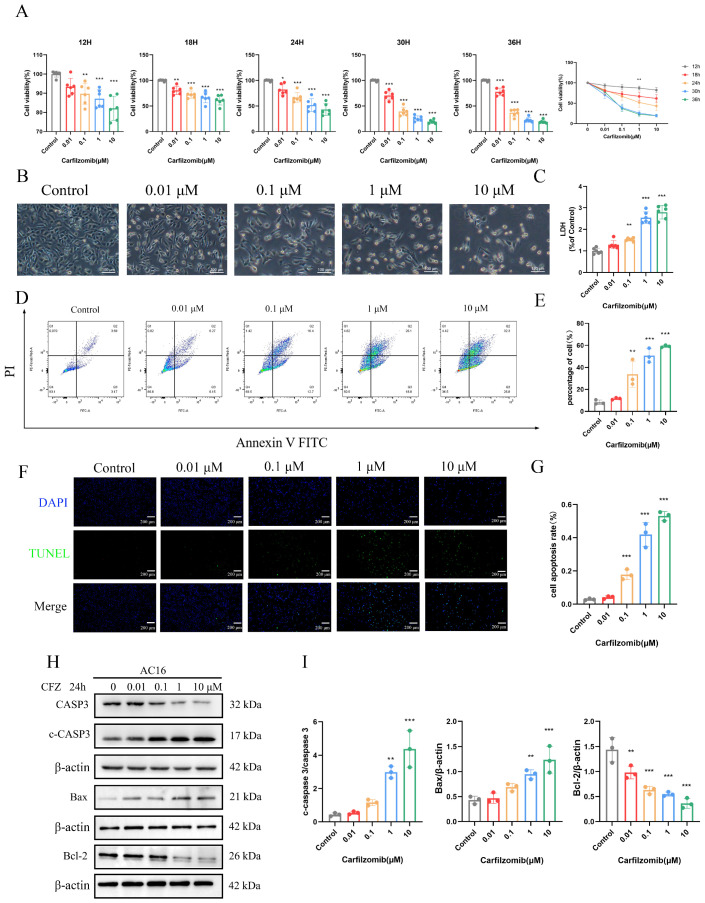
Carfilzomib can damage AC16 cardiac muscle cells and promote apoptosis. (**A**) CCK-8 was used to measure the viability of AC16 cardiomyocytes after CFZ treatment. (**B**) Morphological changes in AC16 cardiomyocytes under an optical microscope (scale bar = 100 μm). (**C**) LDH content in the supernatant of AC16 cardiomyocytes. (**D**,**E**) Flow cytometry was used to detect changes in the apoptosis of AC16 cardiomyocytes after CFZ intervention. (**F**,**G**) TUNEL staining was used to detect changes in the apoptosis rate of AC16 cardiomyocytes after CFZ intervention (scale bar = 200 μm). (**H**,**I**) Western blot results and statistical graphs of apoptosis-related proteins in AC16 cardiomyocytes after CFZ intervention. Data represent the mean ± SD, *n* = 3, 6; * *p* < 0.05, ** *p* < 0.01, *** *p* < 0.001 vs. the control group.

To verify the cardiotoxicity of carfilzomib in vitro, we first used the CCK-8 method to detect the survival rate of AC16 cells. As shown in Figure 3A, we applied different concentrations of carfilzomib (0, 0.01, 0.1, 1, or 10 μM) for 12 h, 18 h, 24 h, 30 h, or 36 h of intervention. The statistical results revealed that as the concentration of carfilzomib increased, the survival rate of AC16 cells decreased, indicating that carfilzomib inhibits the viability of AC16 cells in a concentration-dependent manner; at the same time, as the intervention time increased, the survival rate of AC16 cells also decreased, indicating that carfilzomib inhibits cell viability in a time-dependent manner. Because the cell survival rate was close to 50% when 1 μM carfilzomib was applied for 24 h and there were significant differences between different time points at the 1 μM concentration, in the subsequent experiments, we chose the 1 μM carfilzomib intervention for 24 h as the default intervention condition.

The effect of carfilzomib on the morphology of AC16 cells was observed under an optical microscope. As shown in Figure 3B, under normal conditions, AC16 cells were adherent and fibroblast-like, and only a few cells died in the field of view. As the concentration of carfilzomib increased, the density of AC16 cells gradually decreased, the cell volume decreased, and the number of dead AC16 cells increased. The morphology of the dead AC16 cells became transparent and fragment-like. Changes in LDH content were detected using an LDH kit to evaluate the effect of carfilzomib on damage to AC16 cells. The results are shown in Figure 3C. After carfilzomib intervention, the LDH level in AC16 cells significantly increased, and the LDH level in the cells also increased with increasing CFZ concentration (*p* < 0.01 or *p* < 0.001), indicating that carfilzomib can cause damage to AC16 cells in a concentration-dependent manner.

#### 3.3.2. Carfilzomib Can Induce Apoptosis in AC16 Cells

The apoptosis rate of AC16 cells after carfilzomib intervention was detected via the Annexin V-FITC/PI double-staining method combined with flow cytometry. The results shown in Figure 3 indicate that, compared with the control group, the number of apoptotic cells in the Q2 + Q3 area of the CFZ group significantly increased, and the proportion of apoptotic cells gradually increased with increasing drug concentration, suggesting that carfilzomib can induce the apoptosis of AC16 cells in a concentration-dependent manner. To visually observe the effect of carfilzomib on cardiomyocyte apoptosis, we used TUNEL staining to detect changes in the apoptosis level of AC16 cells induced by CFZ. As shown in Figure 3F,G, compared with that of the control group, the fluorescence intensity of the CFZ group increased, indicating that the level of apoptosis in AC16 cells increased after carfilzomib administration and that the degree of apoptosis increased with increasing concentration, and the difference was statistically significant (*p* < 0.001).

Apoptotic-related proteins were detected via protein immunoblotting to investigate the effect of carfilzomib on the apoptosis of AC16 cells in vitro. The results are shown in Figure 3H,I. Compared with those in the control cells, the expression levels of the proapoptotic proteins Bax and cleaved caspase-3 in the CFZ-treated cells significantly increased, and the protein expression levels gradually increased with increasing concentration. The expression levels of the antiapoptotic protein Bcl-2 decreased, and the protein expression levels gradually decreased with increasing concentration. The differences were statistically significant (*p* < 0.01 or *p* < 0.001). These findings indicate that carfilzomib can induce the apoptosis of AC16 cells in a concentration-dependent manner.

### 3.4. Carfilzomib Intervention Can Lead to Mitochondrial Damage in AC16 Cells

**Figure 4:**
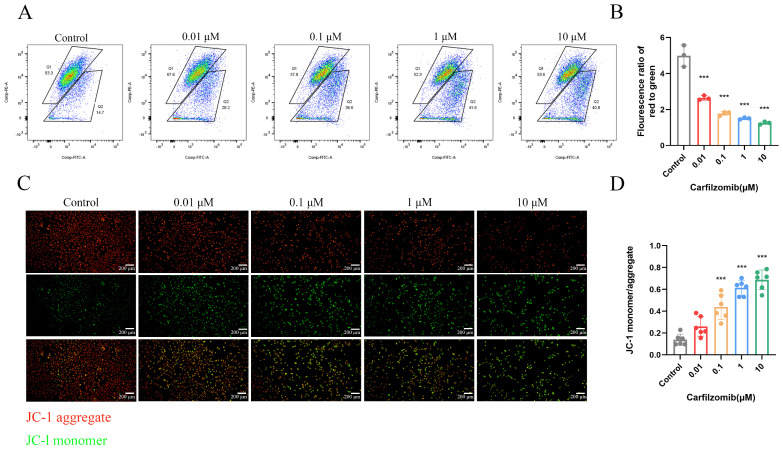
Carfilzomib can damage the mitochondria of AC16 cardiac muscle cells, resulting in a decrease in the mitochondrial membrane potential. (**A**,**B**) Flow cytometry analysis of JC-1 staining results and statistical analysis. (**C**,**D**) JC-1 fluorescence results and statistical data (scale bar = 200 μm). Red fluorescence indicates JC-1 aggregates, whereas green fluorescence indicates JC-1 monomers. Data represent the mean ± SD, *n* = 3, 6; *** *p* < 0.001 vs. the control group.

The effect of carfilzomib on the mitochondrial membrane potential of AC16 cardiomyocytes was detected via JC-1 combined with flow cytometry. The results are shown in Figure 4A,B. The Q1 area represents red fluorescence, and the Q2 area represents green fluorescence. Compared with the control, CFZ significantly lowered the mitochondrial membrane potential of AC16 cells, and the change in the mitochondrial membrane potential became more significant as the concentration of CFZ increased. These results suggest that CFZ can damage mitochondria, alter the mitochondrial membrane potential, and exert a concentration-dependent effect (*p* < 0.001).

The JC-1 fluorescence method was used to more intuitively observe changes in the mitochondrial membrane potential of AC16 cells after carfilzomib intervention. The JC-1 fluorescence results are shown in Figure 4C,D. These results suggest that carfilzomib can induce mitochondrial damage and lower the mitochondrial membrane potential in AC16 cells in a concentration-dependent manner.

### 3.5. Transcriptomics Combined with Experiments to Explore the Mechanism Behind Carfilzomib-Induced Damage in AC16 Cells

#### 3.5.1. RNA Sequencing Assay for Detecting Differentially Expressed Genes in AC16 Cells Induced with Carfilzomib

**Figure 5:**
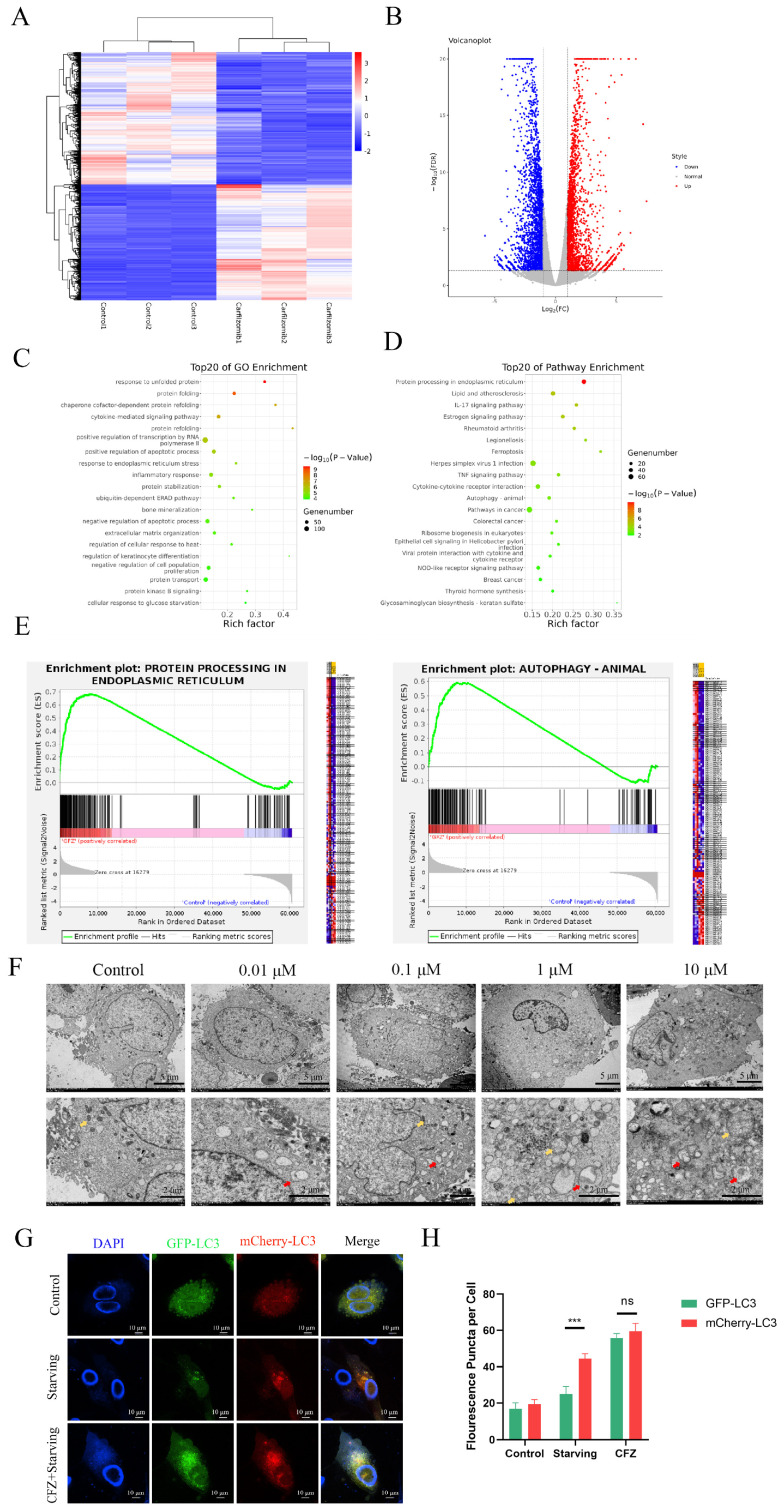
Through transcriptomics combined with experiments, it was found that the cardiotoxicity induced by carfilzomib is closely related to protein processing in the endoplasmic reticulum, mitochondrial damage, and autophagy. (**A**,**B**) Graph showing the mRNA transcriptome results of AC16 cardiomyocytes after carfilzomib intervention. (**C**,**D**) Graphs showing the results of the KEGG and GO enrichment analyses. (**E**) Graph showing the enrichment analysis results of GSEA. The clear version of the blurred image has be contained in Supplementary Materials. (**F**) Graph showing the transmission electron microscopy results of AC16 cardiomyocytes after carfilzomib intervention (scale bar = 5 μm or 2 μm). The yellow arrows indicate mitochondria, and the red arrows indicate autophagosomes. (**G**,**H**) Graphs and statistical charts showing the changes in autophagic flux after mCherry-GFP-LC3 adenovirus transfection of AC16 cells (scale bar = 10 μm). Data represent the mean ± SD, *n* = 3; *** *p* < 0.001 vs. the control group, ns indicates no statistically significant difference.

The AC16 cells were divided into a control group and a CFZ group. Transcriptome sequencing was performed on both groups of cells (Figure S2A–D). The results revealed that, by screening genes with an absolute value of log2FC greater than 1 and a *p* value less than 0.05, we identified a total of 4626 genes, including 2155 upregulated genes and 2470 downregulated genes. Figure 5A,B show the heatmap and volcano plot of the genes that were differentially expressed after carfilzomib intervention. Blue represents downregulated genes, and red represents upregulated genes.

#### 3.5.2. Functional and Pathway Enrichment Analyses of Differentially Expressed Genes

GO and KEGG enrichment analyses of the differentially expressed genes were performed. As shown in Figure 5C, the GO enrichment analysis results indicated that the DEGs were closely related to biological processes such as the response to unfolded proteins, protein folding, protein refolding, and endoplasmic reticulum stress; as shown in Figure 5D, the KEGG enrichment analysis results indicated that the DEGs were closely related to pathways such as protein processing in the endoplasmic reticulum and autophagy. Differential gene set enrichment analysis (GSEA) was subsequently conducted for protein processing in the endoplasmic reticulum and autophagy-related pathways, and the results are shown in Figure 5E. Thus, it can be concluded that carfilzomib intervention mainly activates endoplasmic reticulum stress and autophagy in AC16 cells.

#### 3.5.3. Transmission Electron Microscopy Was Used to Observe Changes in the Mitochondria and Autophagosomes of AC16 Cells in Each Group

In Figure 5F, the yellow arrows point to mitochondria, and the red arrows point to autophagosomes. In normal AC16 cells, the mitochondrial morphology is normal, and there is no abnormal increase in the number of autophagosomes. After intervention with carfilzomib, the mitochondria in AC16 cells become enlarged, the mitochondrial structure is damaged, and the mitochondrial cristae disappear. The number of autophagosomes increases. Moreover, as the concentration of carfilzomib increases, mitochondrial damage becomes more severe, and the number of autophagosomes gradually increases. The results of transmission electron microscopy suggest that carfilzomib can damage mitochondria and activate the formation of autophagosomes in a concentration-dependent manner.

#### 3.5.4. Using mCherry-GFP-LC3 to Detect the Effect of Carfilzomib on the Autophagic Flux of AC16 Cells

To further verify the effect of carfilzomib on the formation of autophagic flux in AC16 cells, mCherry-GFP-LC3 adenovirus was transfected into AC16 cells, and the cells were divided into the control group, the starvation group, and the starvation + carfilzomib group. After the intervention, the fluorescence intensity of each group was observed via laser confocal fluorescence microscopy. As shown in Figure 5G,H, compared with that in the control group, the proportion of red fluorescence increased after starvation treatment; after the administration of carfilzomib, both green and red fluorescence increased, but there was no difference in the ratio of green fluorescence to red fluorescence. These results suggest that starvation treatment can activate autophagy, and, after the administration of carfilzomib, autophagic flux is blocked.

### 3.6. Autophagosome Formation Was Activated After Carfilzomib Intervention

#### 3.6.1. In Vivo Experiments Verified That Carfilzomib Activates the Formation of Autophagosomes

**Figure 6:**
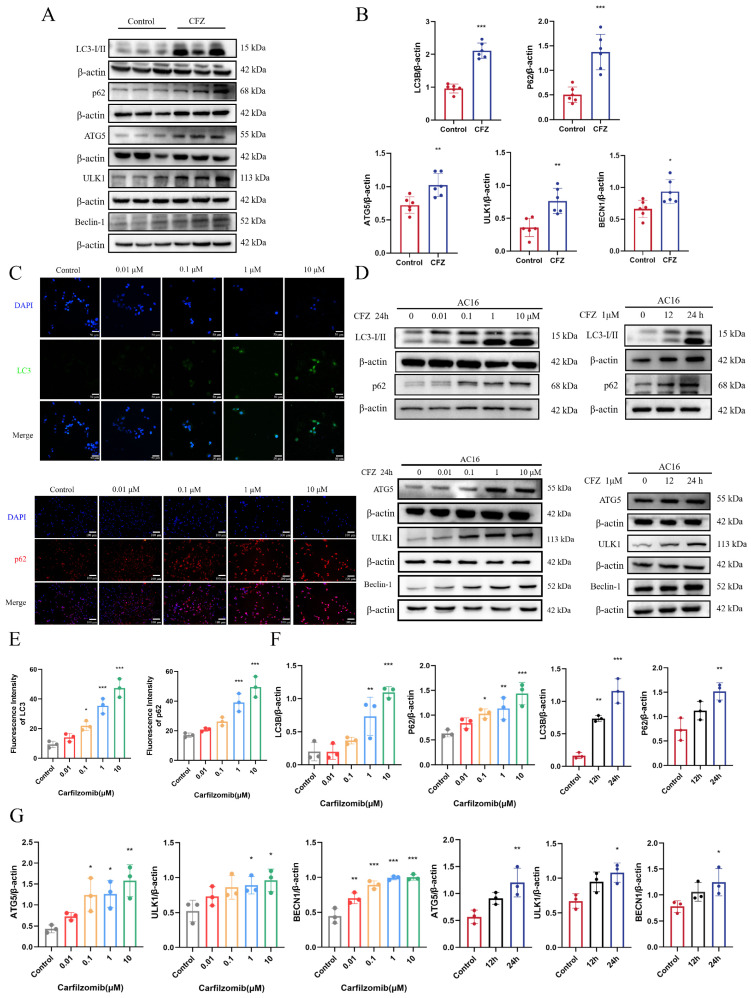
Carfilzomib can induce autophagy in cardiac tissue cells from C57BL/6J mice and in AC16 cardiomyocytes. (**A**,**B**) Western blot results and statistical graphs of the levels of autophagy-related proteins in mouse heart tissue. (**C**) Immunofluorescence results of the LC3 and p62 proteins in AC16 cardiomyocytes after carfilzomib intervention (scale bar = 50 or 100 μm). (**D**) Expression of autophagy-related proteins in AC16 cardiomyocytes after carfilzomib intervention. (**E**) Statistical analysis of the immunofluorescence results. (**F**,**G**) Statistical graph of the Western blot results. Data represent the mean ± SD, *n* = 3, 6; * *p* < 0.05, ** *p* < 0.01, *** *p* < 0.001 vs. the control group.

We used protein immunoblotting to detect the expression of autophagy-related proteins, as shown in Figure 6A,B. Compared with that in the control group, the expression of LC3 in the CFZ group increased, indicating that the formation of autophagosomes in mice after the administration of carfilzomib increased; however, after the administration of carfilzomib, the expression of p62 in the mouse heart tissue also increased, and this change might be due to the blockade of autophagic flux and the reduced degradation of p62, with a statistically significant difference (*p* < 0.001). These results suggest that the mechanism of cardiac toxicity induced by carfilzomib may be related to autophagy. To verify the ability of carfilzomib to activate autophagy in mouse myocardial tissue, the expression of autophagy initiation-related proteins was detected via protein immunoblotting. Compared with the control group, the expression of ATG5, ULK1, and Beclin-1 in the CFZ group increased, indicating that the formation of autophagosomes in mice increased after carfilzomib administration, and the difference was statistically significant (*p* < 0.05 or *p* < 0.01).

#### 3.6.2. In Vitro Experiments Verified That Carfilzomib Activates the Formation of Autophagosomes

As shown in Figure 6D,F,G, compared with those in the control group, the expression levels of LC3 and p62 increased after the administration of CFZ, and the expression levels gradually increased with increasing concentration; as the duration of CFZ intervention increased, the expression levels of LC3 and p62 also increased. These results suggest that the increase in LC3 may be due to the activation of autophagosomes and that the increase in the level of p62 may be due to the blockage of autophagic flux, resulting in the accumulation of p62. The results at the animal level are consistent with these findings, suggesting that carfilzomib-induced cardiomyocyte damage is closely related to autophagy. To further verify the relationship between the cytotoxicity of carfilzomib and autophagy in AC16 cells, we used Western blotting to detect the expression of autophagy activation-related proteins. Compared with those in the control group, the expression levels of ATG5, ULK1, and Beclin-1 increased after carfilzomib administration, and the expression levels gradually increased with increasing carfilzomib concentration; as the intervention time with carfilzomib increased, the expression levels of ATG5, ULK1, and Beclin-1 also increased (*p* < 0.05, *p* < 0.01, or *p* < 0.001). These results suggest that after intervention with carfilzomib, autophagosomes formed in AC16 cells in a time- and concentration-dependent manner. Moreover, immunofluorescence staining was used to further observe the expression and localization of the LC3 and p62 proteins in AC16 cells. As shown in Figure 6C,E, blue fluorescence represents DAPI staining of the cell nucleus, and green fluorescence represents the LC3 protein, which is expressed in both the nucleus and the cytoplasm. Compared with that in the control group, the green fluorescence in the carfilzomib-treated group increased, and the fluorescence intensity gradually increased with increasing carfilzomib concentration. These findings indicate that after the administration of carfilzomib, the protein expression level of LC3 increased in a concentration-dependent manner. The red fluorescence represents the p62 protein, which is expressed in both the nucleus and the cytoplasm; compared with that in the control group, the red fluorescence in the carfilzomib-treated group increased in a concentration-dependent manner. These results are consistent with the Western blot results.

### 3.7. Carfilzomib Blocked the Formation of Autophagic Lysosomes

#### 3.7.1. In Vivo Experiments Verified That Carfilzomib Inhibits the Formation of Autophagic Lysosomes

**Figure 7:**
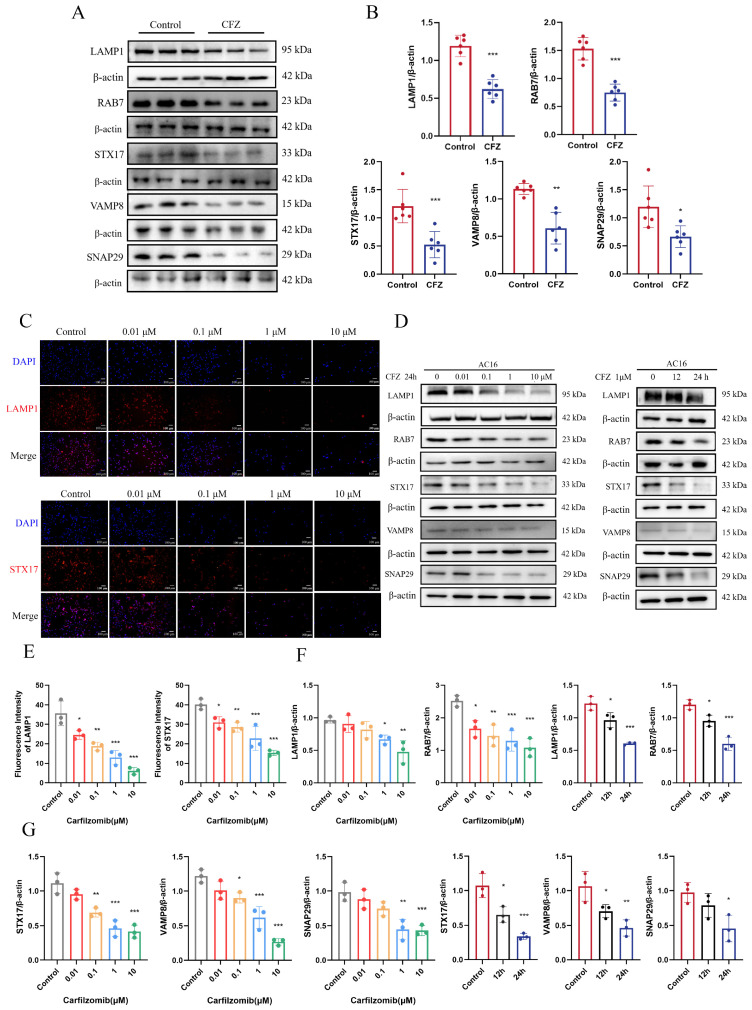
Carfilzomib can inhibit the formation of autophagic lysosomes in the cardiac tissue cells of C57BL/6J mice and the cardiomyocytes of AC16. (**A**,**B**) Western blot results and statistical graphs of autophagosome-related proteins in mouse heart tissue. (**C**) Immunofluorescence results of LAMP1 and STX17 proteins in AC16 cardiomyocytes after carfilzomib intervention (scale bar = 100 μm). (**D**) Expression of autophagosome-related proteins in AC16 cardiomyocytes after carfilzomib intervention. (**E**) Statistical analysis of the immunofluorescence results. (**F**,**G**) Statistical graphs of the Western blot results. Data represent the mean ± SD, *n* = 3, 6; * *p* < 0.05, ** *p* < 0.01, *** *p* < 0.001 vs. the control group.

To verify the effect of carfilzomib on autophagic lysosomes in mouse myocardial tissue, changes in the expression of autophagic lysosome-related proteins in this tissue were detected via protein immunoblotting. As shown in Figure 7A,B, LAMP1 and RAB7 proteins were expressed in mouse myocardial tissue. Compared with those in the control group, the protein expression levels of LAMP1 and RAB7 in the mouse heart tissue of the CFZ group were significantly lower (*p* < 0.001), indicating that the formation of autophagic lysosomes decreased and that their function weakened after the administration of CFZ. These results suggest that carfilzomib inhibits the formation of autophagic lysosomes in mouse myocardial tissue. The main SNARE complex that dominates autophagic lysosome fusion is composed of STX17 and SNAP29 on the autophagosome membrane and VAMP8 on the late lysosome. The STX17, VAMP8, and SNAP29 proteins are expressed in mouse myocardial tissue. Compared with those in the control group, the protein expression levels of STX17, VAMP8, and SNAP29 in the mouse heart tissue of the CFZ group were significantly lower (*p* < 0.05, *p* < 0.01 or *p* < 0.001), indicating that the formation of autophagy-related SNARE complexes decreased after the administration of carfilzomib. These results show that carfilzomib inhibits the formation of lysosomes in mouse myocardial tissue and is related to the inhibition of SNARE complex formation.

#### 3.7.2. In Vitro Experiments Verified That Carfilzomib Inhibits the Formation of Autophagic Lysosomes

To further verify the mechanism through which carfilzomib affects autophagic flux, Western blotting was used to detect the effects of carfilzomib on the expression of autophagic lysosome-related proteins in AC16 cells. As shown in Figure 7D–G, compared with that in the control group, the protein expression level of LAMP1 in AC16 cells significantly decreased after treatment with carfilzomib, indicating that autophagic lysosome function was weakened after the administration of carfilzomib; the protein level of RAB7 decreased after the administration of carfilzomib, indicating that the formation of autophagic lysosomes was reduced. Compared with those in the control group, the protein expression levels of STX17, VAMP8, and SNAP29 in AC16 cells significantly decreased after carfilzomib administration, indicating that the formation and function of autophagy-related SNARE complexes were reduced after carfilzomib administration. These results suggest that carfilzomib inhibited the formation of autophagy-related SNARE complexes in AC16 cells (*p* < 0.05, *p* < 0.01, or *p* < 0.001). Otherwise, our data suggest transcriptional downregulation for VAMP8/SNAP29 and a post-transcriptional mechanism for STX17 (Figure S1A).

We used immunofluorescence to detect the effect of carfilzomib on the protein expression of LAMP1 in AC16 cells. As shown in Figure 7C,E, compared with that in the control group, the red fluorescence in the carfilzomib-treated group decreased, and the fluorescence intensity of red fluorescence decreased as the concentration increased. These findings indicate that after the administration of carfilzomib, the protein expression of LAMP1 decreased in a concentration-dependent manner. Additionally, compared with that in the control group, the red fluorescence of STX17 decreased after the administration of carfilzomib, and the fluorescence intensity of red fluorescence became weaker as the concentration increased. These findings indicate that after the administration of carfilzomib, the protein expression of STX17 decreased in a concentration-dependent manner (*p* < 0.05, *p* < 0.01, or *p* < 0.001).

### 3.8. Carfilzomib Inhibits the Expression of SNARE Proteins by Activating the cGAS-STING Signaling Pathway, Thereby Blocking Autophagic Flux

**Figure 8:**
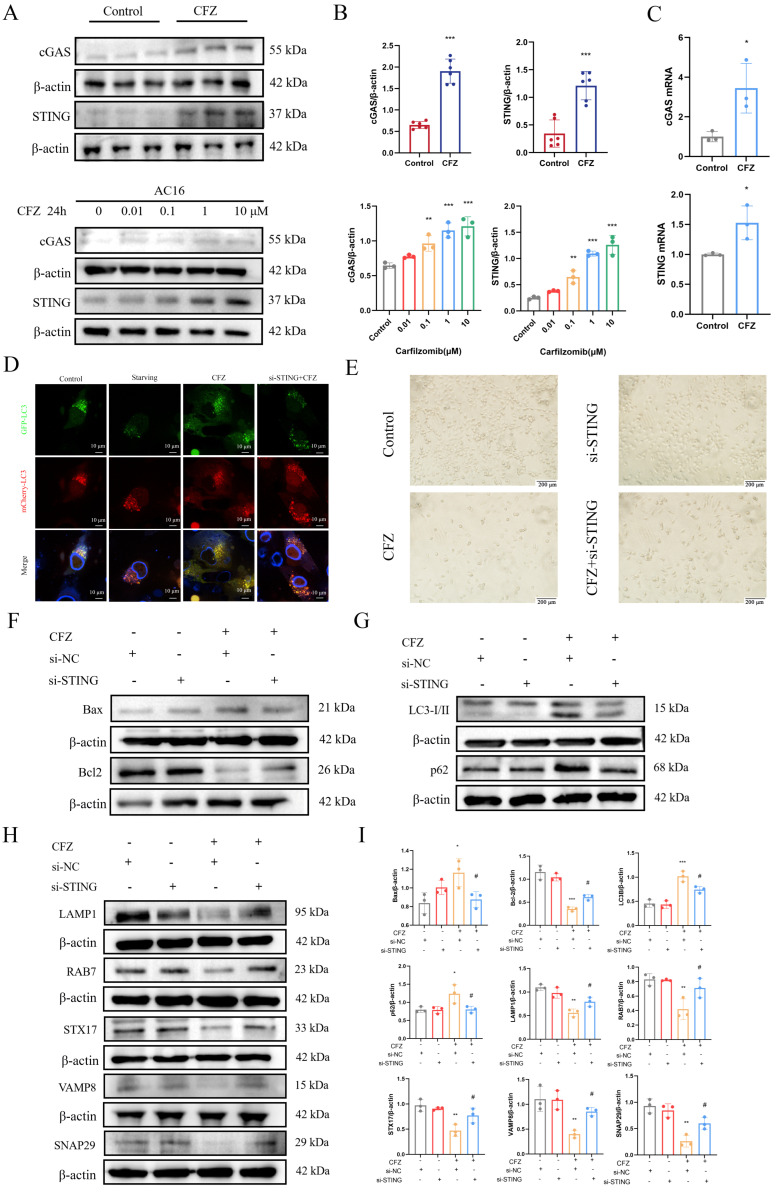
Carfilzomib can activate the cGAS-STING signaling pathway. Knocking down STING can reverse the cardiac toxicity caused by carfilzomib. (**A**,**B**) Western blot results and statistical graphs of cGAS and STING protein expression. (**C**) Statistical graph of changes in cGAS and STING mRNA expression. (**D**) Changes in autophagic flux after *STING* knockdown were detected via mCherry-GFP-LC3 adenovirus transfection (scale bar = 10 μm). (**E**) Changes in the cell state after *STING* knockdown were observed under an optical microscope (scale bar = 200 μm). (**F**–**I**) Western blot results and statistical graphs of related proteins in AC16 cardiomyocytes after STING knockdown. Data represent the mean ± SD, *n* = 3, 6; * *p* < 0.05, ** *p* < 0.01, *** *p* < 0.001 vs. the control group; # *p* < 0.05 vs. the CFZ group.

Western blot experiments combined with qRT–PCR verified that carfilzomib activated the cGAS-STING signaling pathway, as shown in Figure 8A–C. We verified through in vivo and in vitro experiments that the protein expression levels of cGAS and STING increased after carfilzomib administration and that the expression of cGAS and STING gradually increased with increasing carfilzomib concentration, indicating a concentration-dependent effect. Additionally, through qRT–PCR experiments, it was found that the expression of mRNAs also increased, and the statistical results were statistically significant (*p* < 0.05, *p* < 0.01 or *p* < 0.001).

### 3.9. Knocking Down STING Restored Autophagic Flux in AC16 Cells and Reversed the Cardiotoxicity of Carfilzomib

As shown in Figure 8D, after the knockdown of *STING* (Figure S1B), the green fluorescence was weaker than that in the CFZ group, indicating that the knockdown of *STING* can restore the autophagic flux blocked by carfilzomib. Under a light microscope, the cell state after the knockdown of *STING* was significantly better than that in the CFZ group (Figure 8E). Through Western blot experiments to verify the changes in protein expression, after the knockdown of *STING*, the apoptosis of AC16 cells decreased, the autophagic flux was restored, and the expression of autophagic lysosome proteins and SNARE complex proteins increased (Figure 8F–I) (*p* < 0.05, *p* < 0.01, or *p* < 0.001).

### 3.10. In Vivo Experiments Demonstrated That the Cardiotoxicity of Carfilzomib Was Reversed After the Use of the STING Inhibitor C-176 and That Apoptosis in Mouse Heart Tissues Was Reduced, Whereas Autophagic Flux Was Restored

**Figure 9:**
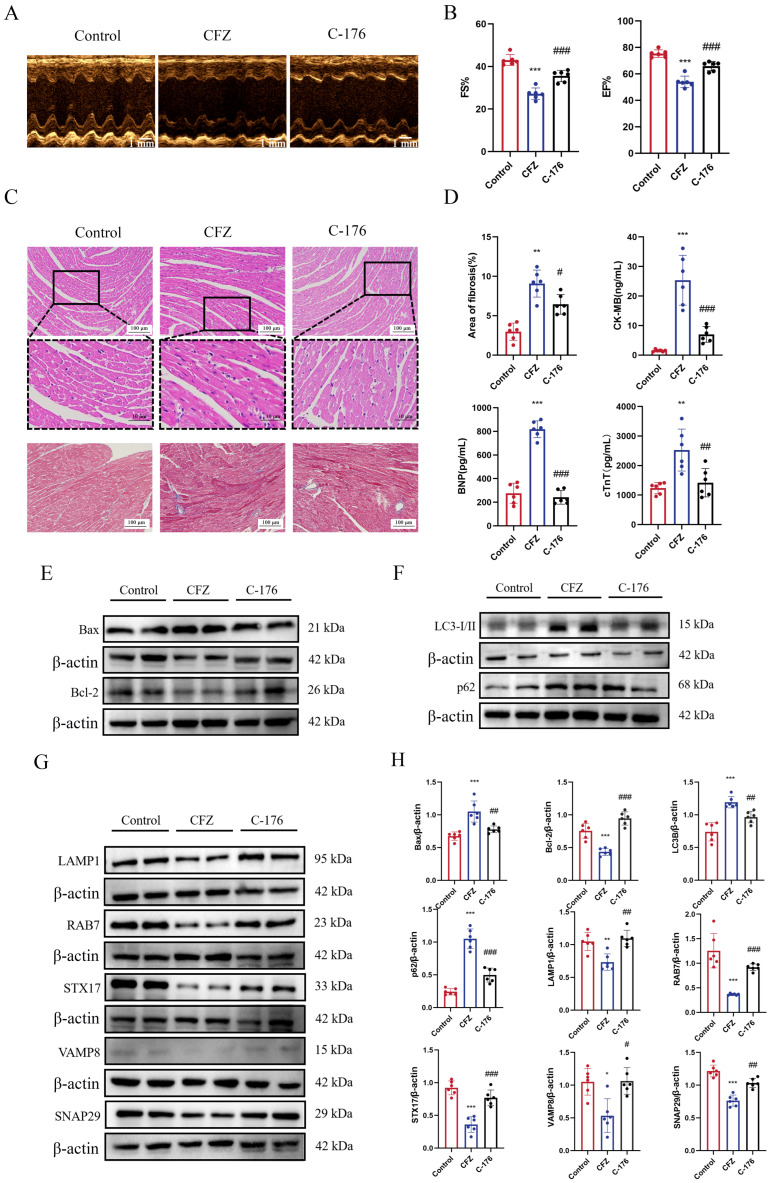
The use of C-176 as a STING inhibitor reversed the cardiotoxic effect of carfilzomib on C57BL/6J mice. (**A**) Echocardiographic results after the use of C-176 as a STING inhibitor (scale bar = 1 mm). (**B**) Statistical graph of the shortening fraction and ejection fraction. (**C**) H&E and Masson staining results (scale bar = 100 μm or 10 μm). (**D**) Statistical graph of Masson staining and serum content statistics of CK-MB, BNP, and cTNT. (**E**–**H**) Western blot results and statistical graphs of related proteins in the heart tissue of C57BL/6J mice after the use of C-176 as a STING inhibitor. Data represent the mean ± SD, *n* = 3, 6; * *p* < 0.05, ** *p* < 0.01, *** *p* < 0.001 vs. the control group; # *p* < 0.05, ## *p* < 0.01, ### *p* < 0.001 vs. the CFZ group.

As shown in Figure 9A,B, after the intraperitoneal injection of C-176, the FS% and EF% of the mice were significantly greater than those in the CFZ group. HE and Masson staining of mouse heart tissue revealed that pathological changes in mouse heart tissue were reduced and that the degree of fibrosis decreased; at the same time, the contents of CK-MB, BNP, and cTNT in the serum were detected, and those in the C-176 group were significantly lower than those in the CFZ group, indicating that inhibiting STING can reverse the cardiac damage caused by CFZ (Figure 9C,D). Finally, we conducted Western blot analysis of apoptosis-, autophagy-, autolysosome- and SNARE-related proteins, and the results are shown in Figure 9E–H. After inhibiting STING in vivo, apoptosis was inhibited, the expression of LC3 and p62 decreased, and autolysosome and SNARE protein expression was restored (*p* < 0.05, *p* < 0.01, or *p* < 0.001).

## 4. Discussion

Cardiomyocytes, as permanent cells, have higher proteasome activity and a higher protein turnover rate than other tissues [12]. In MM cells, proteasome inhibition leads to the rapid accumulation of incompatible regulatory proteins in the endoplasmic reticulum, which ultimately causes an unfolded protein response and induces an apoptotic cascade [13]. Similarly, in cardiomyocytes, the inhibition of proteasome-dependent sarcomere protein turnover can lead to protein imbalance, causing the abnormal accumulation of ubiquitinated proteins [14], thereby causing cellular dysfunction, leading to cell damage, and ultimately resulting in cysteine protease-mediated apoptosis and death [15]. Carfilzomib has a direct effect on the proteasome, and in studies on aged mice, carfilzomib can cause a decrease in vascular contraction and an increase in ROS release [16].

When heart function is impaired, it will cause abnormalities in the contraction and relaxation functions of the heart, thereby affecting the ejection fraction (EF%) and shortening fraction (FS%) [17]. In addition, when myocardial injury occurs, the activity levels of CK-MB and BNP in the body significantly increase [18]. cTNT is a myocardial-specific structural protein that rapidly increases and persists for a period of time within several hours after myocardial injury and is an important landmark indicator in diagnosing myocardial injury in clinical practice [19]. The results of this study indicate that carfilzomib can induce myocardial damage in mice. Similarly, doxorubicin and sunitinib also exhibit cardiotoxicity [20,21].

Previous studies have shown that myocardial injury can lead to the apoptosis of myocardial cells [22]. Apoptosis is proportional to the degree of impaired cardiac function [23]. The drug pretreatment can reduce myocardial cell apoptosis after ischemia–reperfusion by increasing the ratio of Bcl-2/Bax [24]. The Caspase family represents key cysteine proteases in the apoptosis process, among which Caspase-3 is an important protein [25] that can degrade DNA damage repair enzymes and activate nucleases, ultimately inducing apoptosis [26]. In this study, we used TUNEL staining and Western blot analysis and found that the green fluorescence in myocardial tissue was significantly increased. Moreover, the Western blot results revealed that the expression of Bax in the CFZ group was increased, the expression of Bcl2 was decreased, and the expression of cleaved caspase-3 was increased. The results at AC16 (with a 1 μM intervention for 24 h) were consistent with those from the in vivo experiments. However, this study only utilized the immortalized cell line AC16 and lacked research on NRCMs or iPSC-derived cardiomyocytes. Therefore, the findings need to be interpreted with caution and should be verified in more physiologically relevant models.

Mitochondrial damage or dysfunction can induce apoptosis [27]. Cardiomyocytes mainly obtain energy through mitochondrial oxidative phosphorylation [28,29]. The results showed that carfilzomib decreased mitochondrial membrane potential in a concentration-dependent manner, and transmission electron microscopy revealed mitochondrial swelling and the disappearance of cristae.

Established indicators of autophagic flux include LC3, p62, and the mCherry-GFP-LC3 reporter system. According to the literature, impaired autophagic flux is typically characterized by the accumulation of LC3-II and defective p62 degradation. In parallel, the mCherry-GFP-LC3 reporter visually reflects a block in autophagosome–lysosome fusion, evidenced by an increase in yellow puncta (autophagosomes) and a decrease in red puncta (autolysosomes). Therefore, our conclusions are based on these widely accepted criteria. We have now added a statement in the Discussion acknowledging that direct measurements of lysosomal pH and proteolytic activity were not performed, which we have noted as a study limitation. It was found through mCherry-GFP-LC3 adenovirus infection of AC16 cells that autophagy was activated, but the autophagic flux was blocked. Similar mechanisms have also been reported in ischemic stroke and porcine epidemic diarrhea virus, among other diseases [30,31,32]. In subsequent experiments, we explored how carfilzomib activates autophagy and blocks autophagic flux.

Autophagy is a lysosome-mediated degradation process, and it is closely related to a variety of diseases [33,34]. LC3 is a widely used marker of autophagy. P62, acting as a bridge between LC3 and polyubiquitinated proteins, is degraded by autophagic lysosomes [35]. In this study, the results showed that after the administration of carfilzomib, the expression of both LC3 and p62 increased. The increase in LC3 may be due to the activation of autophagosomes, whereas the increase in p62 levels is likely due to the blockage of autophagic flux. Professor Zeng Hui’s team discovered that inhibiting autophagy can weaken the cardiotoxicity induced by doxorubicin and further confirmed in vivo that SAR405 combined with right levocarnitine inhibits this cardiotoxicity, opening up new possibilities for the clinical treatment of cardiotoxicity induced by doxorubicin chemotherapy [36]. The expressions of the autophagy initiation proteins ATG5, Beclin-1 and ULK1 all increased after treatment with carfilzomib, indicating that autophagy initiation was activated [37,38,39,40,41,42].

The current prevailing view is that dysfunction in the autophagy–lysosome system is one of the most important mechanisms behind the obstruction of autophagic flux and is also a pathogenic factor for various diseases [43]. Recent studies have shown that the myocardial infarction model induced by isoproterenol also involves lysosomal dysfunction [44]. When lysosomal function is impaired, and autophagic flux is blocked, the expression of both LAMP1 and RAB7 decreases [45]. The protein expression level of LAMP1 in the carfilzomib group decreased, as did the protein expression of RAB7. The combination of autophagosomes and lysosomes to form autophagic lysosomes requires the cooperative action of the SNARE complex (STX17, SNAP29, VAMP8) [46,47]. Reduced expression of STX17, VAMP8, and SNAP29 damages the formation of autophagic lysosomes [48,49,50].

Studies have shown that enhancing the activity of the 20S proteasome significantly increases the degradation of SNAP29 and STX17 but has no significant effect on VAMP8 levels. Further research has elucidated that this degradation occurs through a ubiquitin-independent pathway and is primarily mediated by the 20S core particle in the absence of the 19S regulatory particle. SNAP29 and STX17 are responsible for mediating autophagosome–lysosome fusion during autophagy. Enhanced 20S proteasome activity leads to their excessive degradation, thereby significantly reducing autophagic flux. Through Western blot experiments, we found that carfilzomib could inhibit the protein expression of STX17, SNAP29, and VAMP8, thereby inhibiting the formation of autophagic lysosomes.

Studies have shown that various risk factors can activate the cGAS-STING pathway and participate in cardiovascular pathological damage and that the abnormal activation of the cGAS-STING pathway is associated with several cardiac functional disorders [51]. In heart diseases, the cGAS-STING pathway is overactivated, leading to an excessive release of IFN-1, IL-6, and other inflammatory factors, which may be one of the reasons for the aggravation of heart diseases [52]. Professor Chen Zhijian’s team discovered that STING can induce autophagy [53]. Professor Xu Pinglong’s team reported through experiments that the STING protein mediates the nonclassical signal transduction pathway on the endoplasmic reticulum membrane [54]. Therefore, we speculate that the effect of carfilzomib-induced cardiotoxicity may be related to the cGAS-STING pathway. Furthermore, the formation of the STING-SNARE complex is closely related: Knocking out *STX17* will block the autophagic flux and exacerbate atherosclerosis, while knocking out *STING* can enhance the autophagic flux [55].

The results of in vitro and in vivo experiments revealed that carfilzomib treatment could activate the cGAS–STING pathway. Moreover, when si*STING* was used to knock down *STING* in AC16 cells and C-176 was used as a STING inhibitor in C57BL/6J mice, the protein expression levels of LC3 and p62 decreased, the protein expression levels of LAMP1 and RAB7 increased, and the protein expression levels of the SNARE complex-related proteins STX17, VAMP8, and SNAP29 increased. Autophagic lysosomal function was restored, indicating that after *STING* was knocked down, autophagic flux recovered.

At the same time, inhibition of STING also reduces tumor burden. Specifically in multiple myeloma, studies have shown that inhibiting the STING pathway reduces tumor burden in preclinical models. Furthermore, knocking down *STING* in multiple myeloma cells has been reported to promote colony formation, suggesting that the role of STING may be highly context-dependent and that under certain circumstances, STING suppression may be therapeutically beneficial rather than detrimental. On the other hand, the ratio of Bax/Bcl-2 decreased, as did apoptosis, indicating that inhibiting the cGAS-STING pathway can reduce the cardiotoxicity induced by carfilzomib. Future studies employing qRT-PCR validation in independent cohorts and mechanistic dissection of STING-mediated SNARE degradation will be required to fully resolve the molecular pathways involved.

## 5. Conclusions

**Figure 10:**
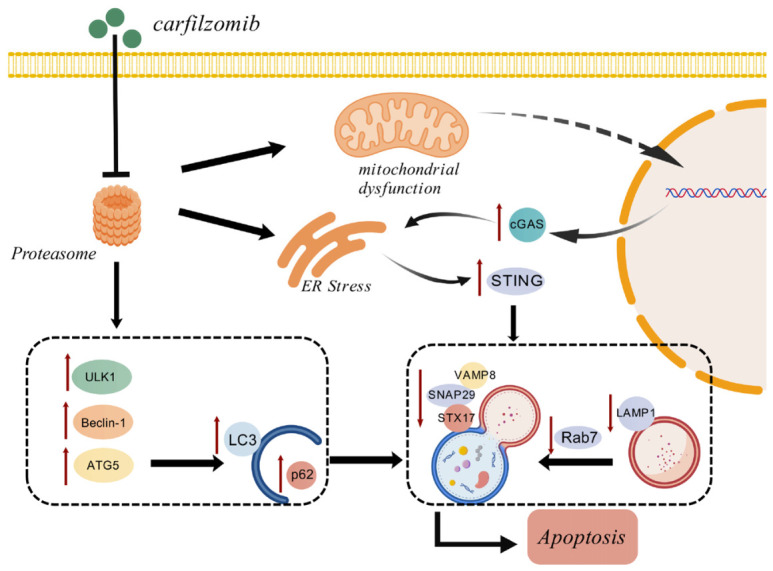
Carfilzomib (CFZ) treatment induces mitochondrial damage in cardiomyocytes, which may cause mitochondrial DNA (mtDNA) to be released into the cytoplasm. This triggers the activation of the cGAS-STING signaling pathway. Once activated, STING suppresses the expression of the SNARE complex (STX17-SNAP29-VAMP8), which is essential for the fusion of autophagosomes with lysosomes. Consequently, autophagic flux is disrupted at the fusion stage, resulting in the accumulation of autophagosomes that fail to form autophagolysosomes. This blockade impairs the clearance of damaged mitochondria and other organelles, ultimately contributing to cardiomyocyte apoptosis and cardiac dysfunction.

Carfilzomib can damage mitochondria and activate the formation of autophagosomes through the cGAS-STING signaling pathway. It also promotes the formation of autophagosomes while inhibiting the formation of autolysosomes, thereby blocking autophagic flux and ultimately leading to the accumulation of damaged mitochondria and the induction of apoptosis. However, direct detection of cytosolic mtDNA has not been performed, and future studies are required to validate this proposed mechanism (Figure 10).

## Data Availability

The paper contains the original contributions made during the study; further inquiries can be directed to the corresponding authors.

## Supplementary Materials

The following supporting information can be downloaded at: https://www.mdpi.com/article/10.3390/biom16060854/s1, Figure S1: (A) Statistical graph of changes in STX17, SNAP29 and VAMP8 mRNA expression. (B) Verification by Western Blot after STING knockdown (n = 3). (C) Weight changes of mice after injection of CFZ (n = 15). Data represent the mean ± SD; ** *p* < 0.01, *** *p* < 0.001 vs. the control group; Figure S2: (A) ViolinPlot. (B) DensityPlot. (C) PCA. (D) SampleCorrelation groupHeatmap.

## Abbreviations

The following abbreviations are used in this manuscript:

ATG5	Autophagy-related gene 5
BNP	Brain natriuretic peptide
CFZ	Carfilzomib
cGAS	Cyclic GMP–AMP synthase
CK-MB	Creatine kinase MB
cTNT	Cardiac troponin T
CVAE	Cardiovascular adverse events
EF	Ejection Fraction
FS	Fractional shortening
GSEA	Gene set enrichment analysis
MM	Multiple myeloma
