# Supplementary material for: Carfilzomib Induces Cardiotoxicity by Blocking Autophagic Flux Through the cGAS-STING Signaling Pathway
Source: Biomolecules. 2026 Jun 11;16(6):854. doi: 10.3390/biom16060854 (PMC13297306; doi:10.3390/biom16060854)
Supplement: Supplementary file 1 [file biomolecules-16-00854-s001.zip › biomolecules-4233931-original-images.pdf]

Figure 2

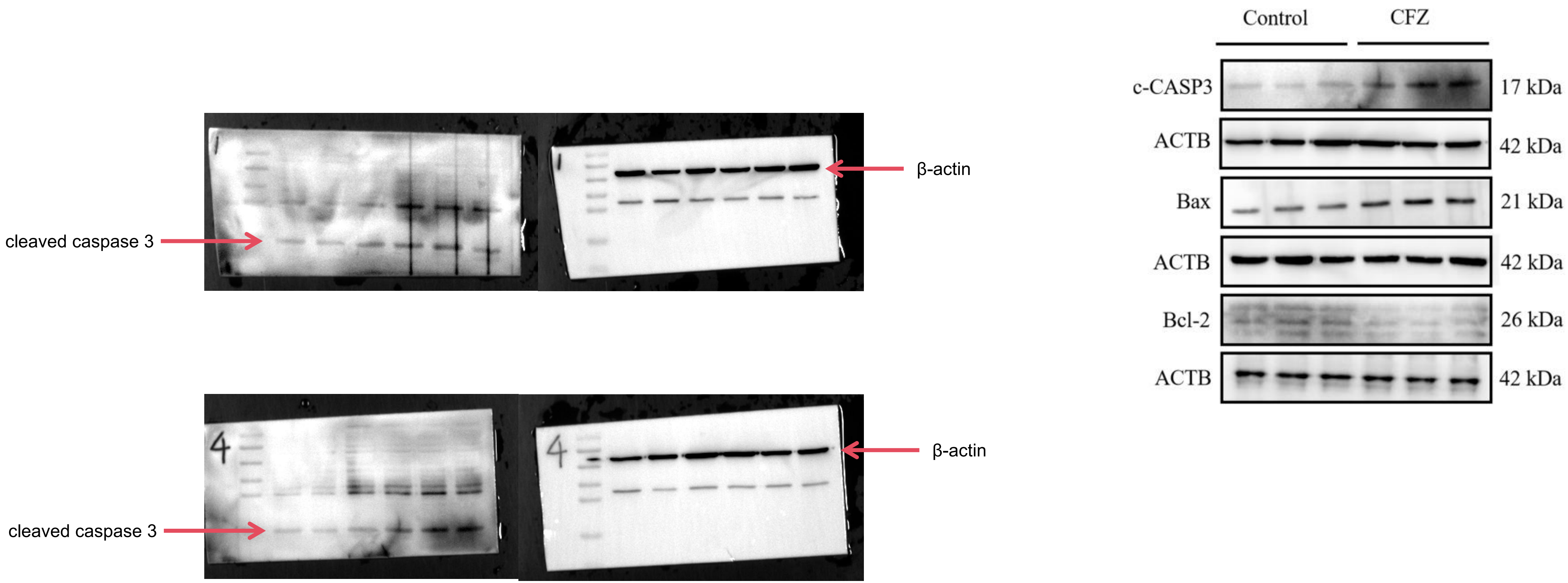
cleaved caspase 3
β-actin
cleaved caspase 3
β-actin
Control
CFZ
c-CASP3
17 kDa
ACTB
42 kDa
Bax
21 kDa
ACTB
42 kDa
Bcl-2
26 kDa
ACTB
42 kDa

Figure 2

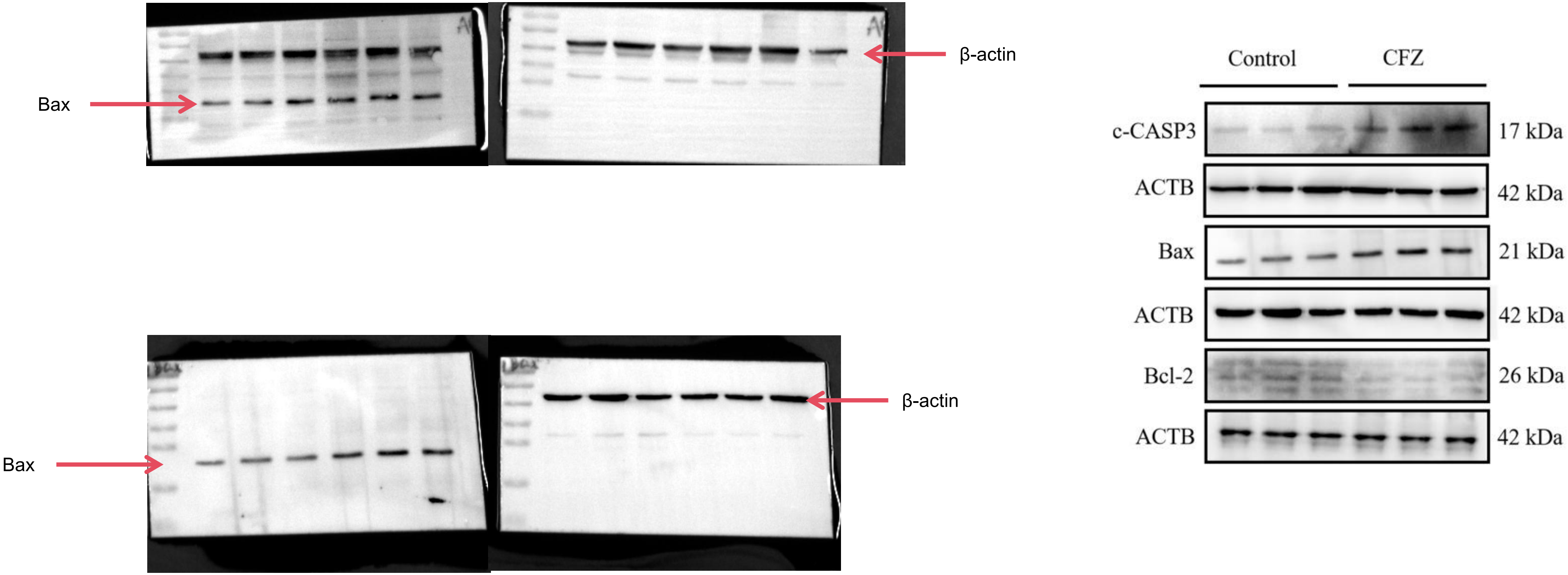
Bax
β-actin
Bax
β-actin
Control
CFZ
c-CASP3
17 kDa
ACTB
42 kDa
Bax
21 kDa
ACTB
42 kDa
Bcl-2
26 kDa
ACTB
42 kDa

Figure 2

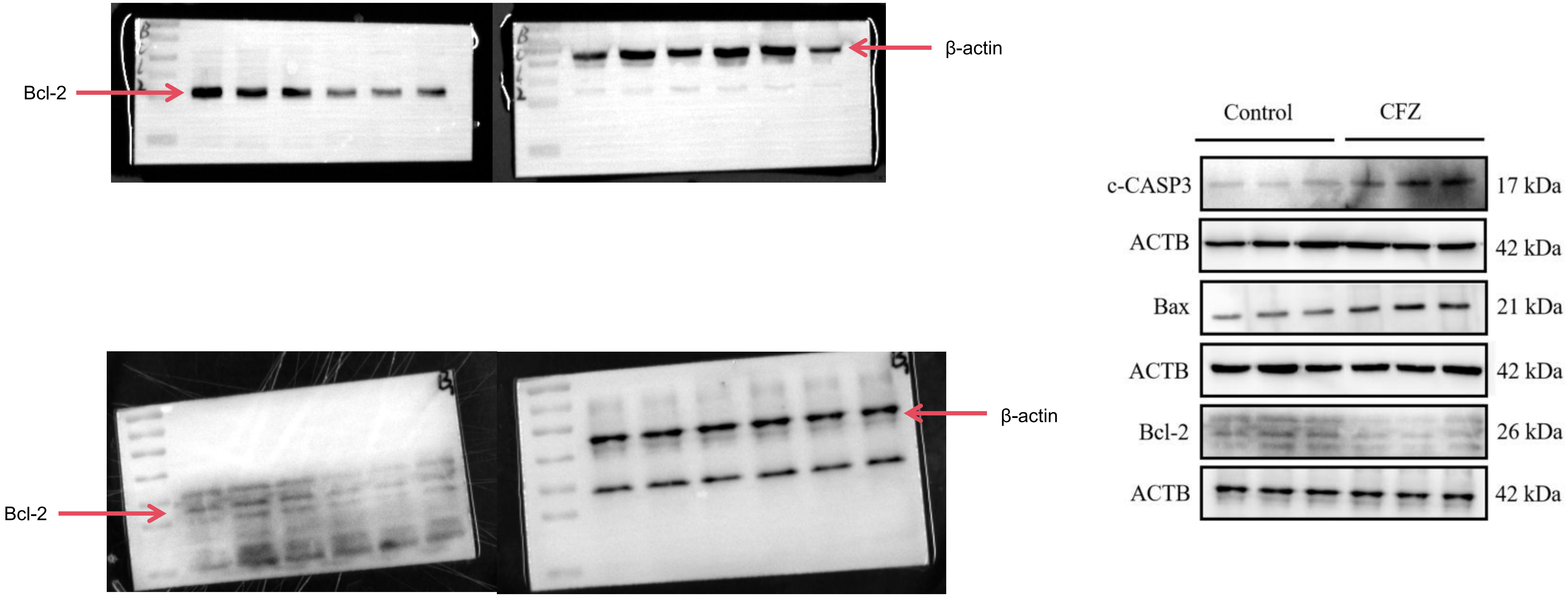

Bcl-2
β-actin
Bcl-2
β-actin
Control
CFZ
c-CASP3
17 kDa
ACTB
42 kDa
Bax
21 kDa
ACTB
42 kDa
Bcl-2
26 kDa
ACTB
42 kDa

Figure 3

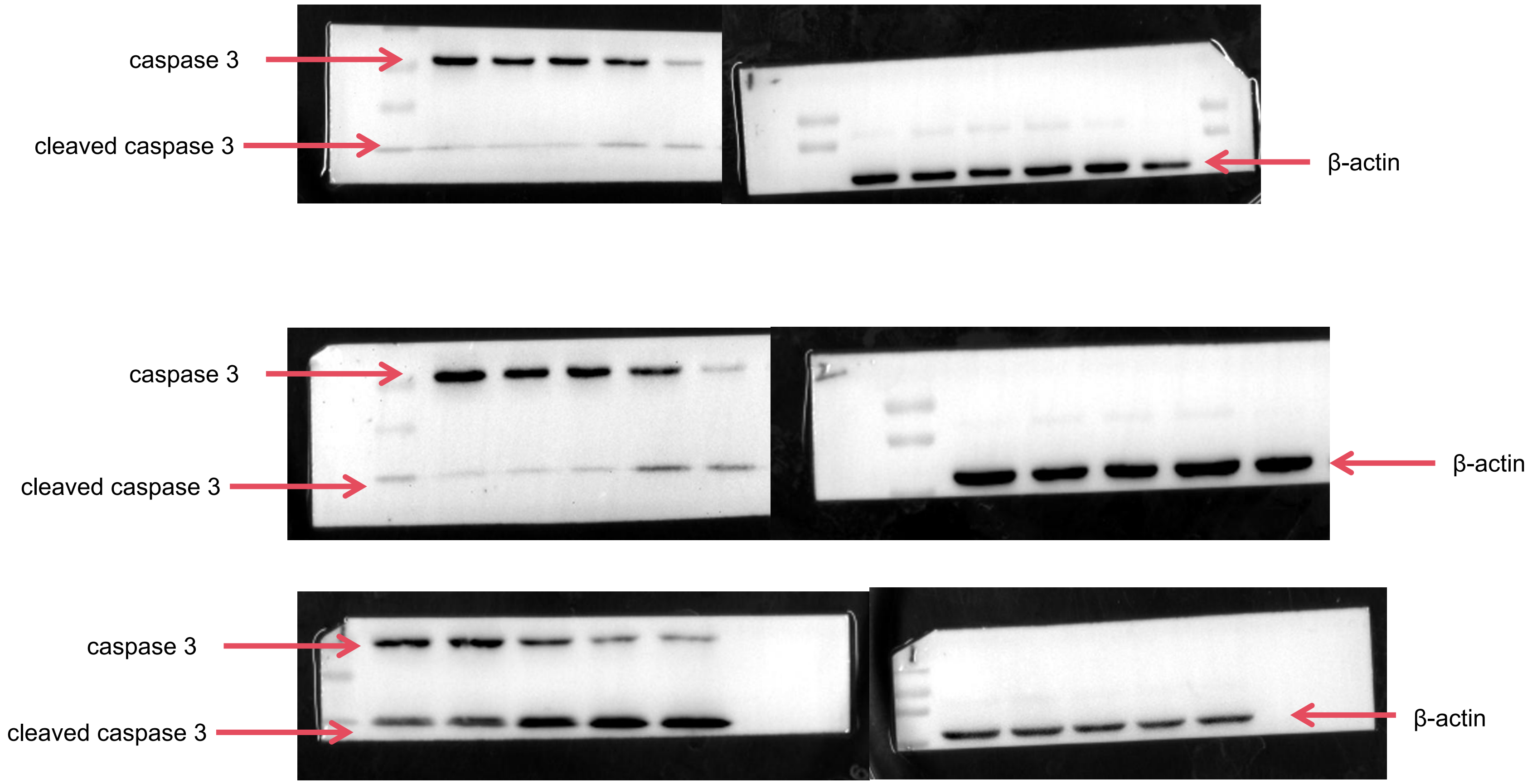
caspase 3
cleaved caspase 3
β-actin
caspase 3
cleaved caspase 3
β-actin
caspase 3
cleaved caspase 3
β-actin

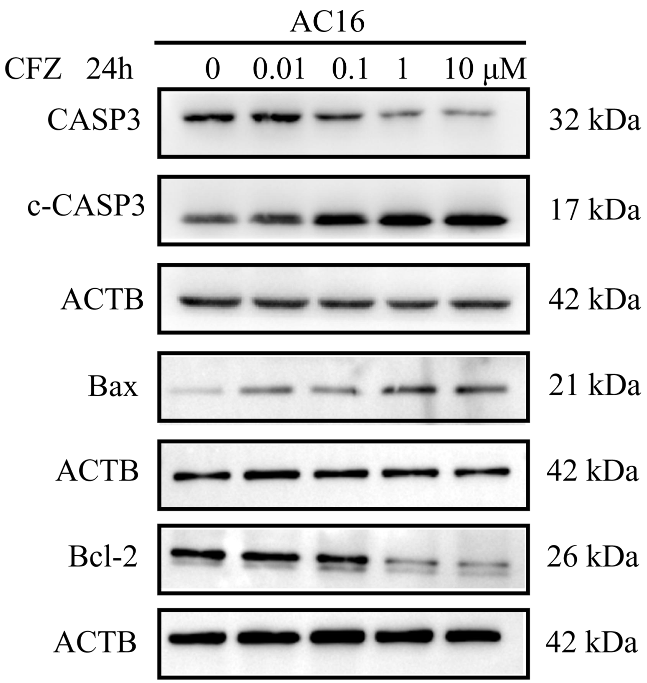
AC16
CFZ 24h
0 0.01 0.1 1 10 μM
CASP3
32 kDa
c-CASP3
17 kDa
ACTB
42 kDa
Bax
21 kDa
ACTB
42 kDa
Bcl-2
26 kDa
ACTB
42 kDa

Figure 3

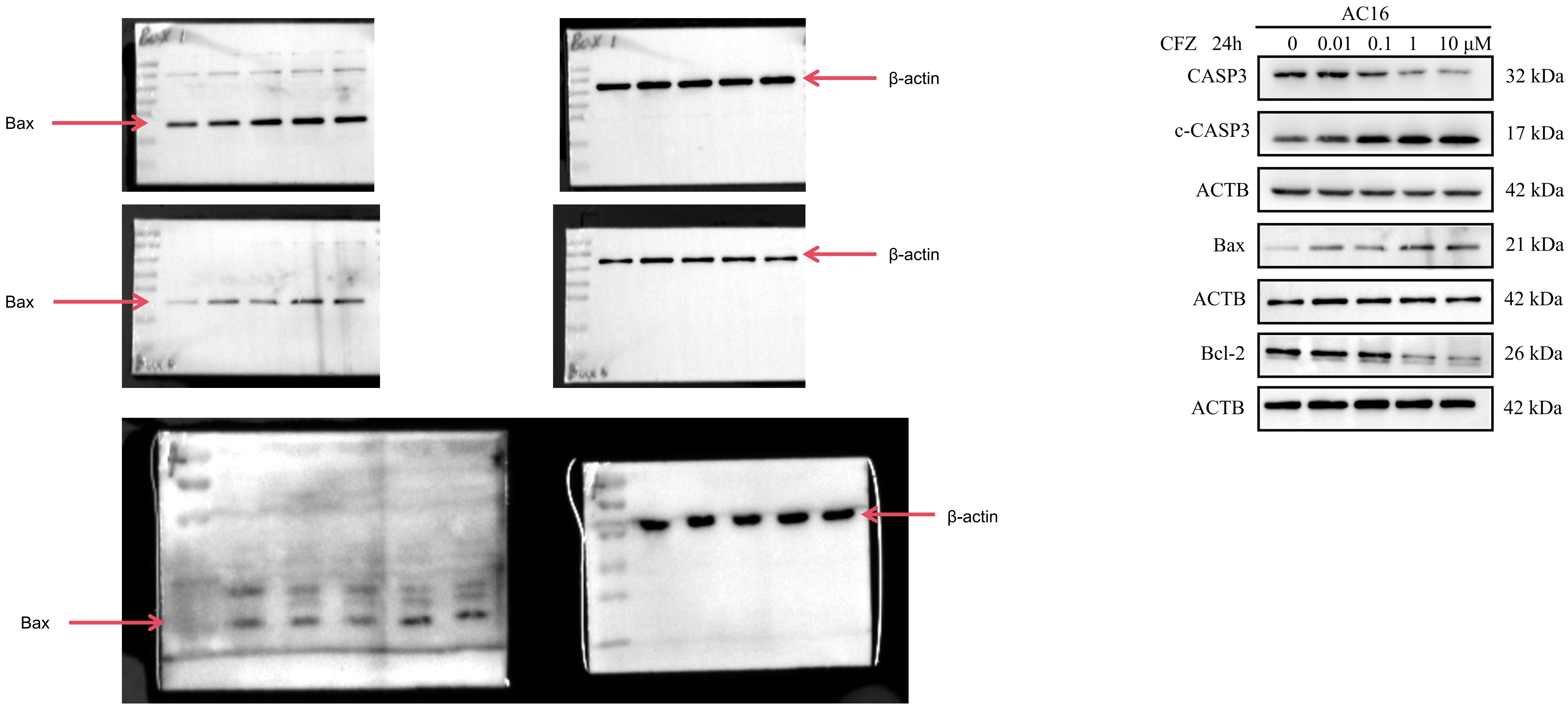
Bax
β-actin
Bax
β-actin
Bax
β-actin
AC16
CFZ 24h 0 0.01 0.1 1 10 µM
CASP3 32 kDa
c-CASP3 17 kDa
ACTB 42 kDa
Bax 21 kDa
ACTB 42 kDa
Bcl-2 26 kDa
ACTB 42 kDa

Figure 3

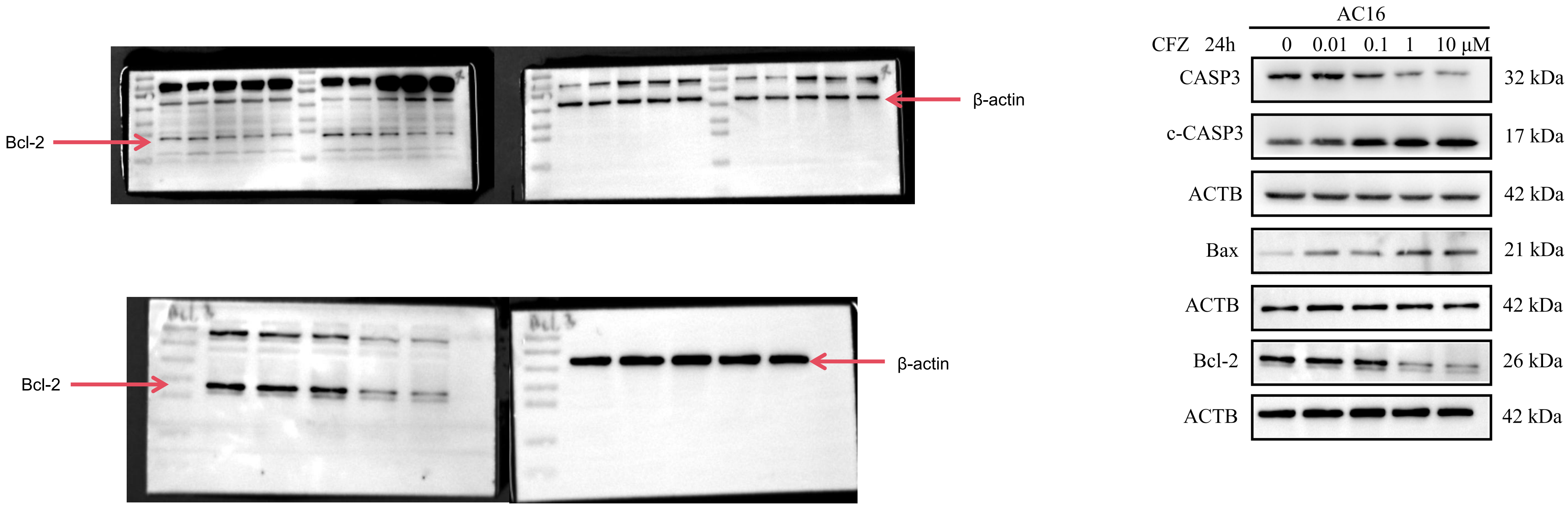
Bcl-2
β-actin
Bcl-2
β-actin
AC16
CFZ 24h
0
0.01
0.1
1
10 μM
CASP3
32 kDa
c-CASP3
17 kDa
ACTB
42 kDa
Bax
21 kDa
ACTB
42 kDa
Bcl-2
26 kDa
ACTB
42 kDa

Figure 6

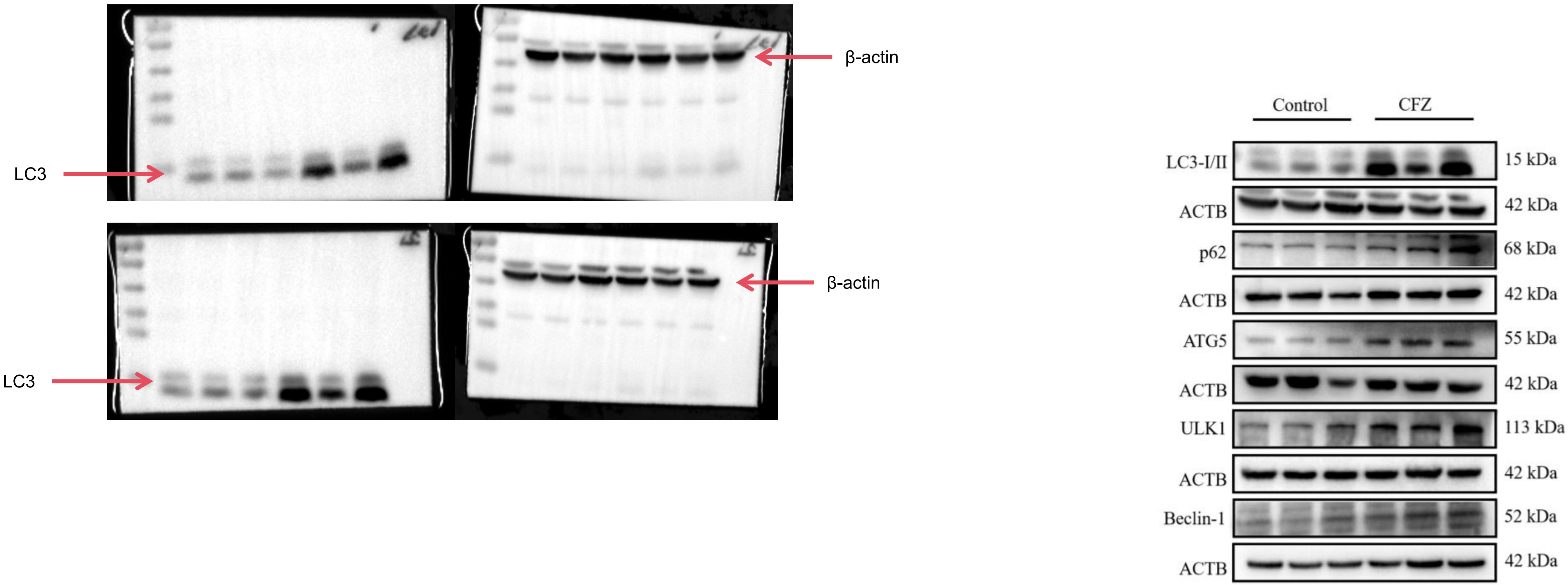
LC3
β-actin
LC3
β-actin
Control
CFZ
LC3-I/II
15 kDa
ACTB
42 kDa
p62
68 kDa
ACTB
42 kDa
ATG5
55 kDa
ACTB
42 kDa
ULK1
113 kDa
ACTB
42 kDa
Beclin-1
52 kDa
ACTB
42 kDa

Figure 6

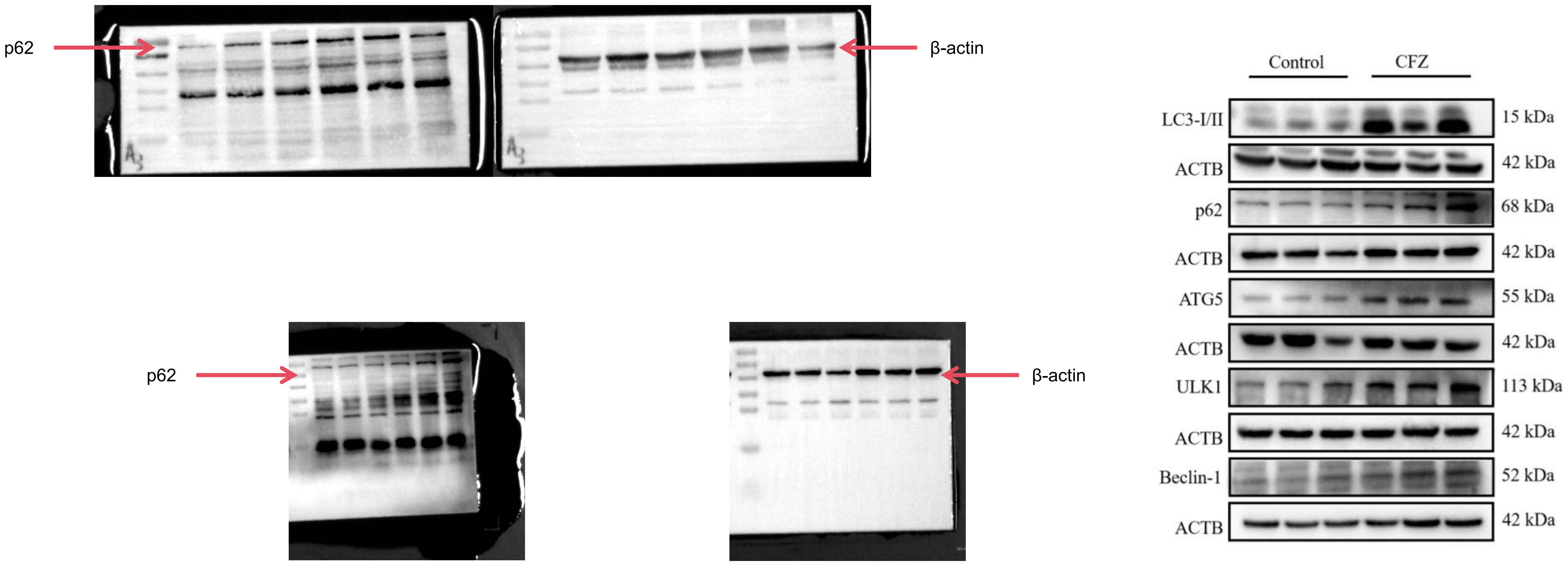
p62
β-actin
p62
β-actin
Control
CFZ
LC3-I/II
15 kDa
ACTB
42 kDa
p62
68 kDa
ACTB
42 kDa
ATG5
55 kDa
ACTB
42 kDa
ULK1
113 kDa
ACTB
42 kDa
Beclin-1
52 kDa
ACTB
42 kDa

Figure 6

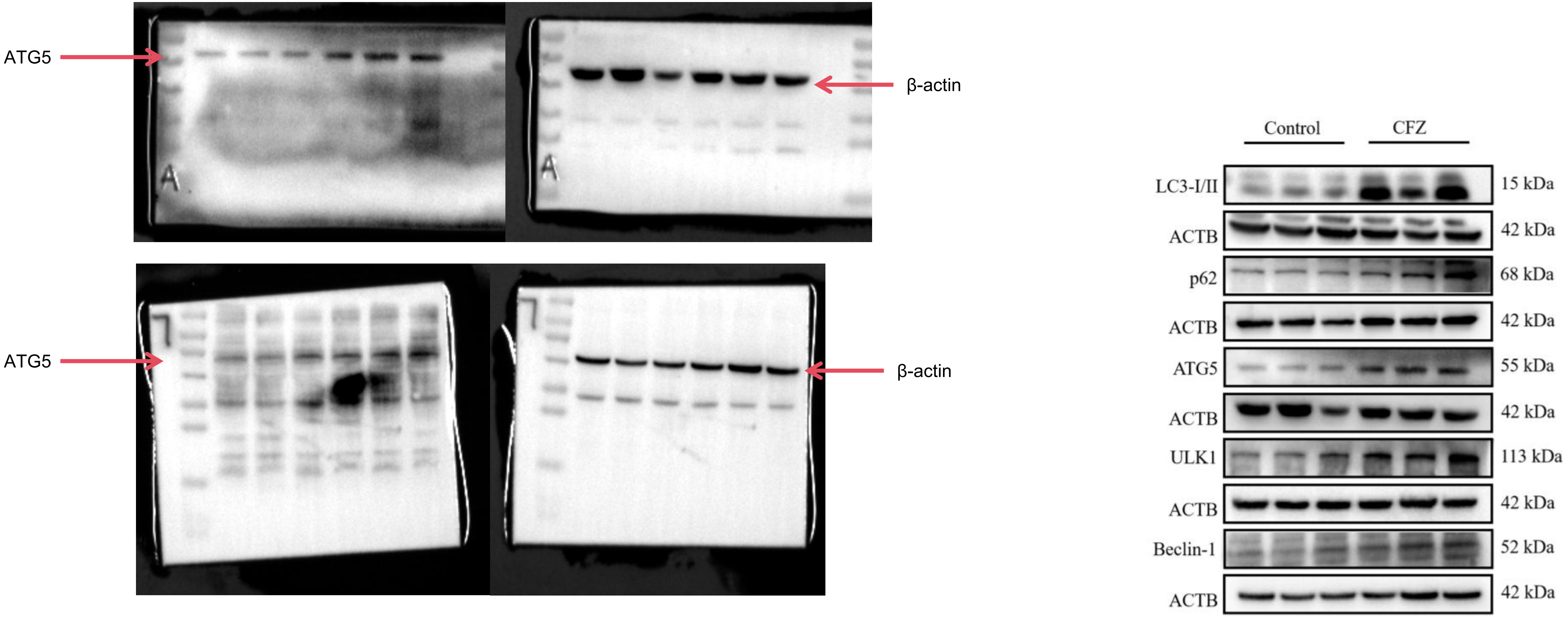

ATG5
β-actin
ATG5
β-actin
Control
CFZ
LC3-I/II
15 kDa
ACTB
42 kDa
p62
68 kDa
ACTB
42 kDa
ATG5
55 kDa
ACTB
42 kDa
ULK1
113 kDa
ACTB
42 kDa
Beclin-1
52 kDa
ACTB
42 kDa

Figure 6

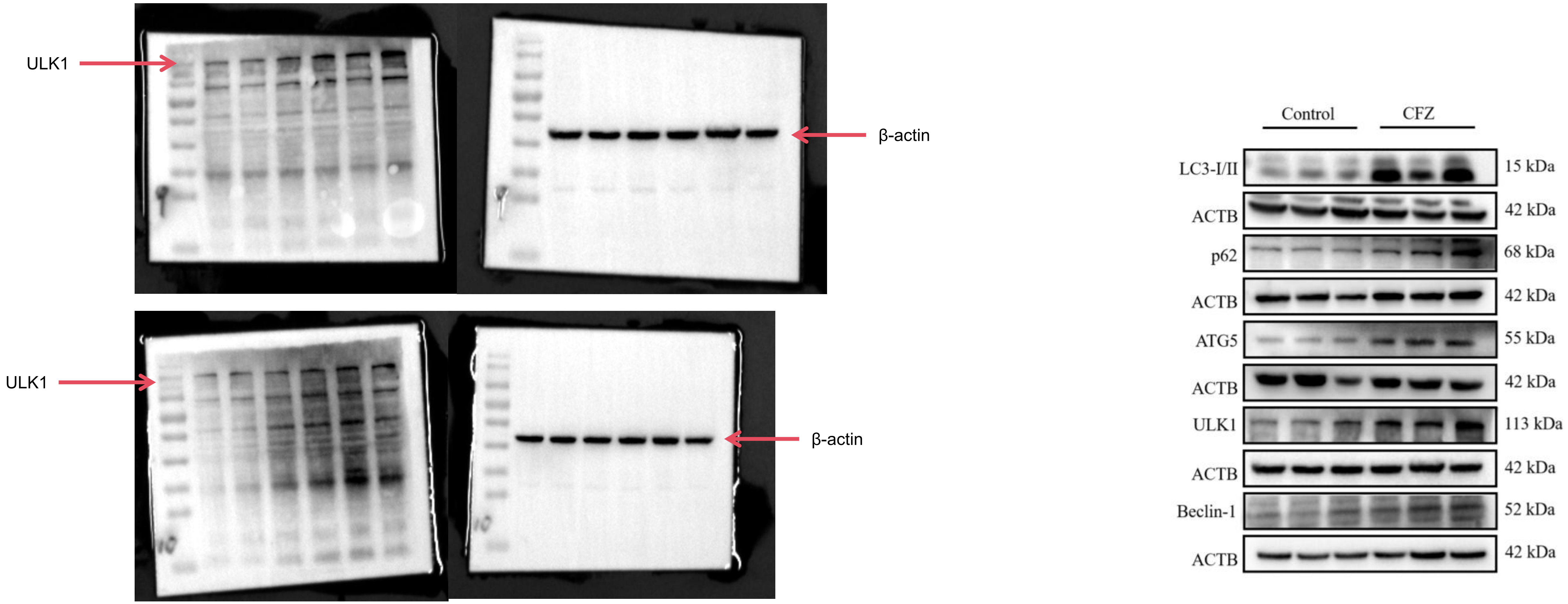
ULK1
β-actin
ULK1
β-actin
Control
CFZ
LC3-I/II
15 kDa
ACTB
42 kDa
p62
68 kDa
ACTB
42 kDa
ATG5
55 kDa
ACTB
42 kDa
ULK1
113 kDa
ACTB
42 kDa
Beclin-1
52 kDa
ACTB
42 kDa

Figure 6

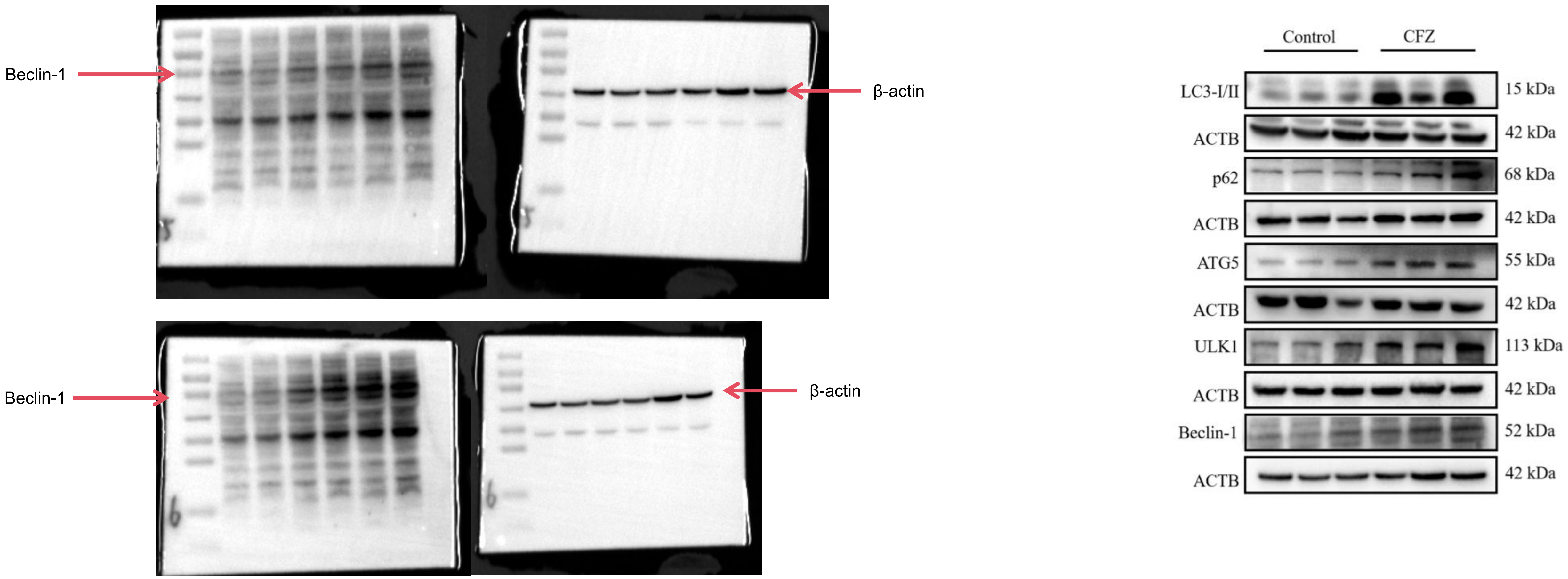

Beclin-1
β-actin
Beclin-1
β-actin
Control
CFZ
LC3-I/II
ACTB
p62
ACTB
ATG5
ACTB
ULK1
ACTB
Beclin-1
ACTB
15 kDa
42 kDa
68 kDa
42 kDa
55 kDa
42 kDa
113 kDa
42 kDa
52 kDa
42 kDa

Figure 6

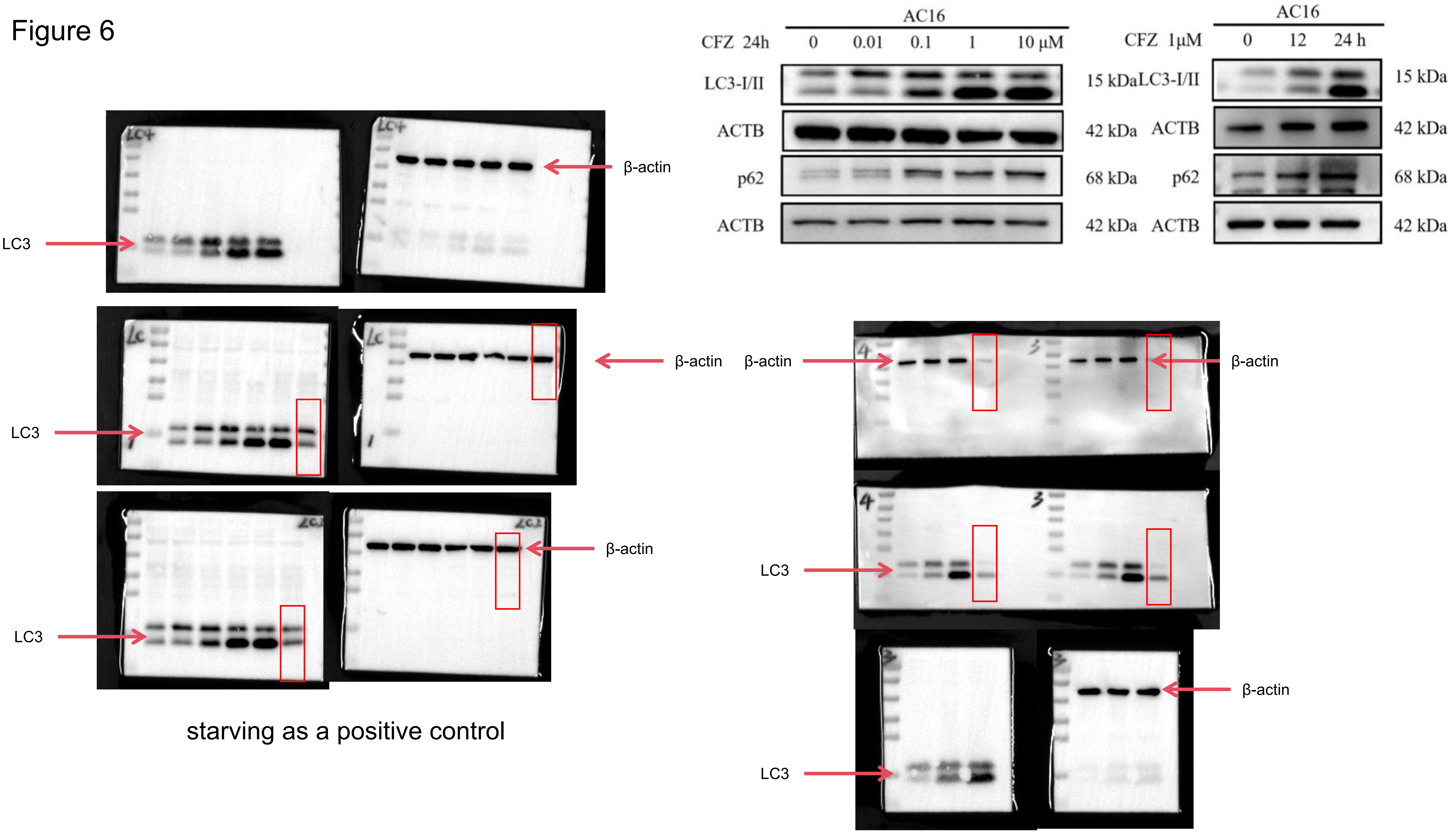

starving as a positive control

Figure 6

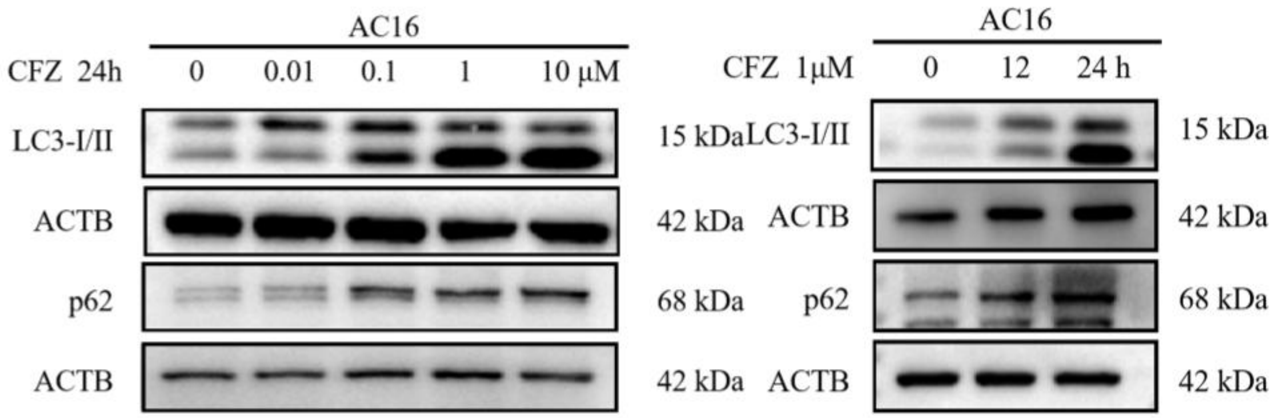
AC16
CFZ 24h
0
0.01
0.1
1
10 μM
LC3-I/II
ACTB
p62
ACTB
15 kDa
42 kDa
68 kDa
42 kDa
AC16
CFZ 1μM
0
12
24 h
LC3-I/II
ACTB
p62
ACTB
15 kDa
42 kDa
68 kDa
42 kDa

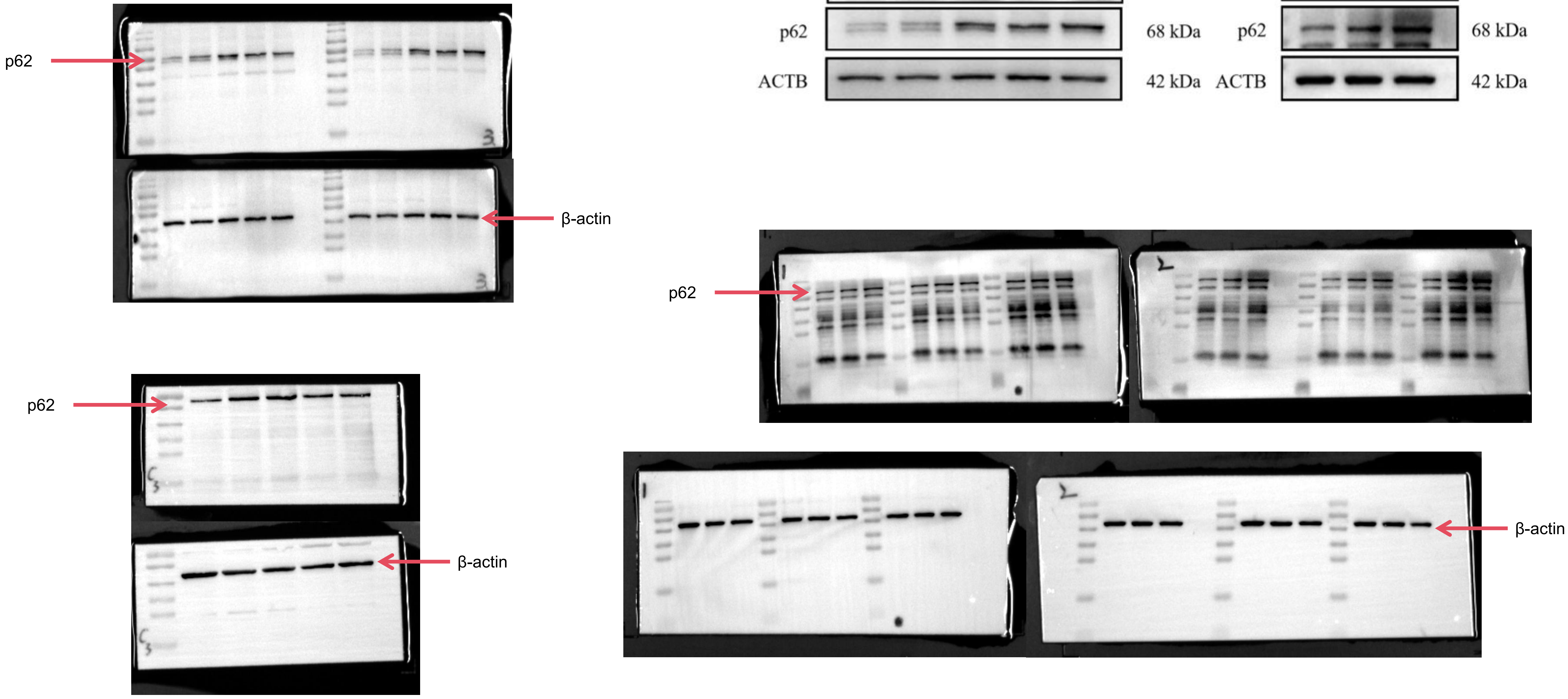
p62
β-actin
p62
β-actin
p62
β-actin

Figure 6

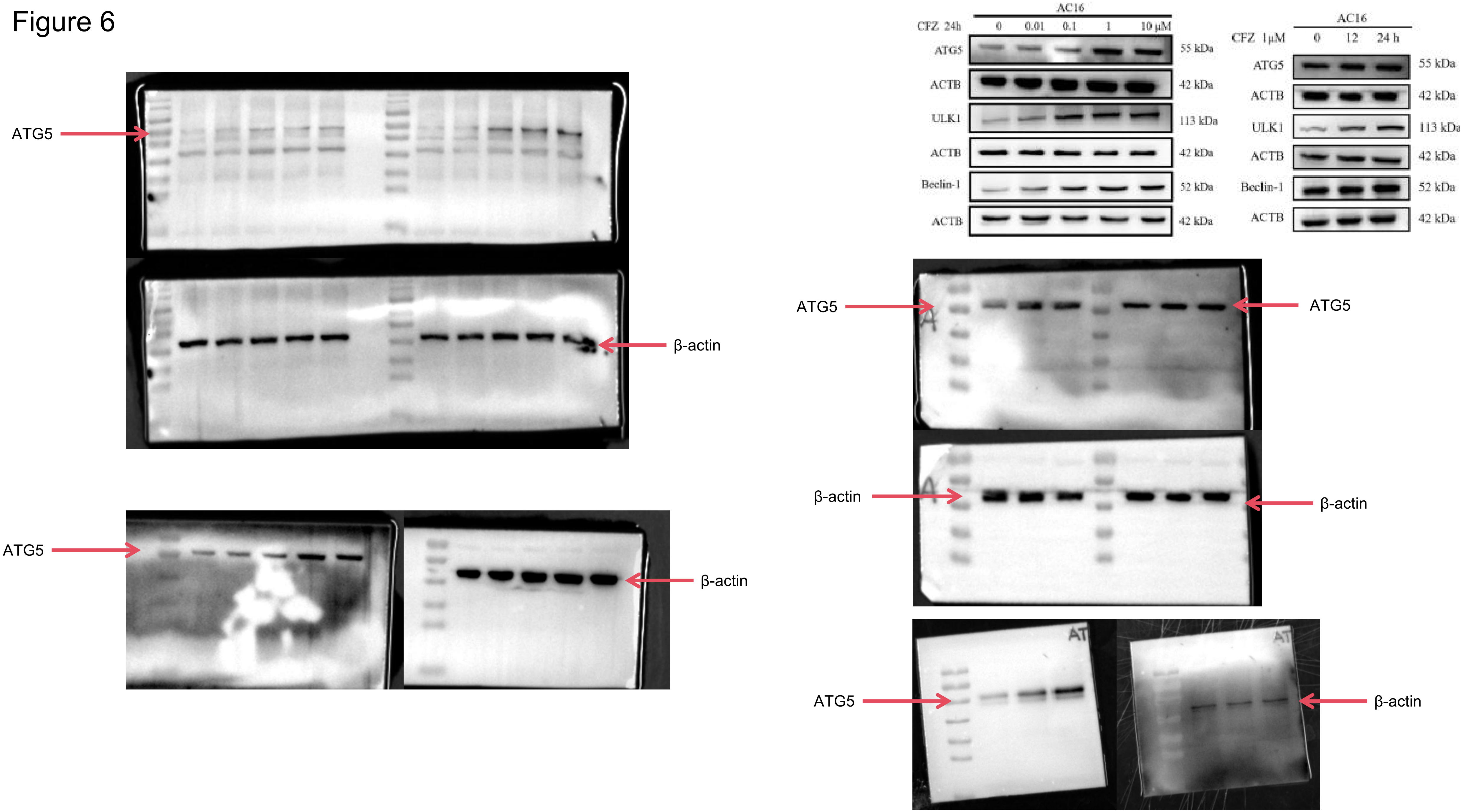
AC16
CFZ 24h
0
0.01
0.1
1
10 μM
ATG5
55 kDa
ACTB
42 kDa
ULK1
113 kDa
Beclin-1
52 kDa
CFZ 1μM
12
24 h
ATG5
β-actin

Figure 6

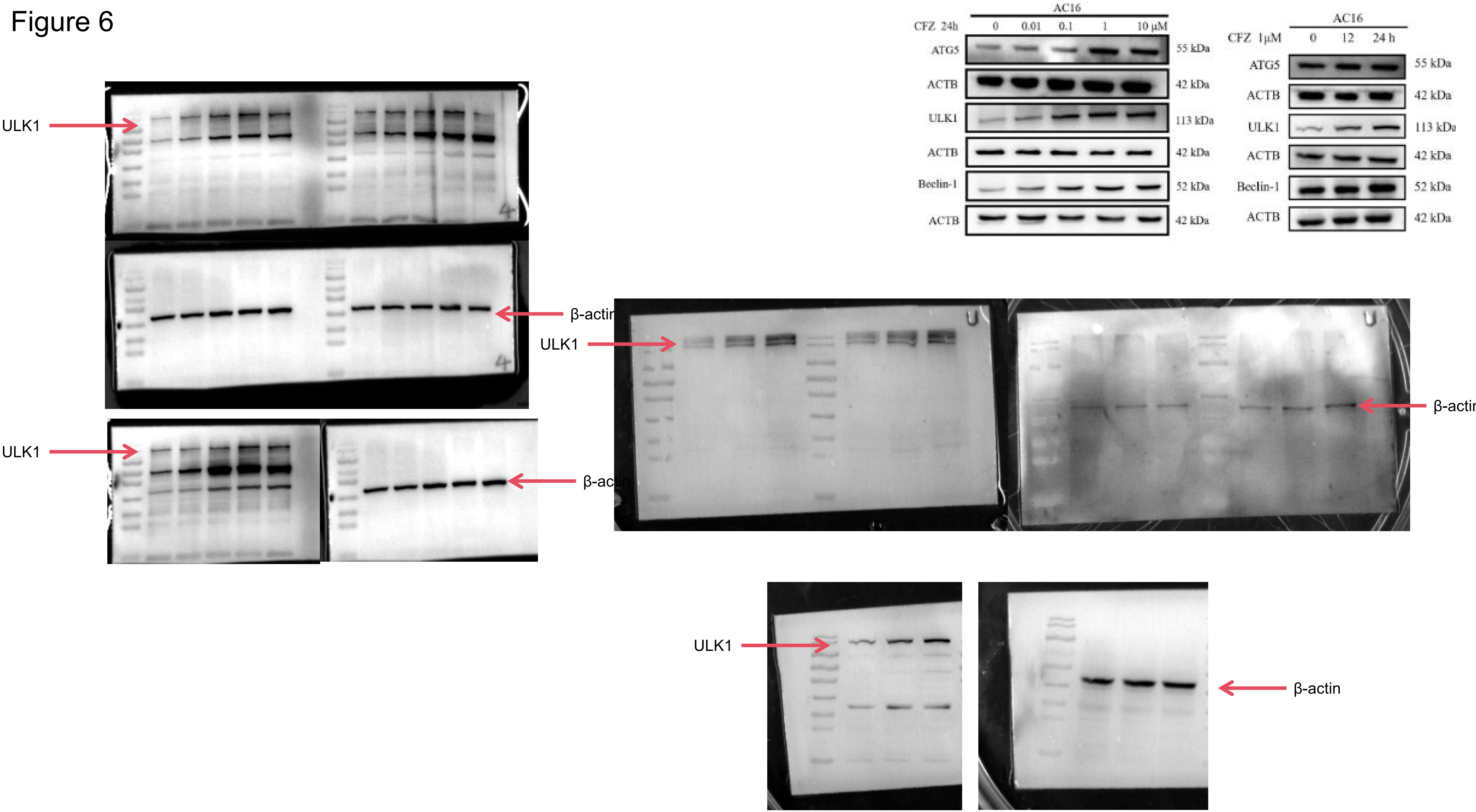
AC16
CFZ 24h
0
0.01
0.1
1
10 μM
ATG5
ACTB
ULK1
ACTB
Beclin-1
ACTB
55 kDa
42 kDa
113 kDa
42 kDa
52 kDa
42 kDa
AC16
CFZ 1μM
0
12
24 h
ATG5
ACTB
ULK1
ACTB
Beclin-1
ACTB
55 kDa
42 kDa
113 kDa
42 kDa
52 kDa
42 kDa
ULK1
β-actin
ULK1
ULK1
ULK1
β-actin

Figure 6

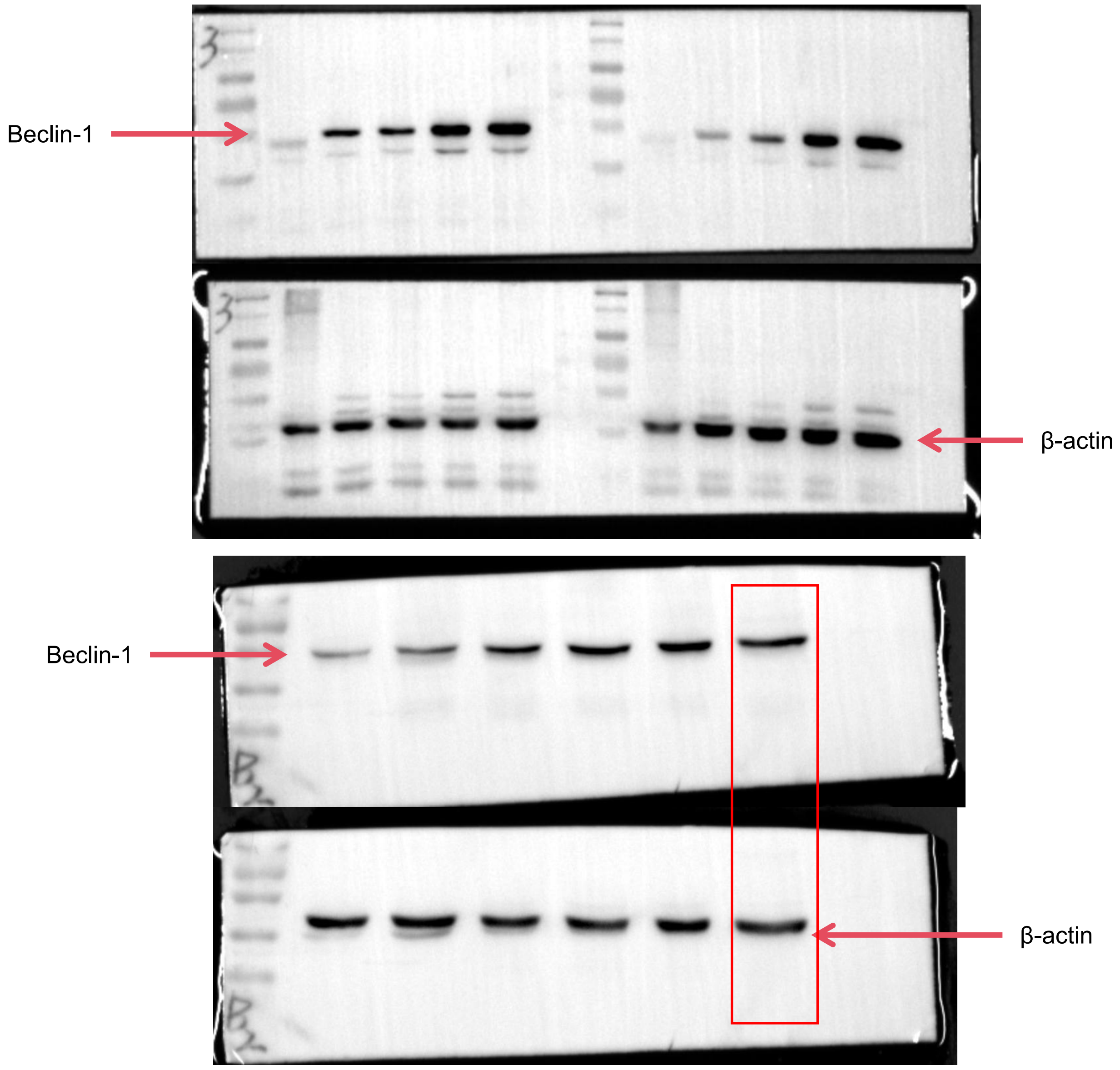

starving as a positive control

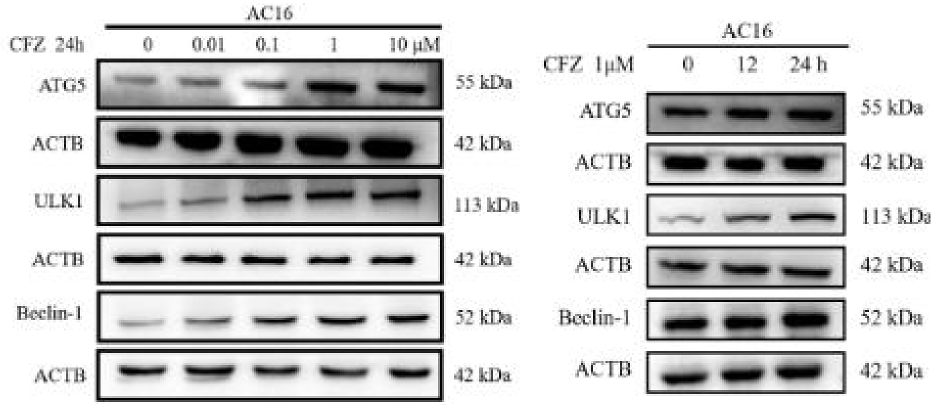

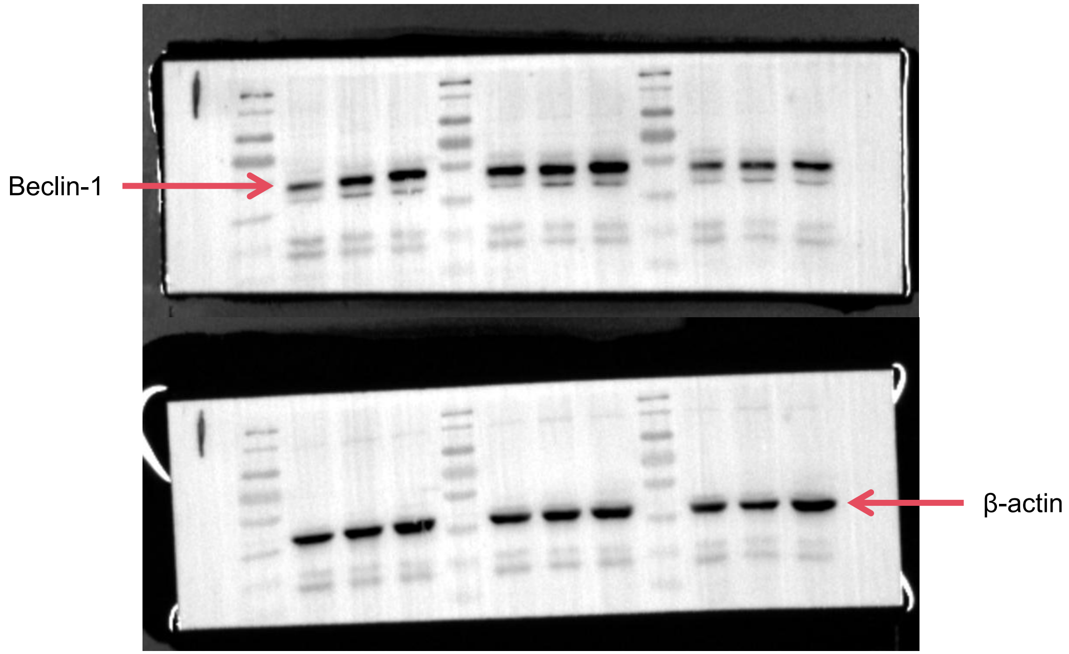

Figure 7

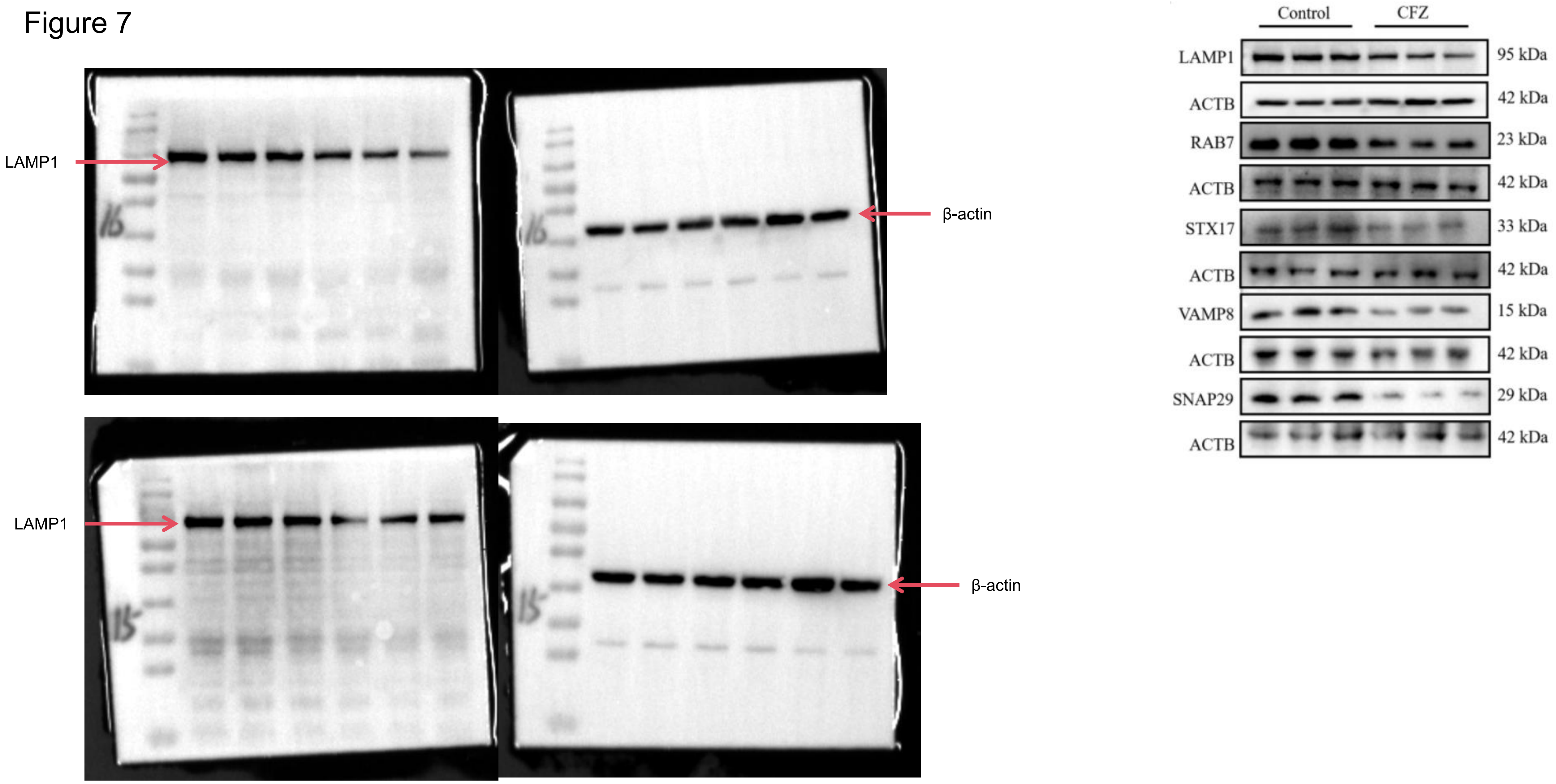

LAMP1
β-actin
LAMP1
β-actin
Control
CFZ
LAMP1
95 kDa
ACTB
42 kDa
RAB7
23 kDa
ACTB
42 kDa
STX17
33 kDa
ACTB
42 kDa
VAMP8
15 kDa
ACTB
42 kDa
SNAP29
29 kDa
ACTB
42 kDa

Figure 7

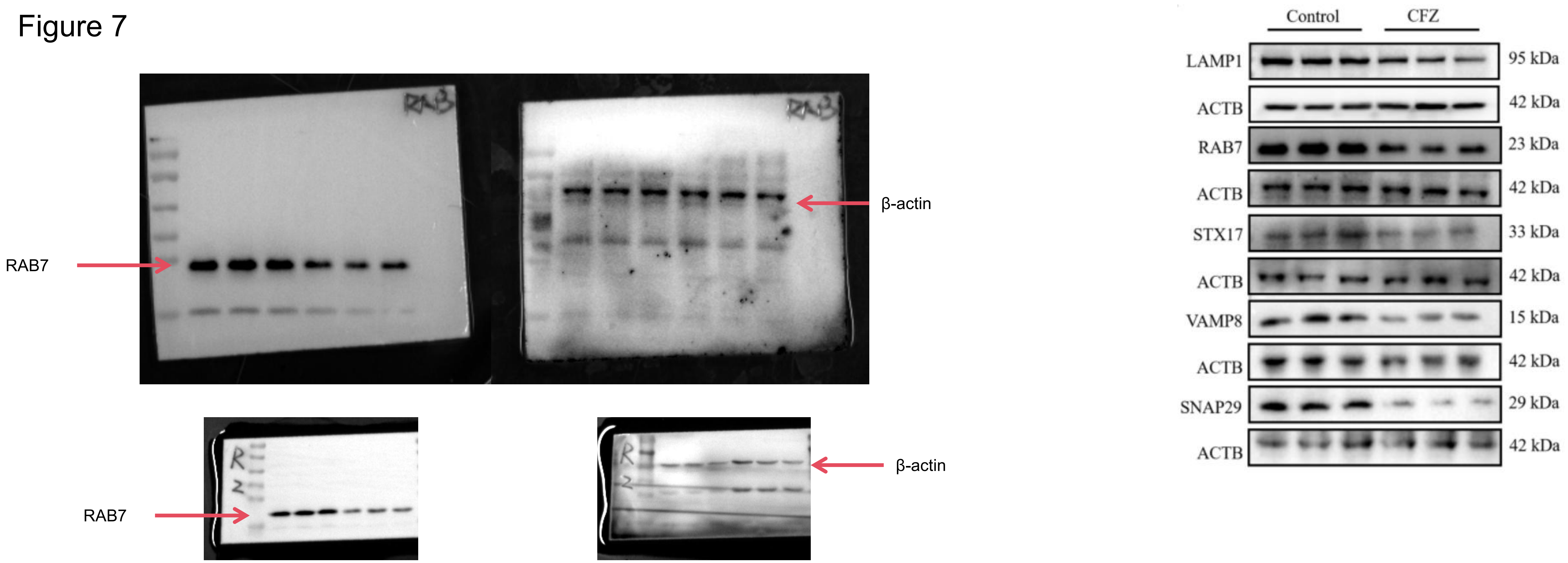

RAB7
β-actin
RAB7
β-actin
Control
CFZ
LAMP1
95 kDa
ACTB
42 kDa
RAB7
23 kDa
ACTB
42 kDa
STX17
33 kDa
ACTB
42 kDa
VAMP8
15 kDa
ACTB
42 kDa
SNAP29
29 kDa
ACTB
42 kDa

Figure 7

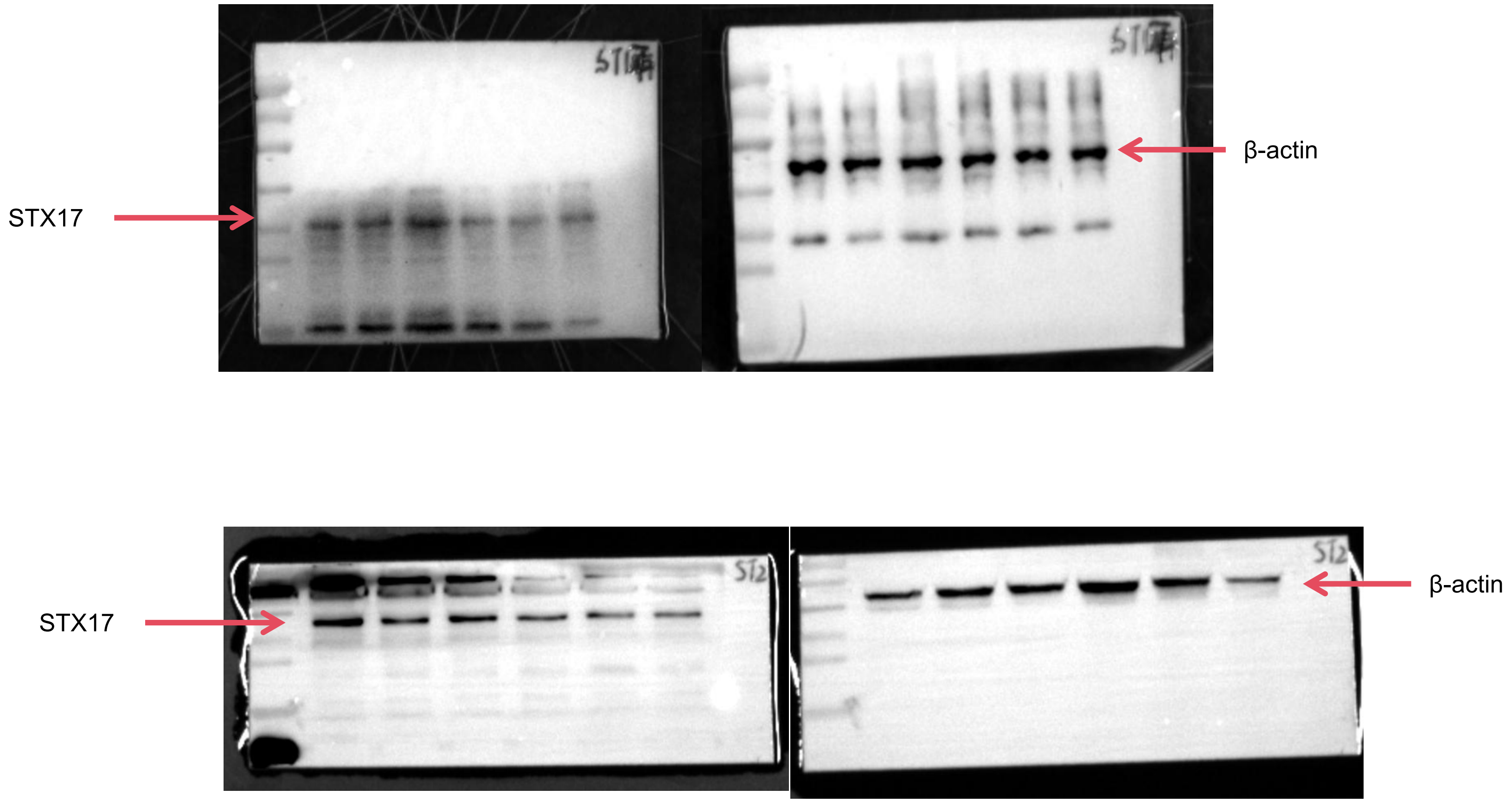
STX17
β-actin
STX17
β-actin

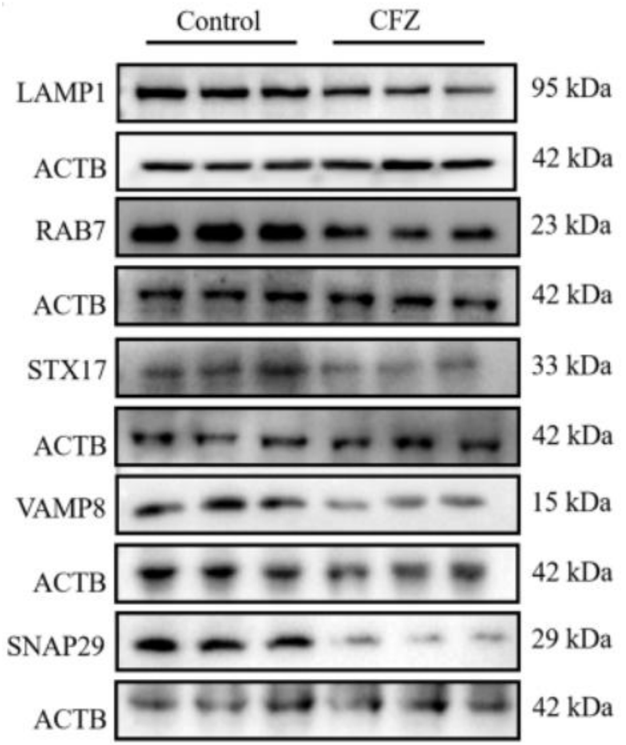
Control
CFZ
LAMP1
95 kDa
ACTB
42 kDa
RAB7
23 kDa
ACTB
42 kDa
STX17
33 kDa
ACTB
42 kDa
VAMP8
15 kDa
ACTB
42 kDa
SNAP29
29 kDa
ACTB
42 kDa

Figure 7

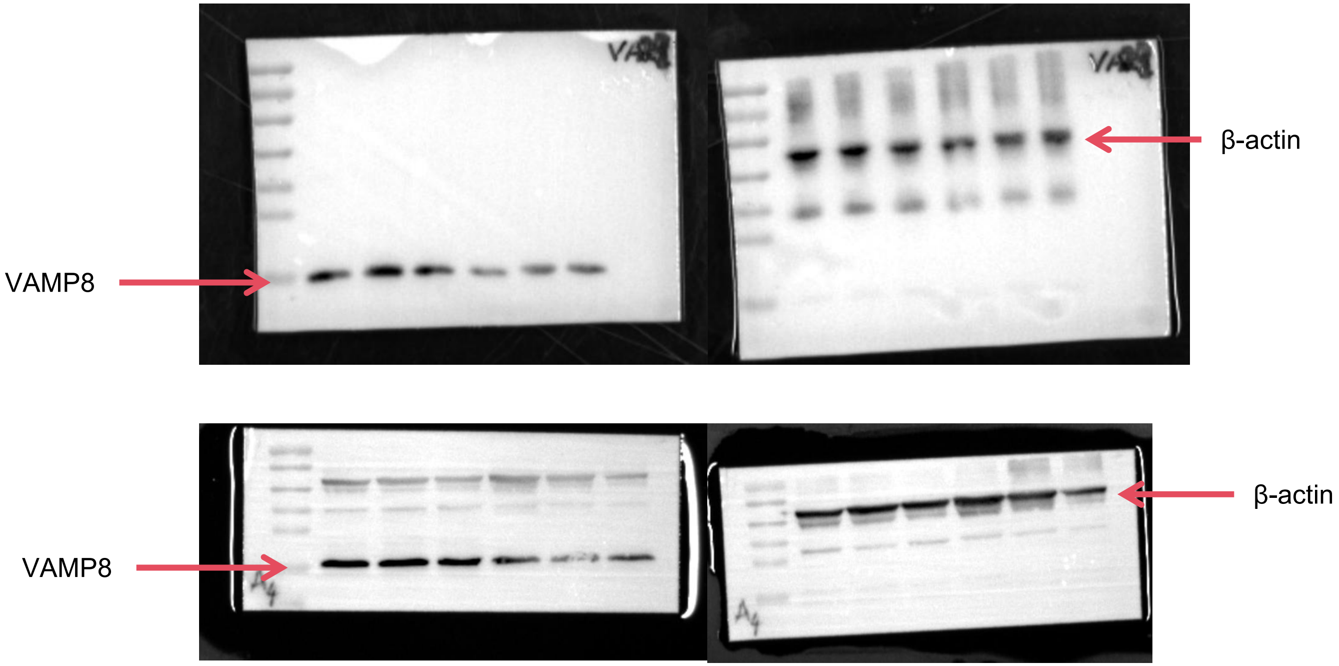

VAMP8
β-actin
VAMP8
β-actin

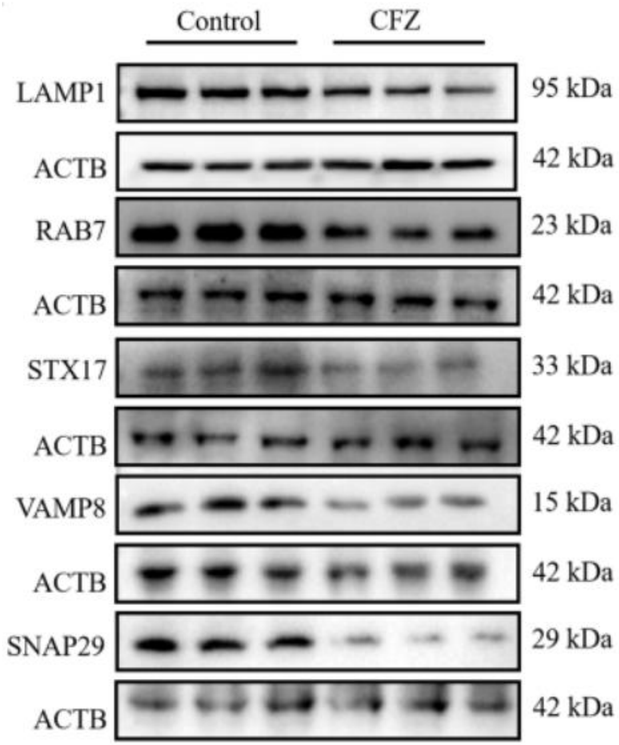

Control
CFZ
LAMP1
95 kDa
ACTB
42 kDa
RAB7
23 kDa
ACTB
42 kDa
STX17
33 kDa
ACTB
42 kDa
VAMP8
15 kDa
ACTB
42 kDa
SNAP29
29 kDa
ACTB
42 kDa

Figure 7

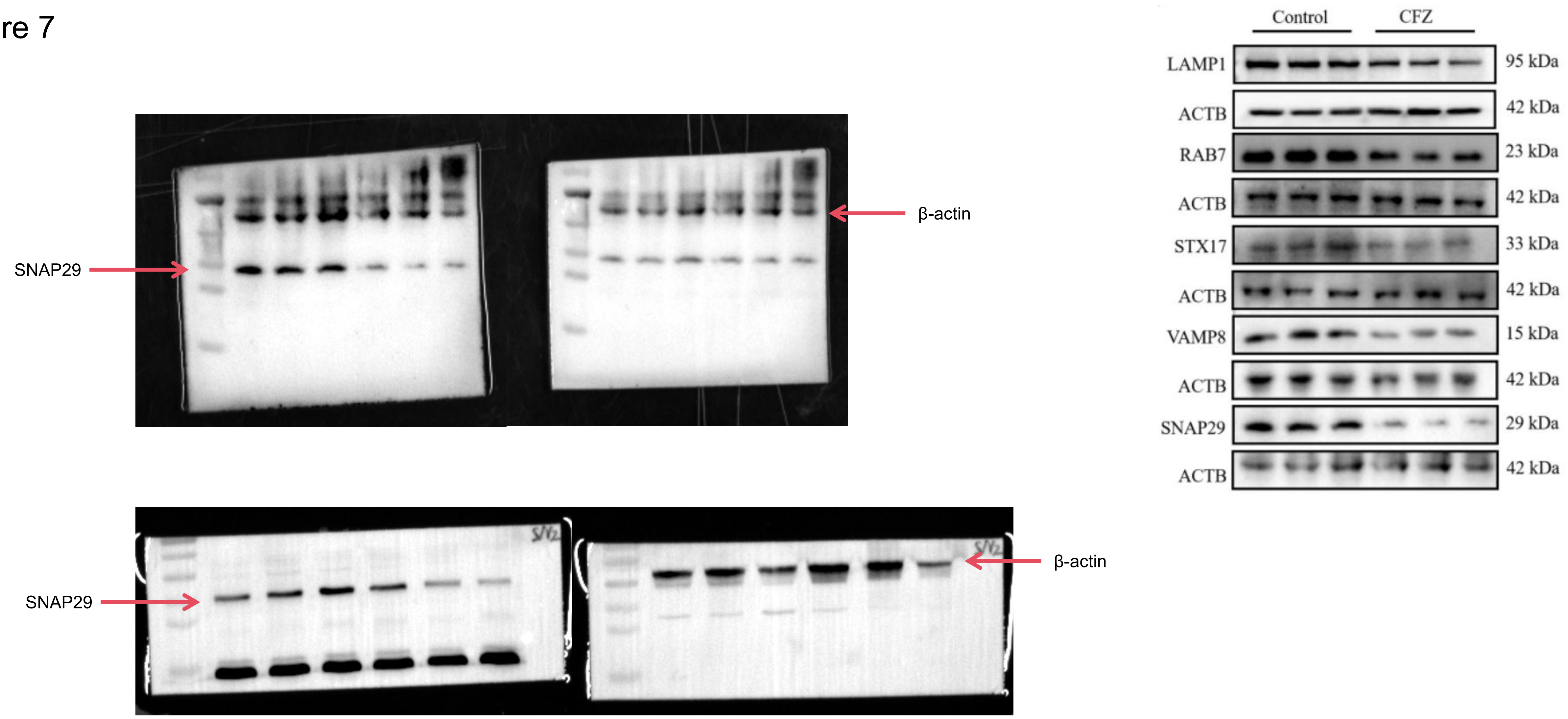

SNAP29
β-actin
SNAP29
β-actin
Control
CFZ
LAMP1
ACTB
RAB7
ACTB
STX17
ACTB
VAMP8
ACTB
SNAP29
ACTB
95 kDa
42 kDa
23 kDa
42 kDa
33 kDa
42 kDa
15 kDa
42 kDa
29 kDa
42 kDa

Figure 7

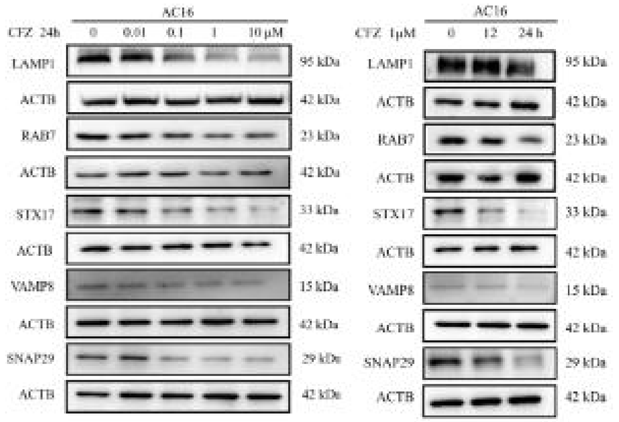

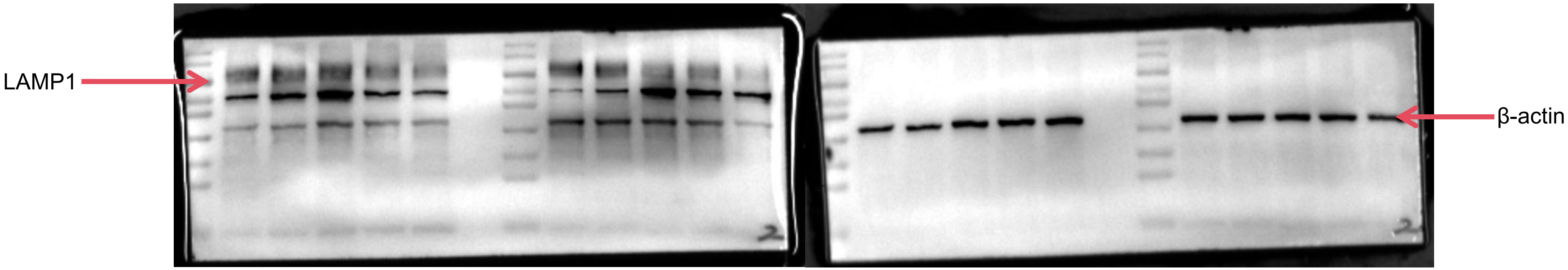

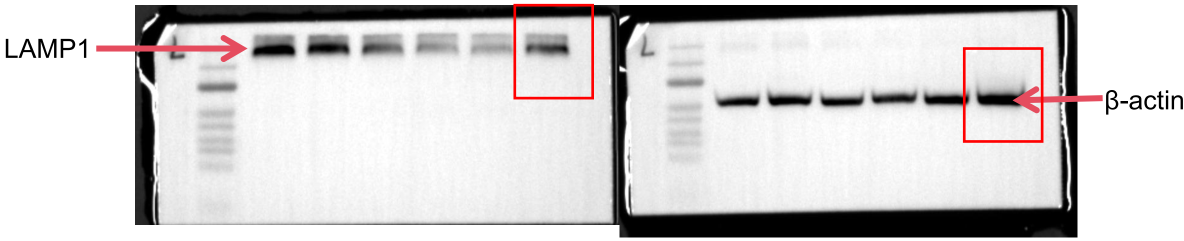

starving as a positive control

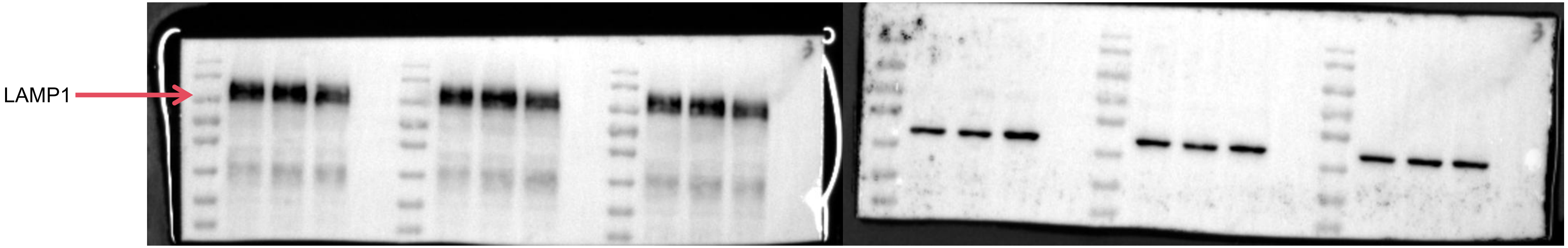

Figure 7

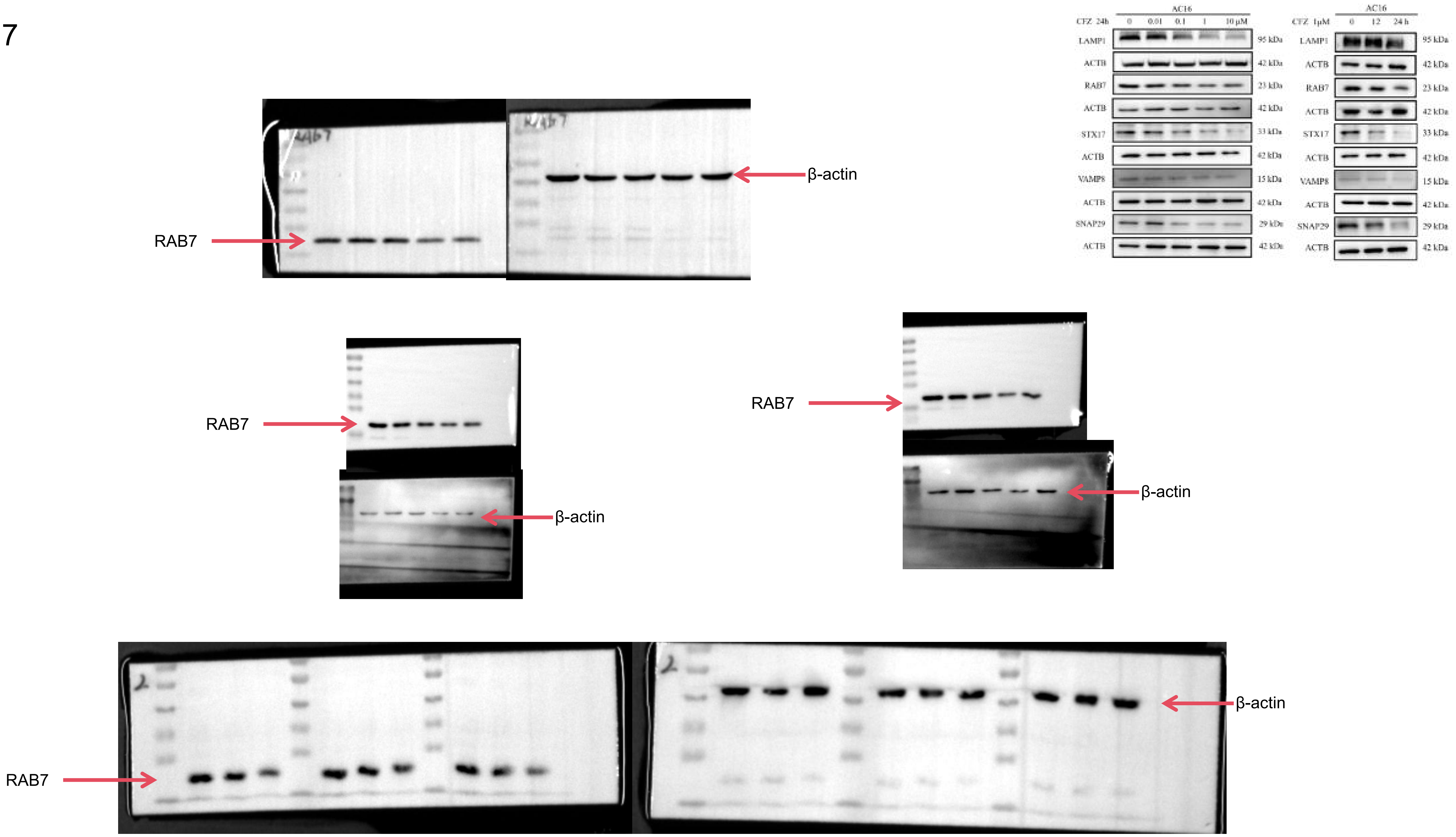
AC16
CFZ 24h
0
0.01
0.1
1
10 μM
LAMP1
95 kDa
ACTB
42 kDa
RAB7
23 kDa
STX17
33 kDa
VAMP8
15 kDa
SNAP29
29 kDa
CFZ 1μM
12
24 h
RAB7
β-actin

Figure 7

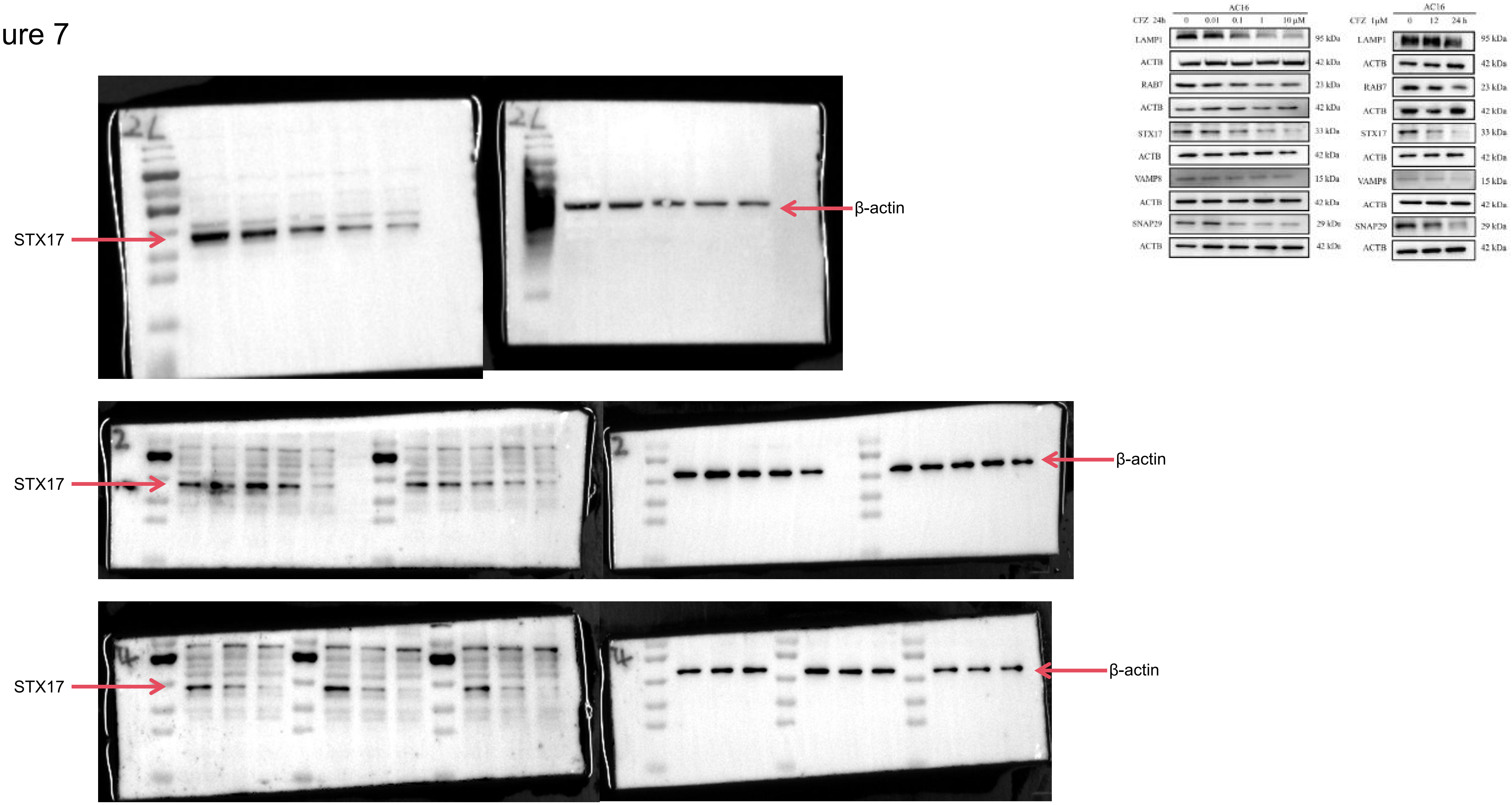

STX17
β-actin
STX17
β-actin
STX17
β-actin
AC16
CFZ 24h
0
0.01
0.1
1
10 μM
LAMP1
ACTB
RAB7
STX17
VAMP8
SNAP29
95 kDa
42 kDa
23 kDa
33 kDa
15 kDa
29 kDa
CFZ 1μM
0
12
24 h

Figure 7

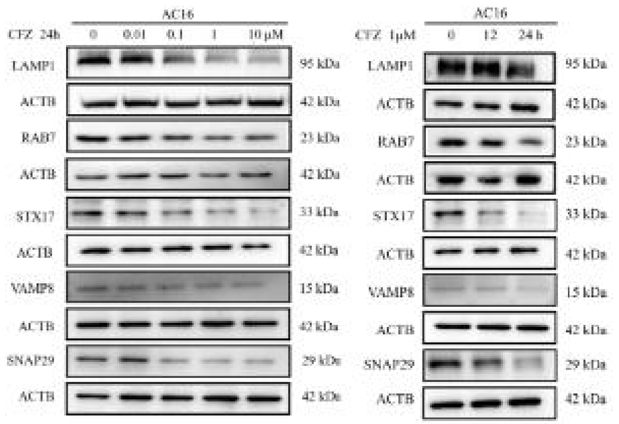
AC16
CFZ 24h
0
0.01
0.1
1
10 μM
LAMP1
ACTB
RAB7
STX17
VAMP8
SNAP29
95 kDa
42 kDa
23 kDa
33 kDa
15 kDa
29 kDa
AC16
CFZ 1μM
0
12
24 h

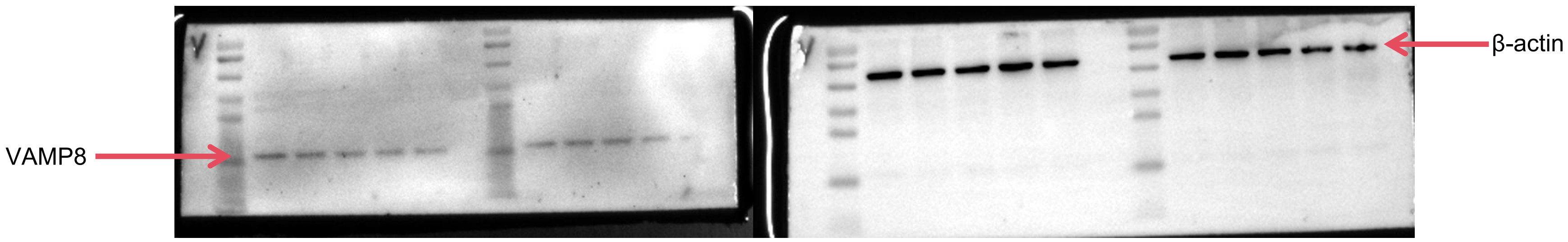
VAMP8
β-actin

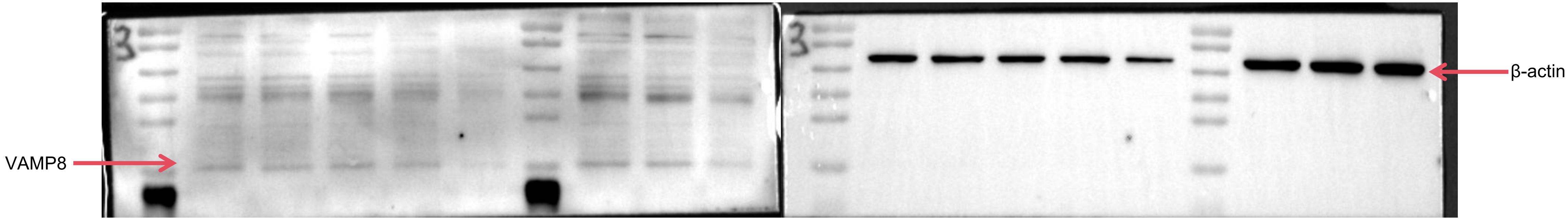
VAMP8
β-actin

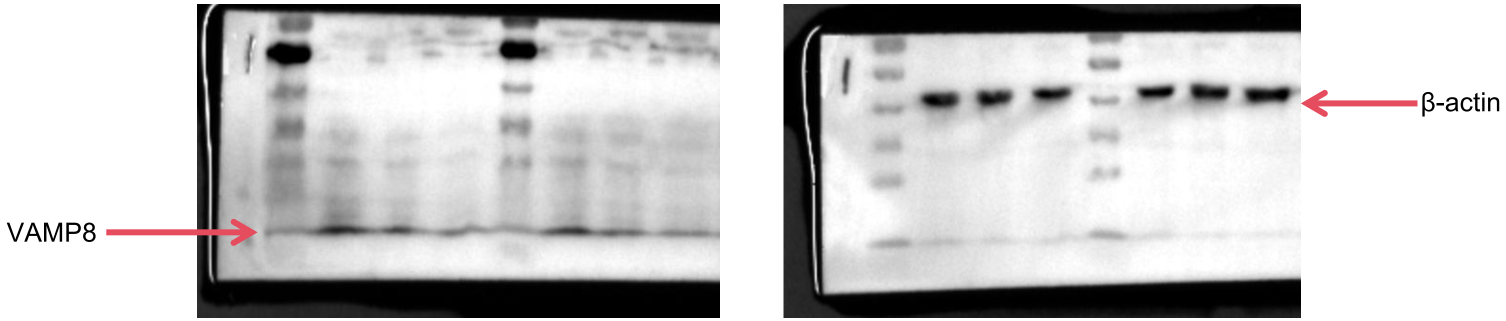
VAMP8
β-actin

Figure 7

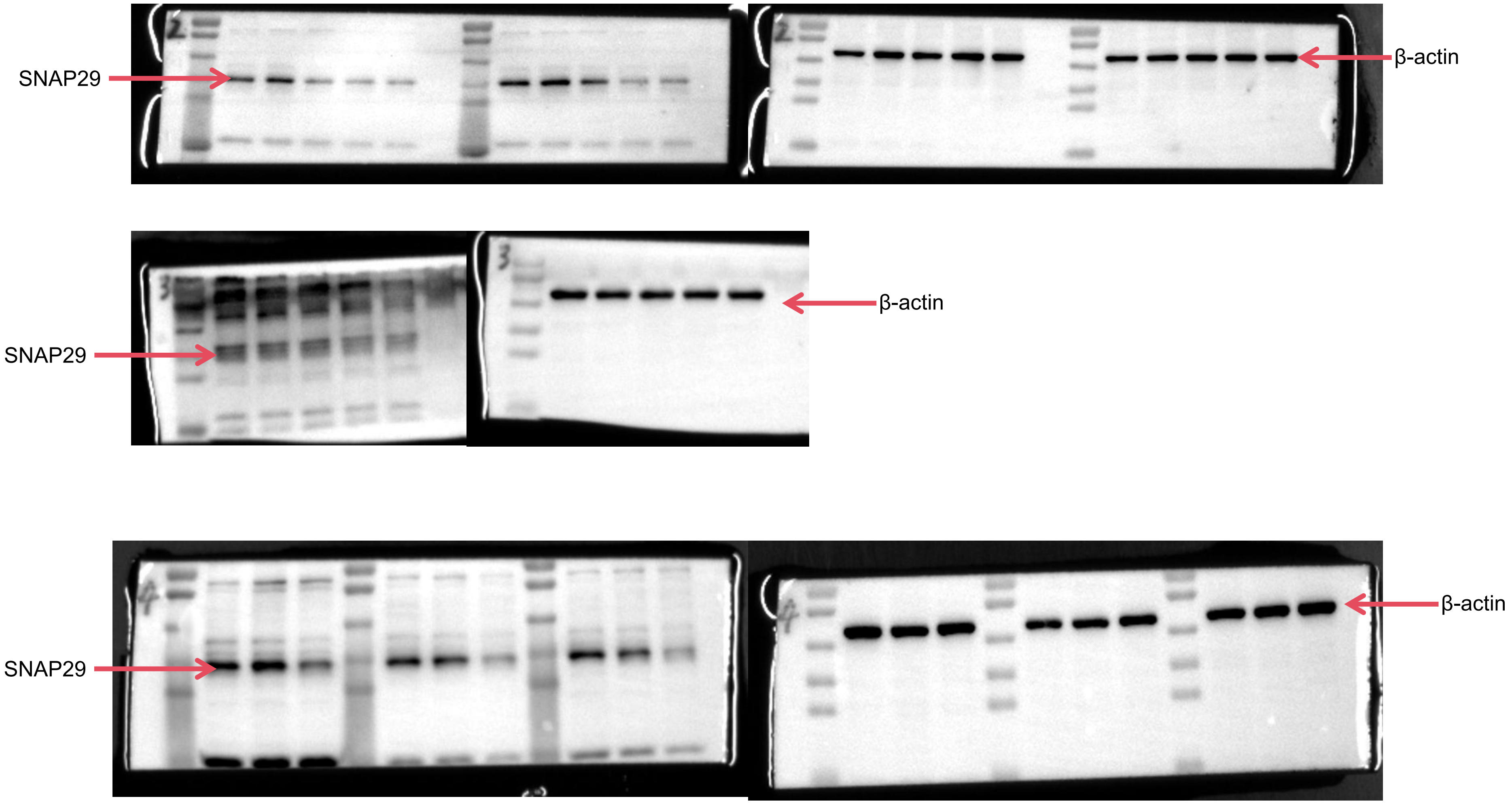
SNAP29
β-actin
SNAP29
β-actin
SNAP29
β-actin

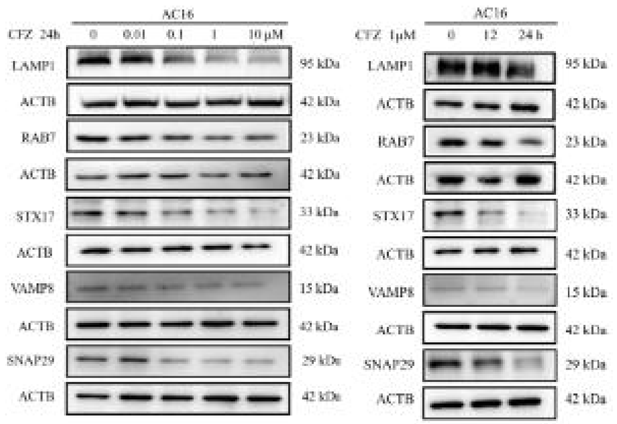
AC16
CFZ 24h
0
0.01
0.1
1
10 μM
CFZ 1μM
0
12
24 h
LAMP1
ACTB
RAB7
STX17
VAMP8
SNAP29

Figure 8

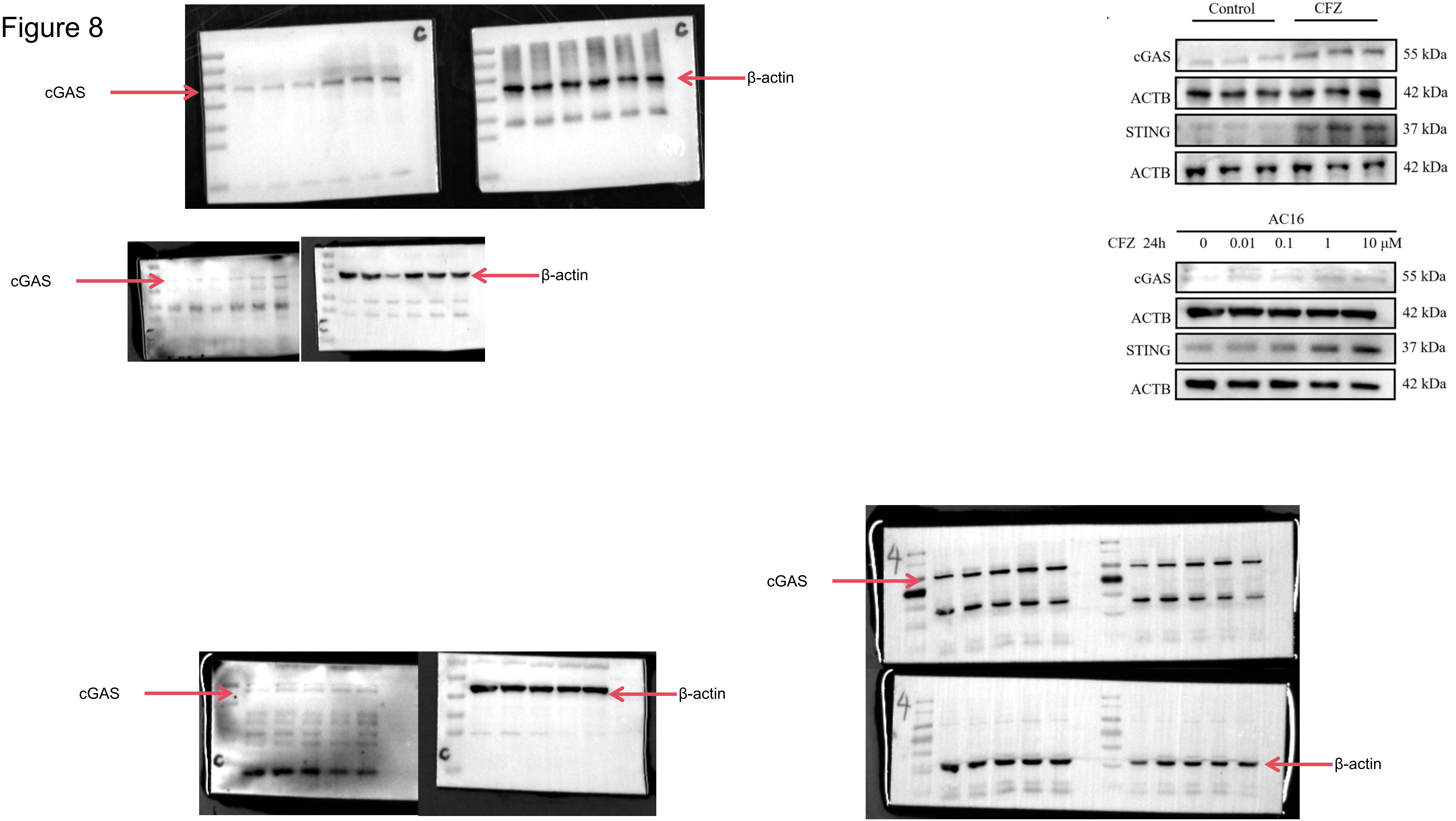
cGAS
β-actin
cGAS
β-actin
Control
CFZ
cGAS
55 kDa
ACTB
42 kDa
STING
37 kDa
ACTB
42 kDa
AC16
CFZ 24h
0
0.01
0.1
1
10 μM
cGAS
55 kDa
ACTB
42 kDa
STING
37 kDa
ACTB
42 kDa
cGAS
β-actin
cGAS
β-actin

Figure 8

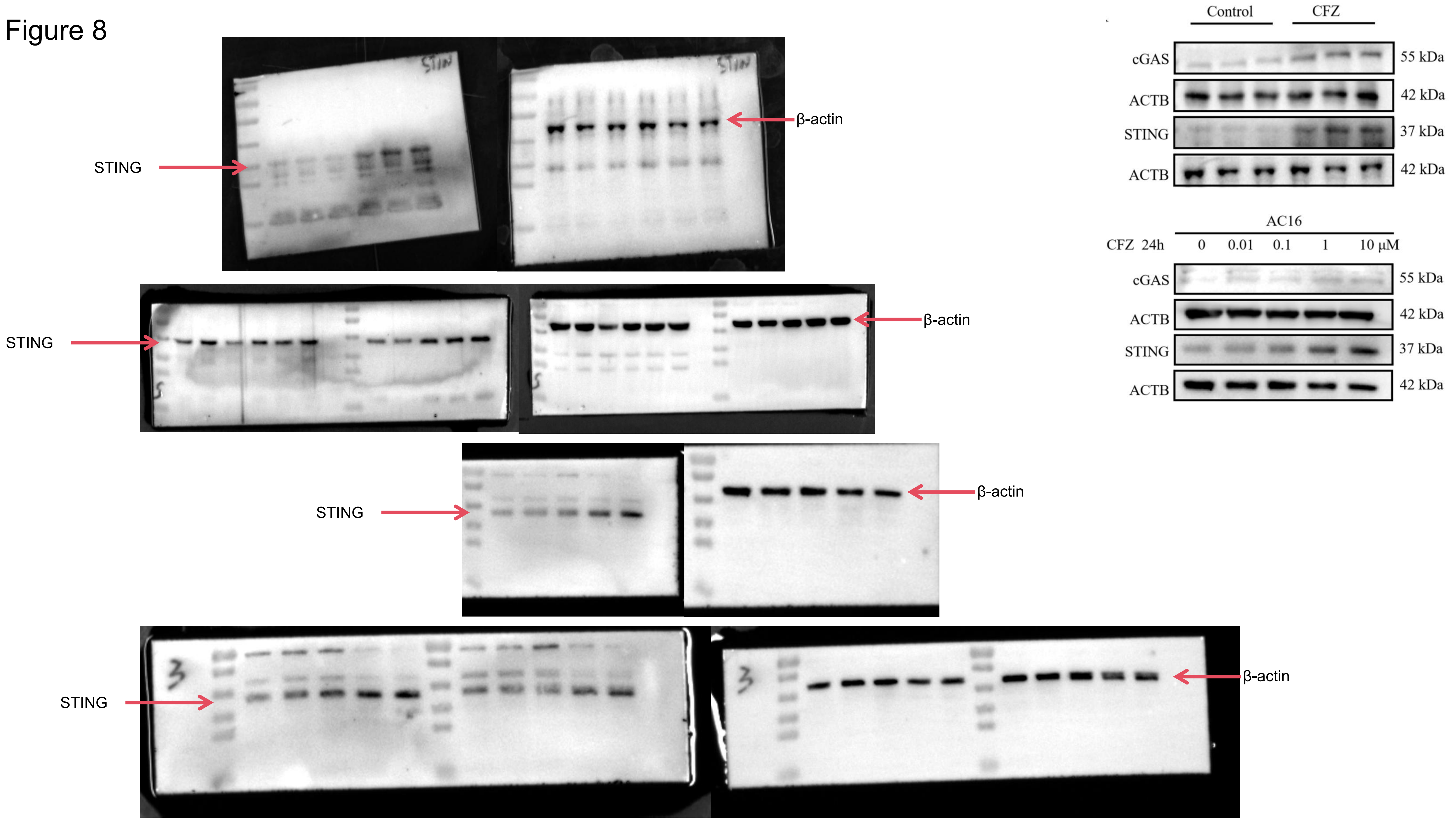
STING
β-actin
STING
β-actin
STING
β-actin
STING
β-actin
Control
CFZ
cGAS
55 kDa
ACTB
42 kDa
STING
37 kDa
ACTB
42 kDa
AC16
CFZ 24h
0
0.01
0.1
1
10 μM
cGAS
55 kDa
ACTB
42 kDa
STING
37 kDa
ACTB
42 kDa

Figure 8

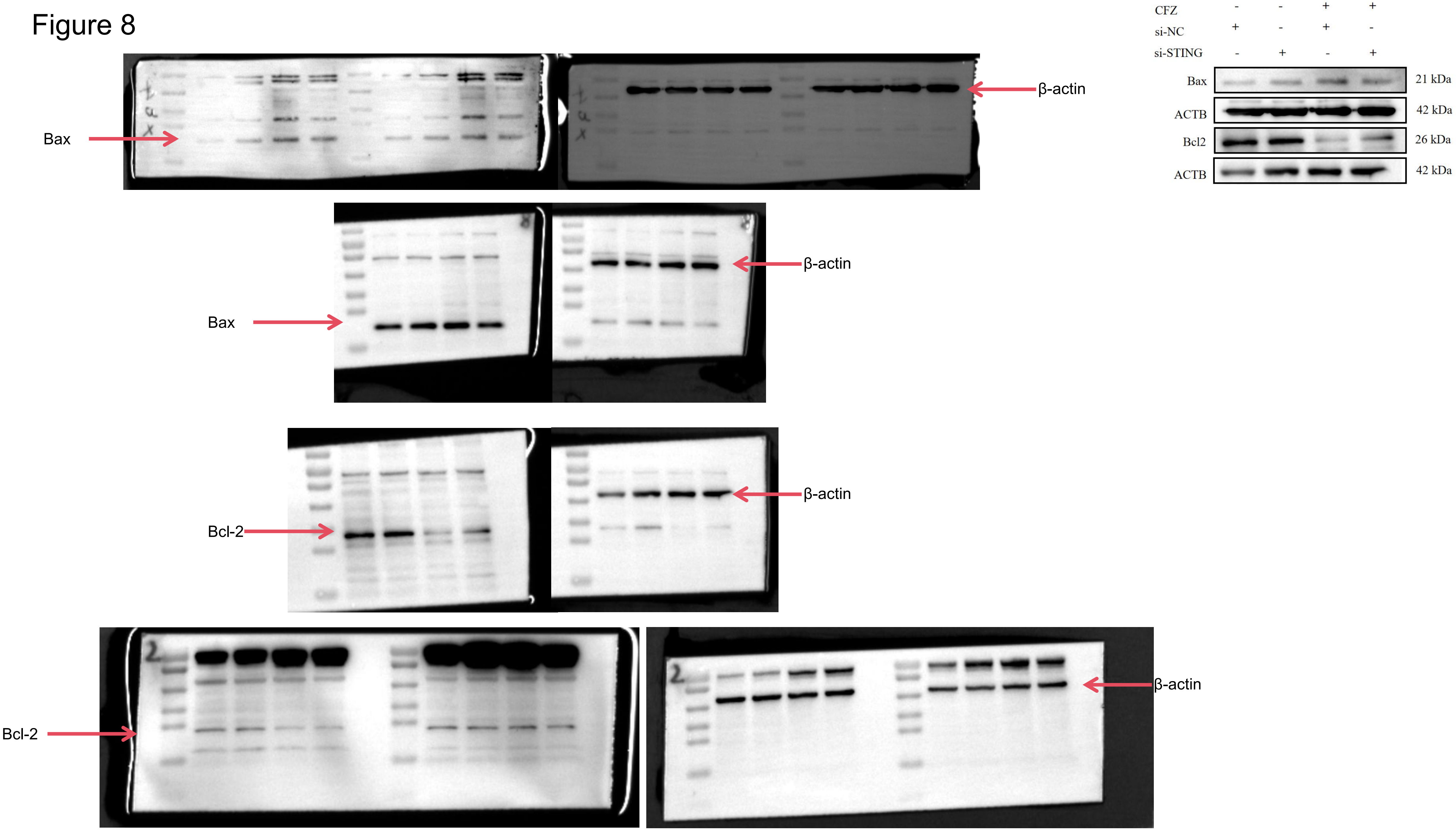

Bax
β-actin
Bax
β-actin
Bcl-2
β-actin
Bcl-2
β-actin
CFZ - - + +
si-NC + - + -
si-STING - + - +
Bax
21 kDa
ACTB
42 kDa
Bcl2
26 kDa
ACTB
42 kDa

Figure 8

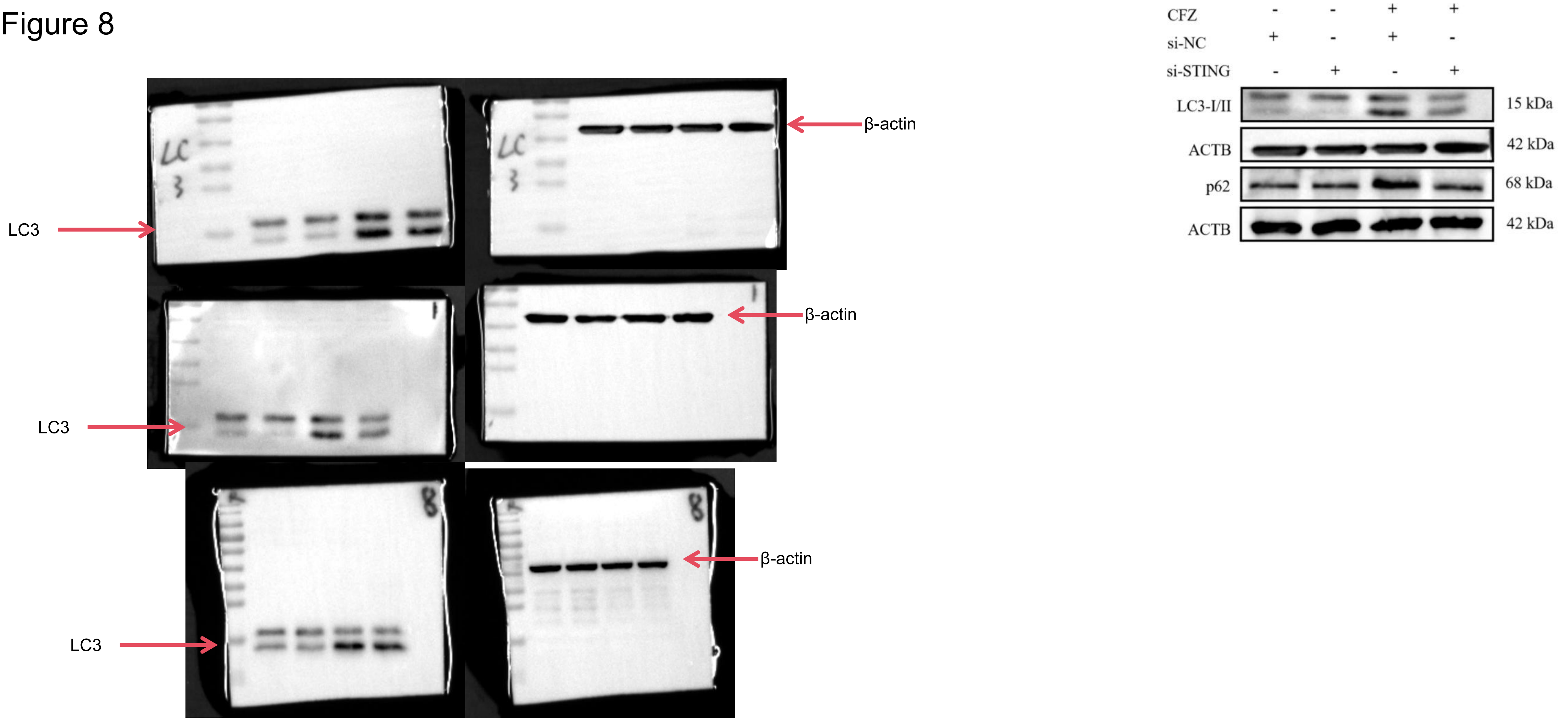
CFZ - - + +
si-NC + - + -
si-STING - + - +
LC3-I/II
15 kDa
ACTB
42 kDa
p62
68 kDa
ACTB
42 kDa
LC3
β-actin
LC3
β-actin
LC3
β-actin

Figure 8

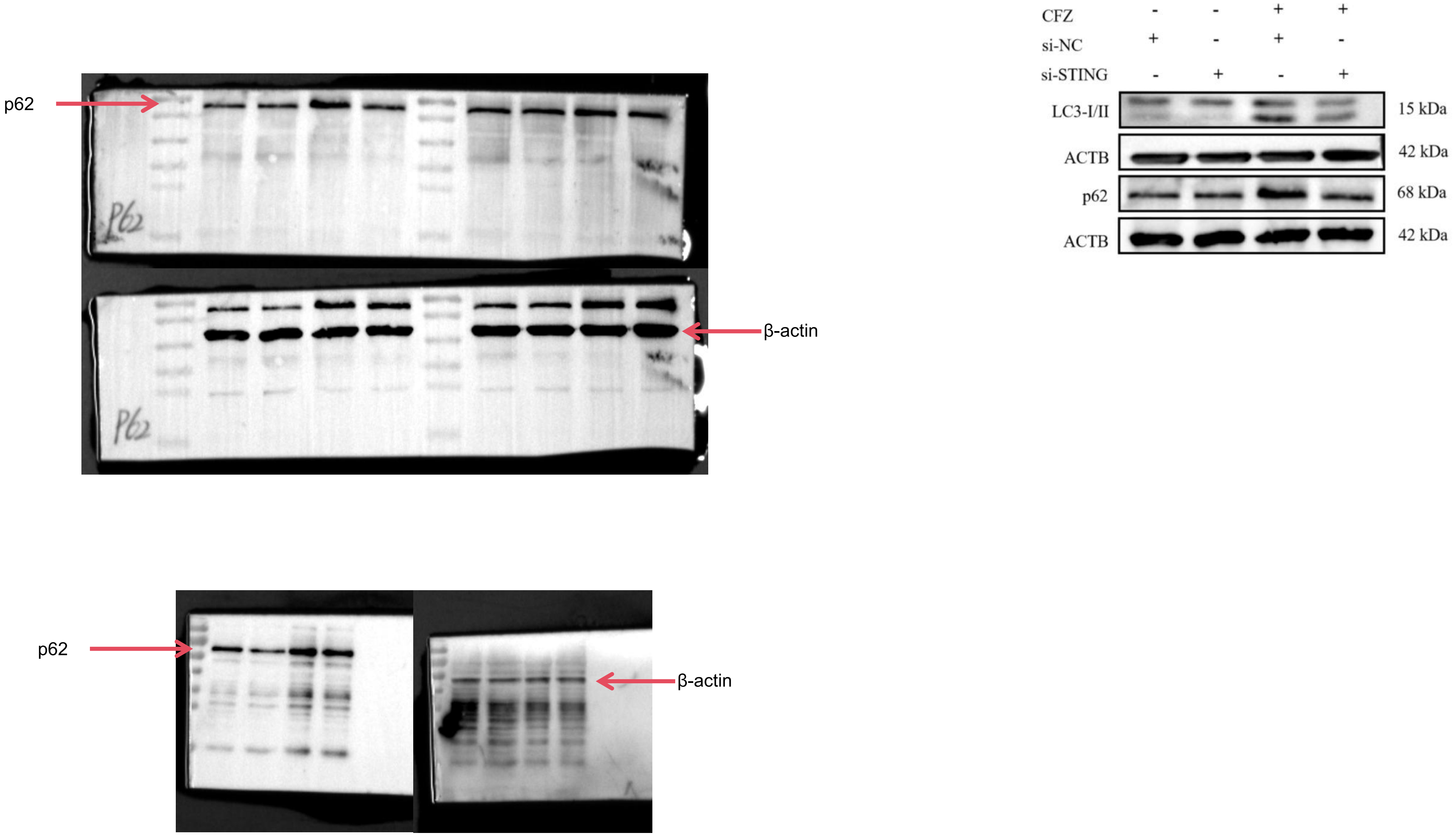
p62
β-actin
p62
β-actin
CFZ
si-NC
si-STING
LC3-I/II
ACTB
p62
ACTB
15 kDa
42 kDa
68 kDa
42 kDa

Figure 8

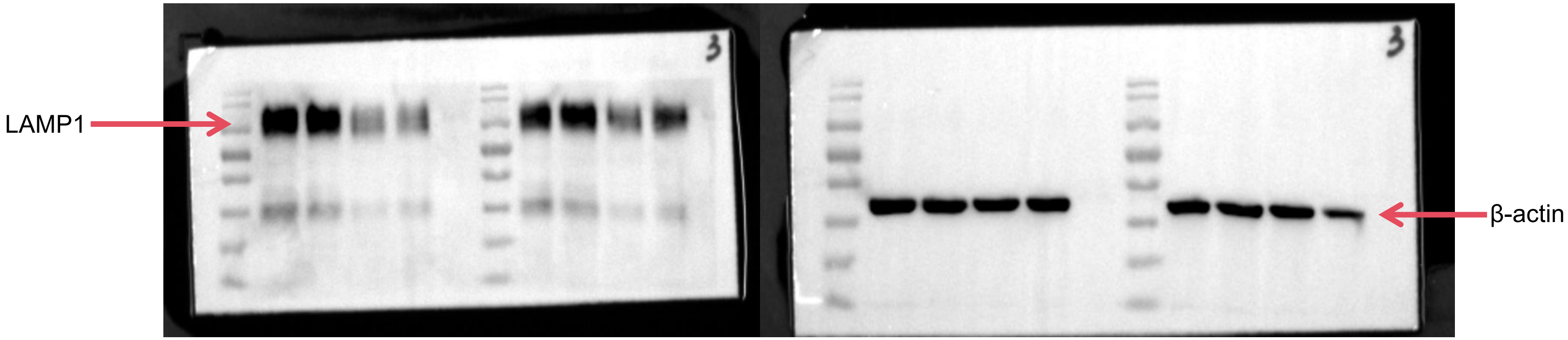
LAMP1
β-actin

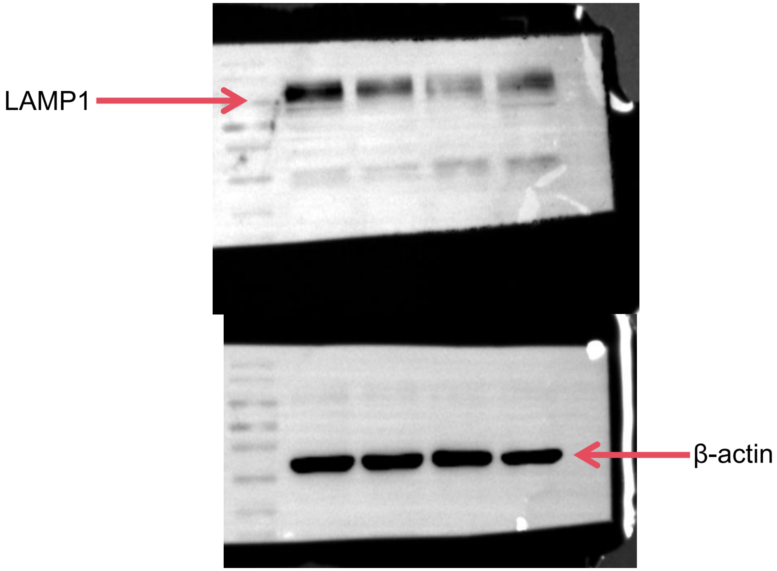
LAMP1
β-actin

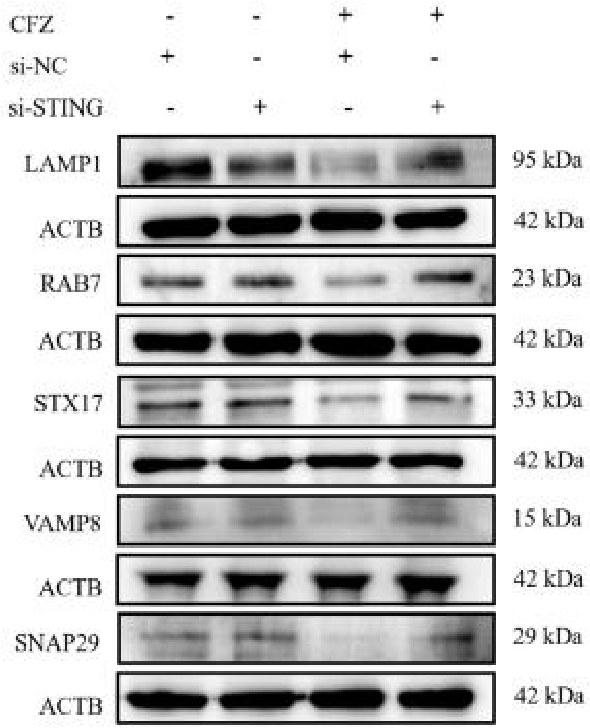
CFZ - - + +
si-NC + - + -
si-STING - + - +
LAMP1 95 kDa
ACTB 42 kDa
RAB7 23 kDa
ACTB 42 kDa
STX17 33 kDa
ACTB 42 kDa
VAMP8 15 kDa
ACTB 42 kDa
SNAP29 29 kDa
ACTB 42 kDa

Figure 8

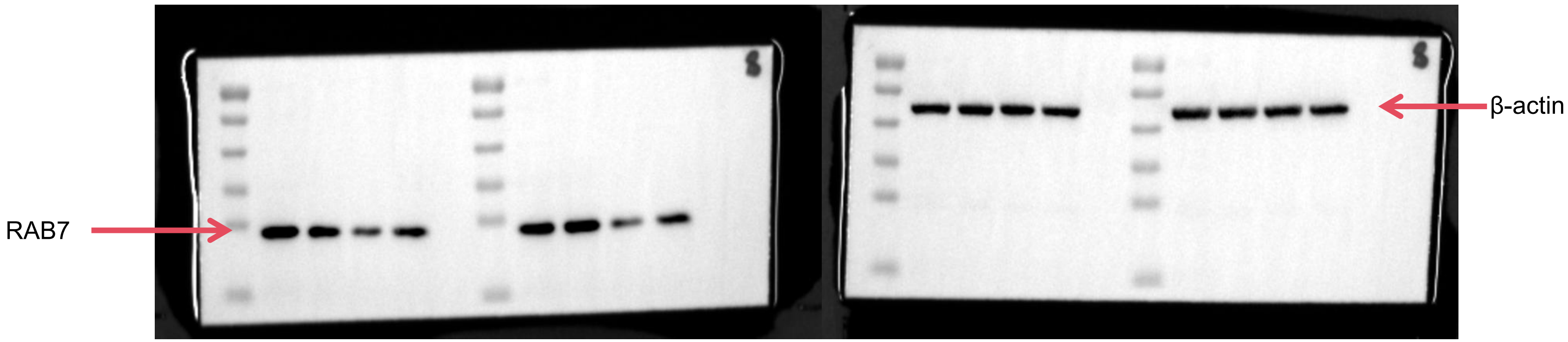
RAB7
β-actin

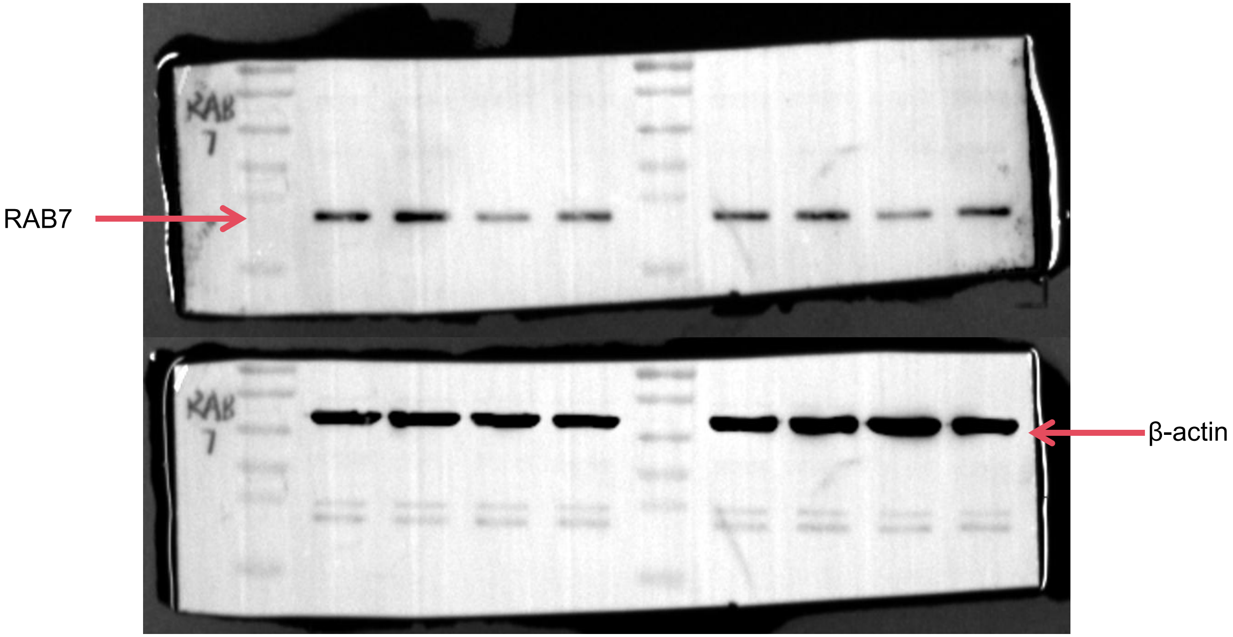
RAB7
β-actin

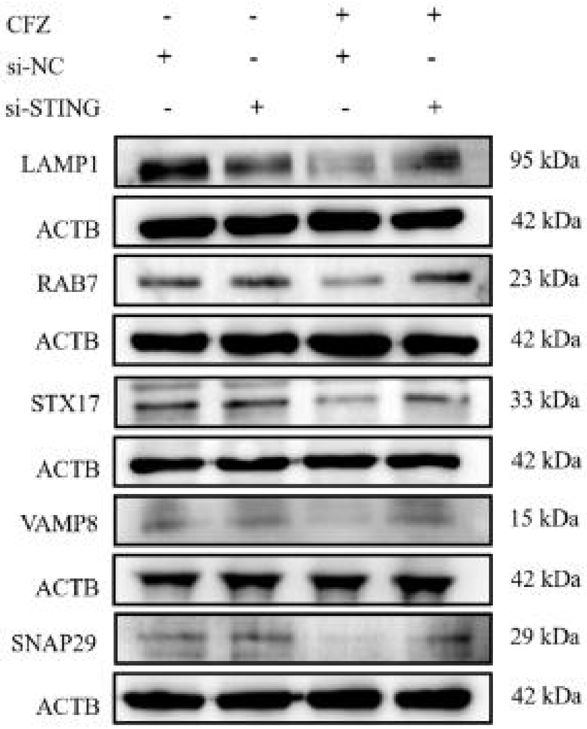
CFZ - - + +
si-NC + - + -
si-STING - + - +
LAMP1
95 kDa
ACTB
42 kDa
RAB7
23 kDa
ACTB
42 kDa
STX17
33 kDa
ACTB
42 kDa
VAMP8
15 kDa
ACTB
42 kDa
SNAP29
29 kDa
ACTB
42 kDa

Figure 8

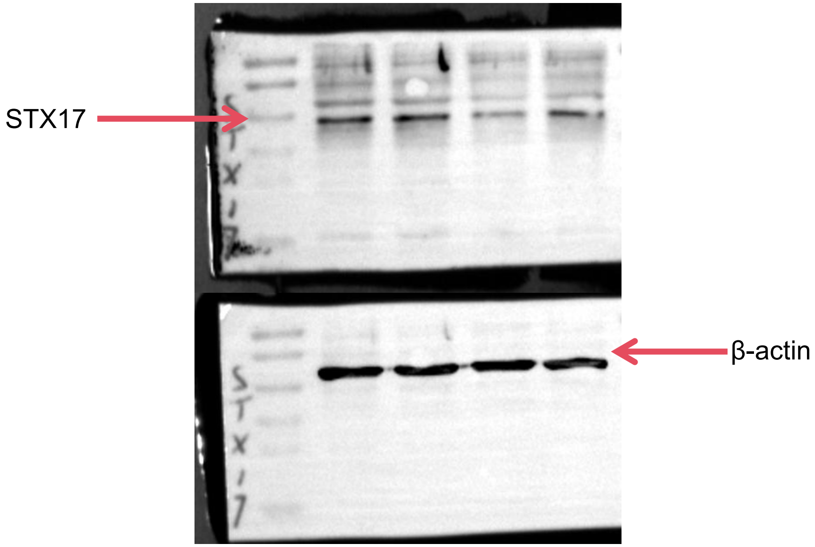

STX17
β-actin

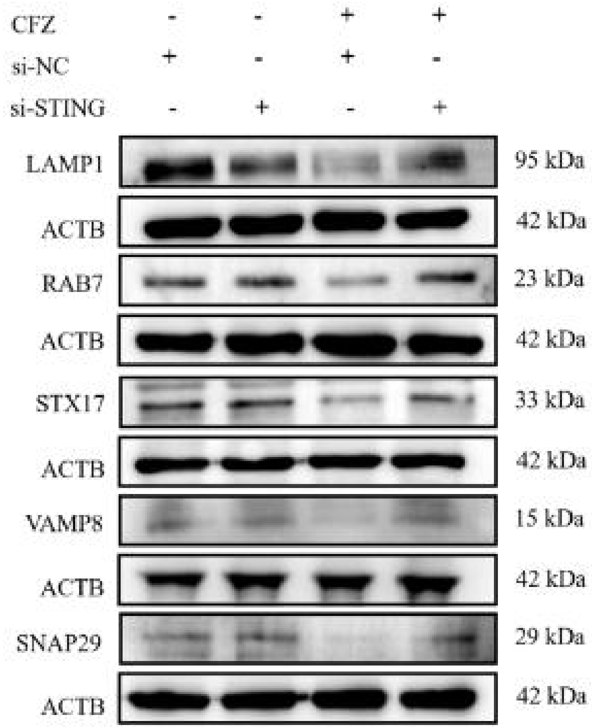

CFZ - - + +
si-NC + - + -
si-STING - + - +
LAMP1
95 kDa
ACTB
42 kDa
RAB7
23 kDa
ACTB
42 kDa
STX17
33 kDa
ACTB
42 kDa
VAMP8
15 kDa
ACTB
42 kDa
SNAP29
29 kDa
ACTB
42 kDa

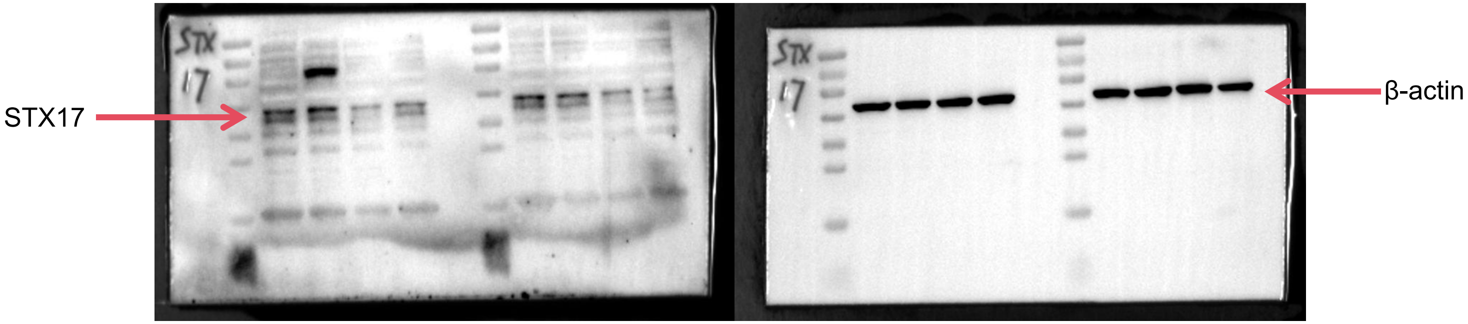

STX17
β-actin

Figure 8

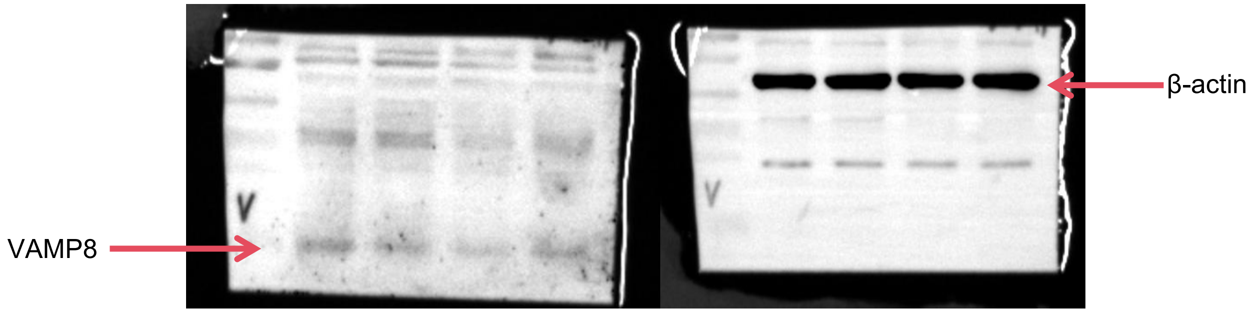
VAMP8
β-actin

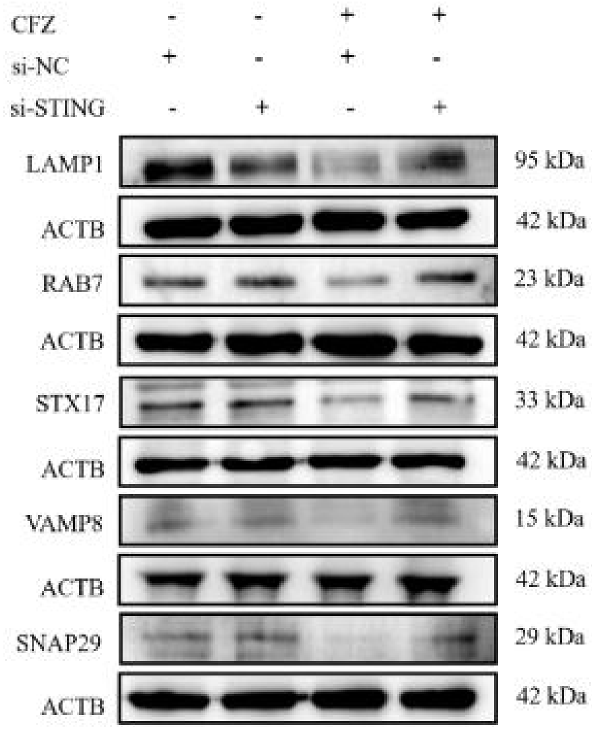
CFZ - - + +
si-NC + - + -
si-STING - + - +
LAMP1
95 kDa
ACTB
42 kDa
RAB7
23 kDa
ACTB
42 kDa
STX17
33 kDa
ACTB
42 kDa
VAMP8
15 kDa
ACTB
42 kDa
SNAP29
29 kDa
ACTB
42 kDa

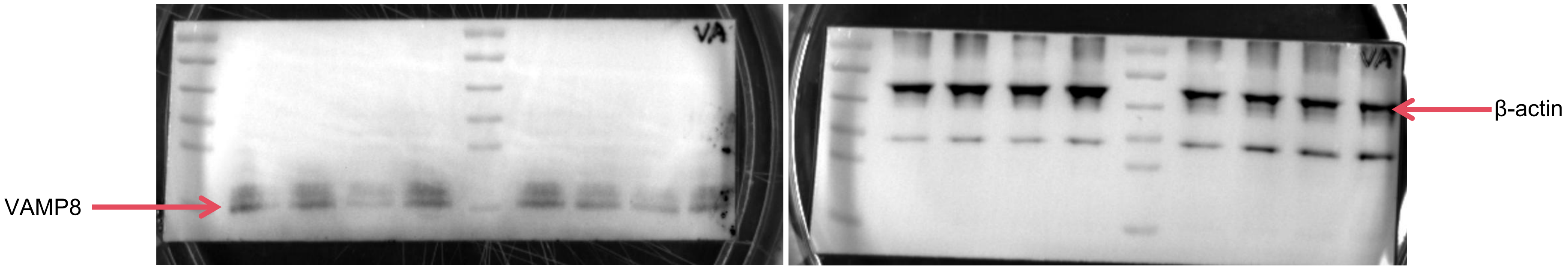
VAMP8
β-actin

Figure 8

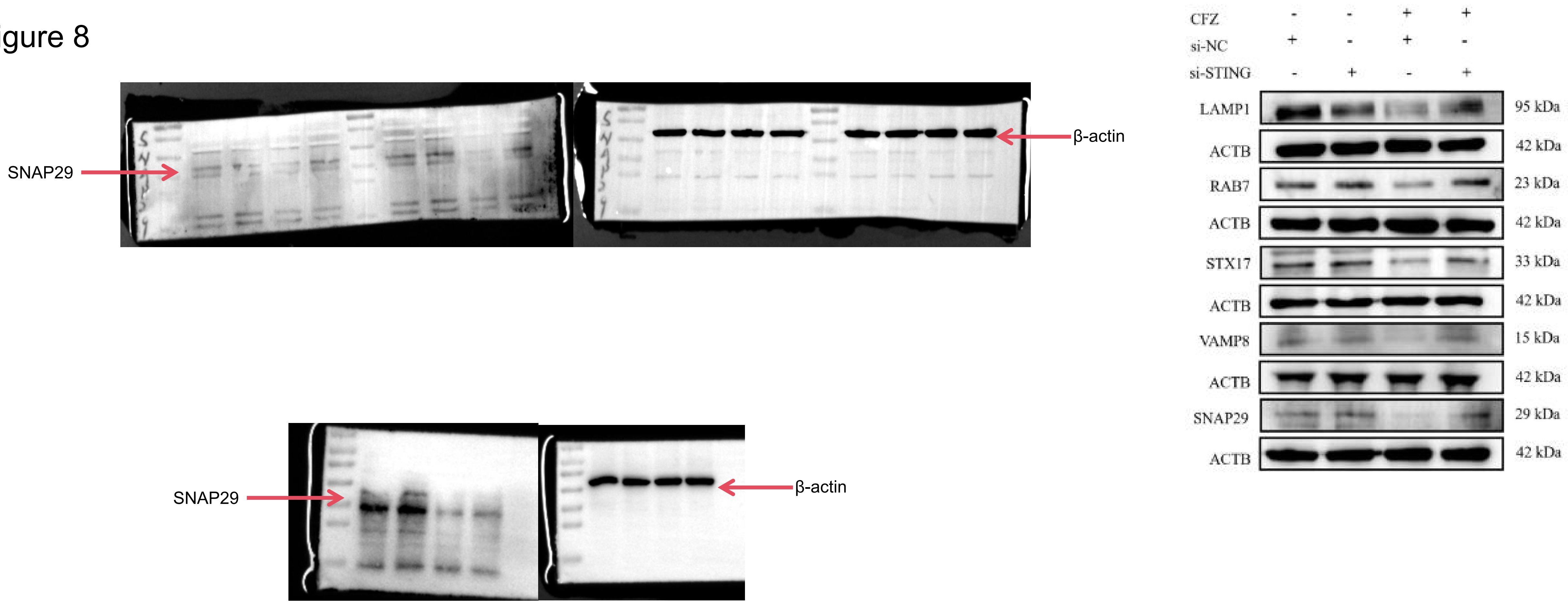
SNAP29
β-actin
SNAP29
β-actin
CFZ - - + +
si-NC + - + -
si-STING - + - +
LAMP1 95 kDa
ACTB 42 kDa
RAB7 23 kDa
ACTB 42 kDa
STX17 33 kDa
ACTB 42 kDa
VAMP8 15 kDa
ACTB 42 kDa
SNAP29 29 kDa
ACTB 42 kDa

Figure 9

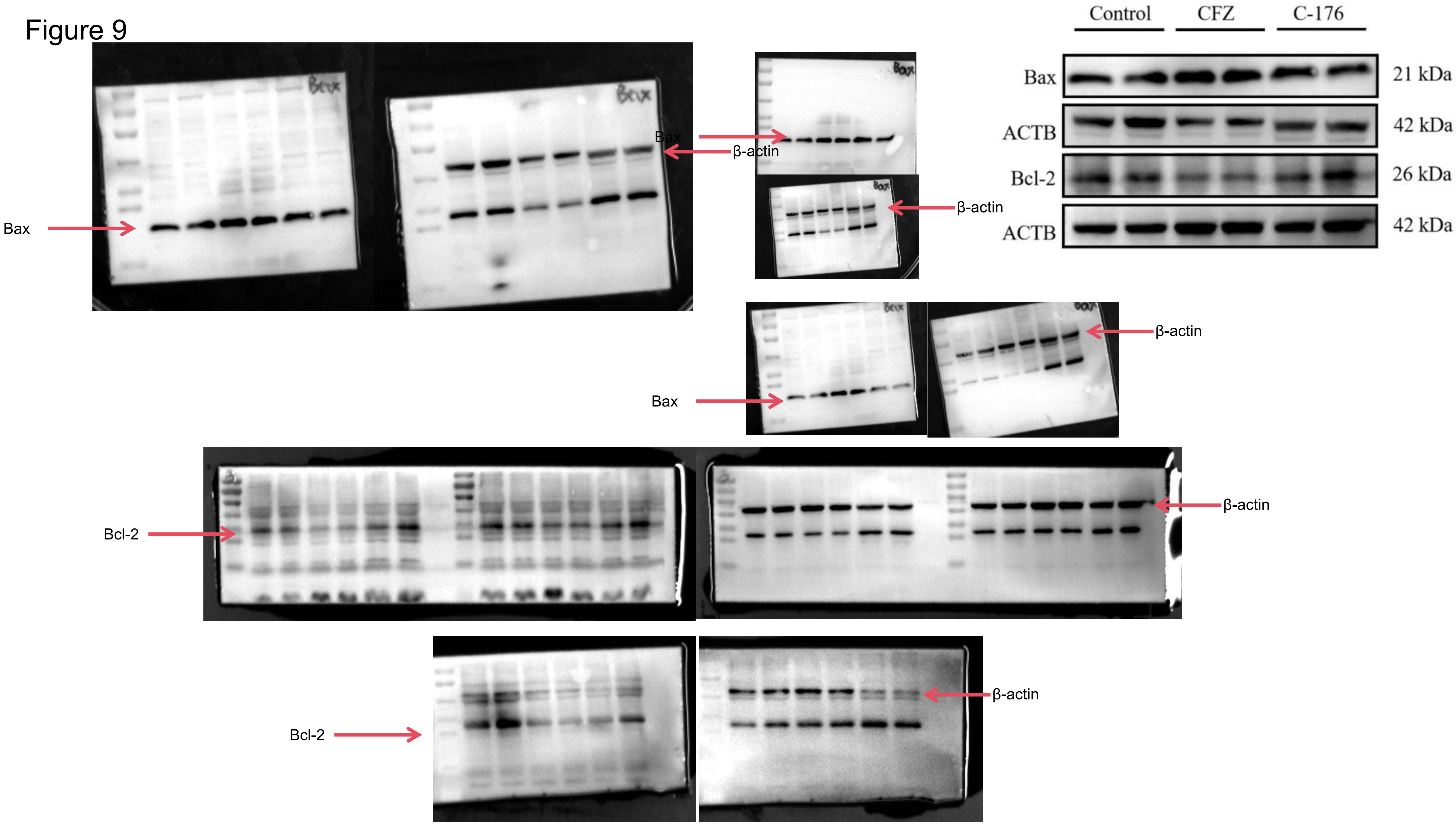
Control
CFZ
C-176
Bax
21 kDa
ACTB
42 kDa
Bcl-2
26 kDa
ACTB
42 kDa
Bax
Bax
β-actin
β-actin
β-actin
Bax
Bcl-2
β-actin
Bcl-2
β-actin

Figure 9

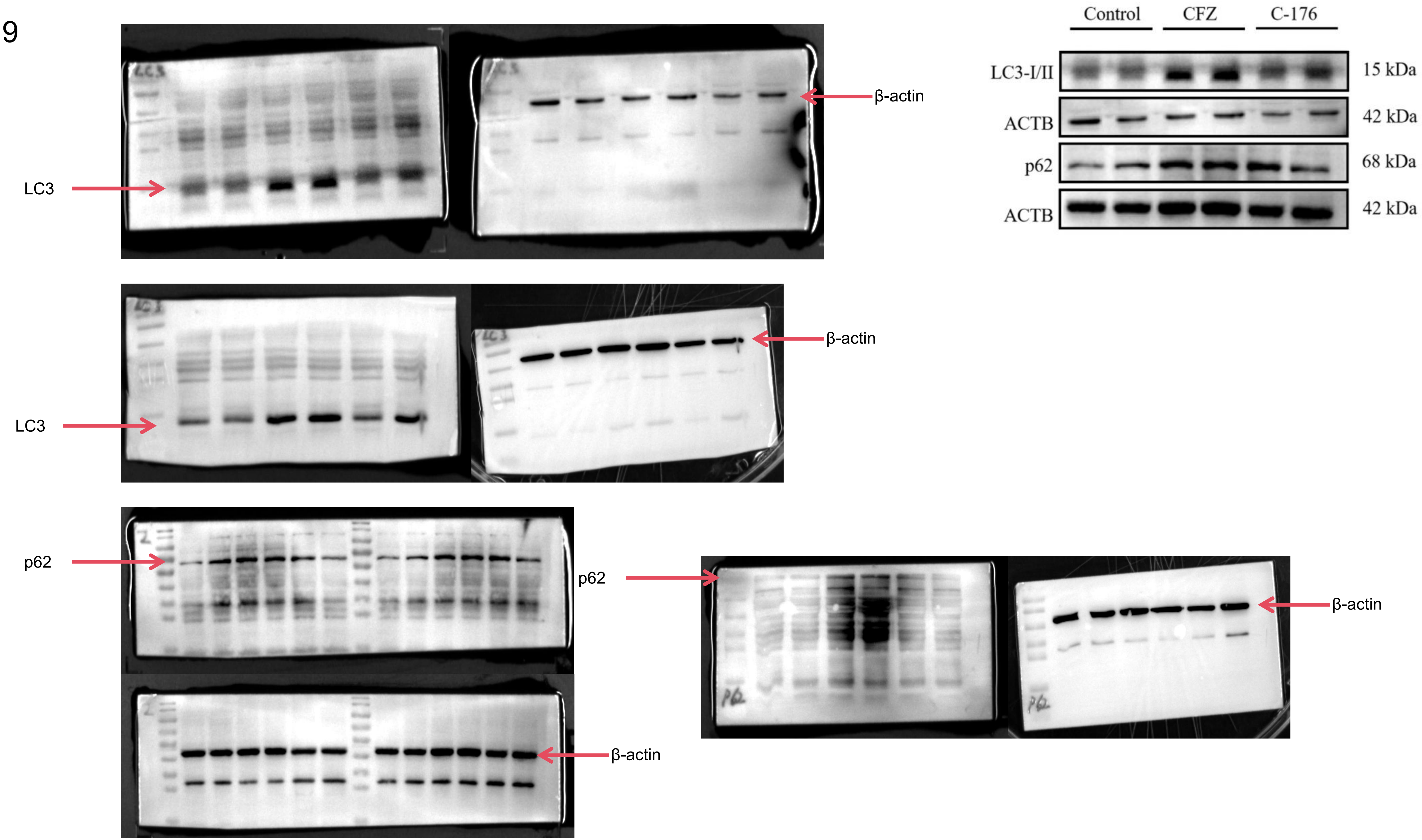

Control
CFZ
C-176
LC3-I/II
15 kDa
ACTB
42 kDa
p62
68 kDa
ACTB
42 kDa
LC3
β-actin
LC3
β-actin
p62
p62
β-actin
β-actin

Figure 9

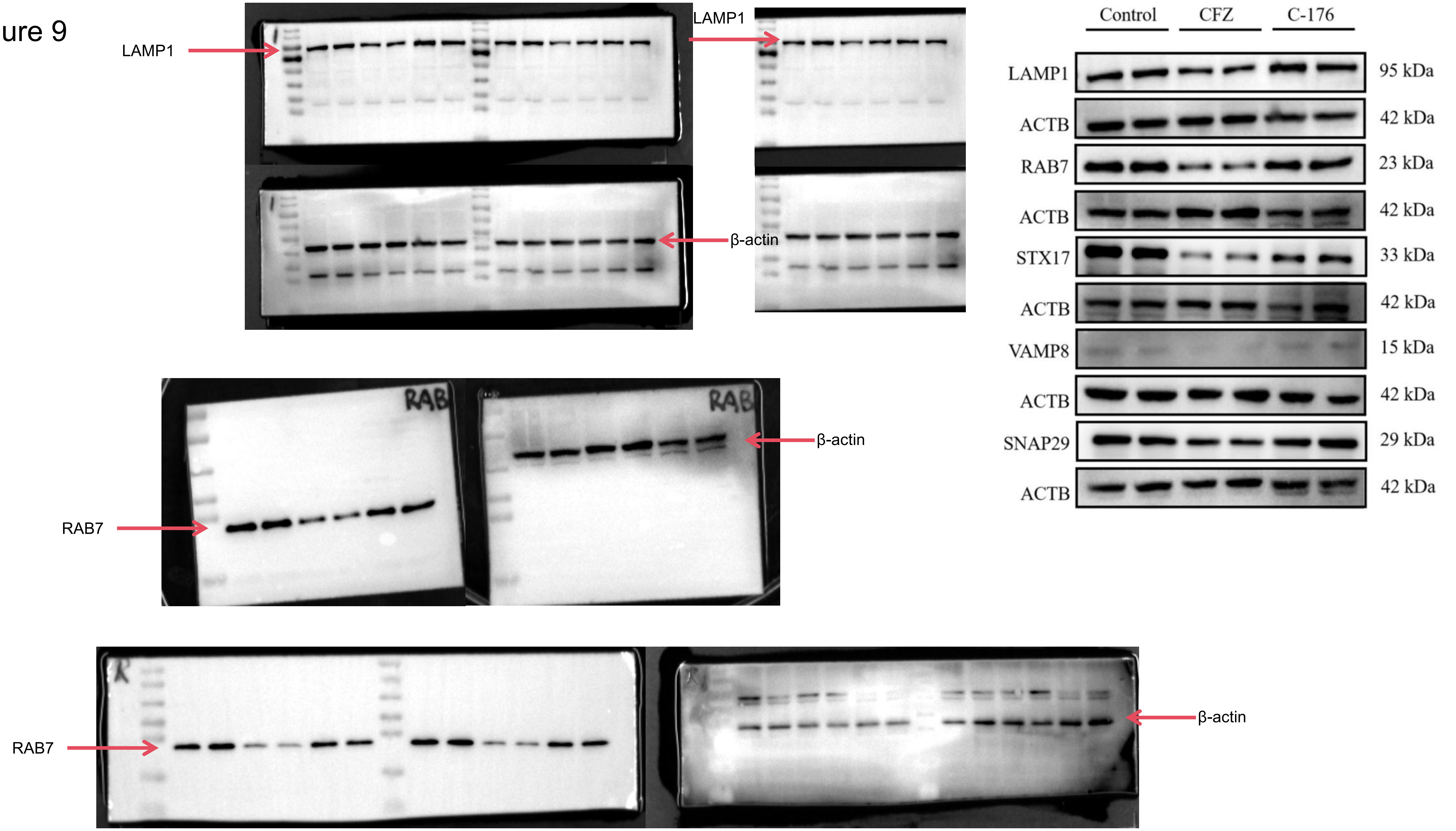
LAMP1
LAMP1
β-actin
RAB7
RAB
RAB
β-actin
RAB7
β-actin
Control
CFZ
C-176
LAMP1
95 kDa
ACTB
42 kDa
RAB7
23 kDa
ACTB
42 kDa
STX17
33 kDa
ACTB
42 kDa
VAMP8
15 kDa
ACTB
42 kDa
SNAP29
29 kDa
ACTB
42 kDa

Figure 9

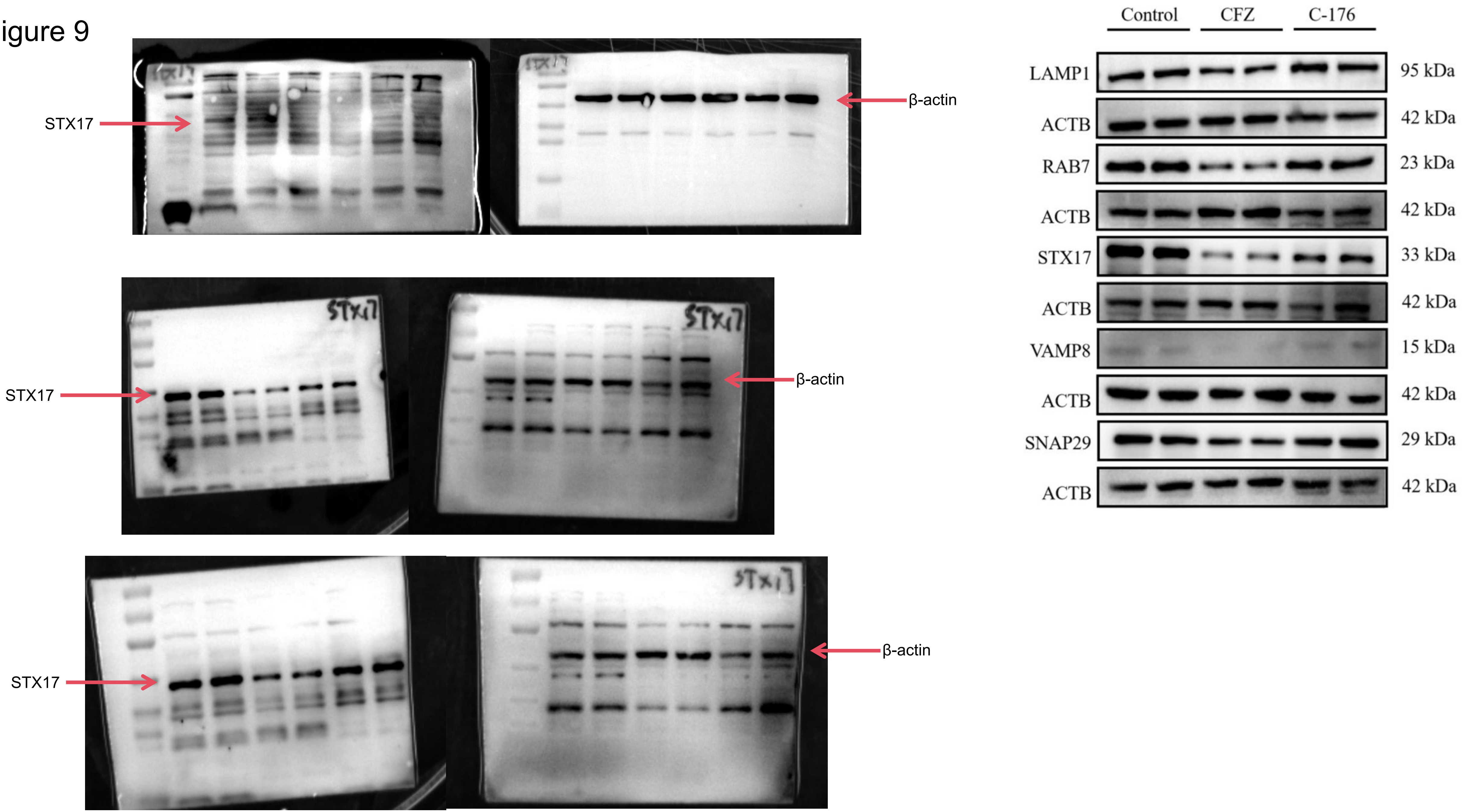
STX17
β-actin
STX17
β-actin
STX17
β-actin
Control
CFZ
C-176
LAMP1
95 kDa
ACTB
42 kDa
RAB7
23 kDa
ACTB
42 kDa
STX17
33 kDa
ACTB
42 kDa
VAMP8
15 kDa
ACTB
42 kDa
SNAP29
29 kDa
ACTB
42 kDa

Figure 9

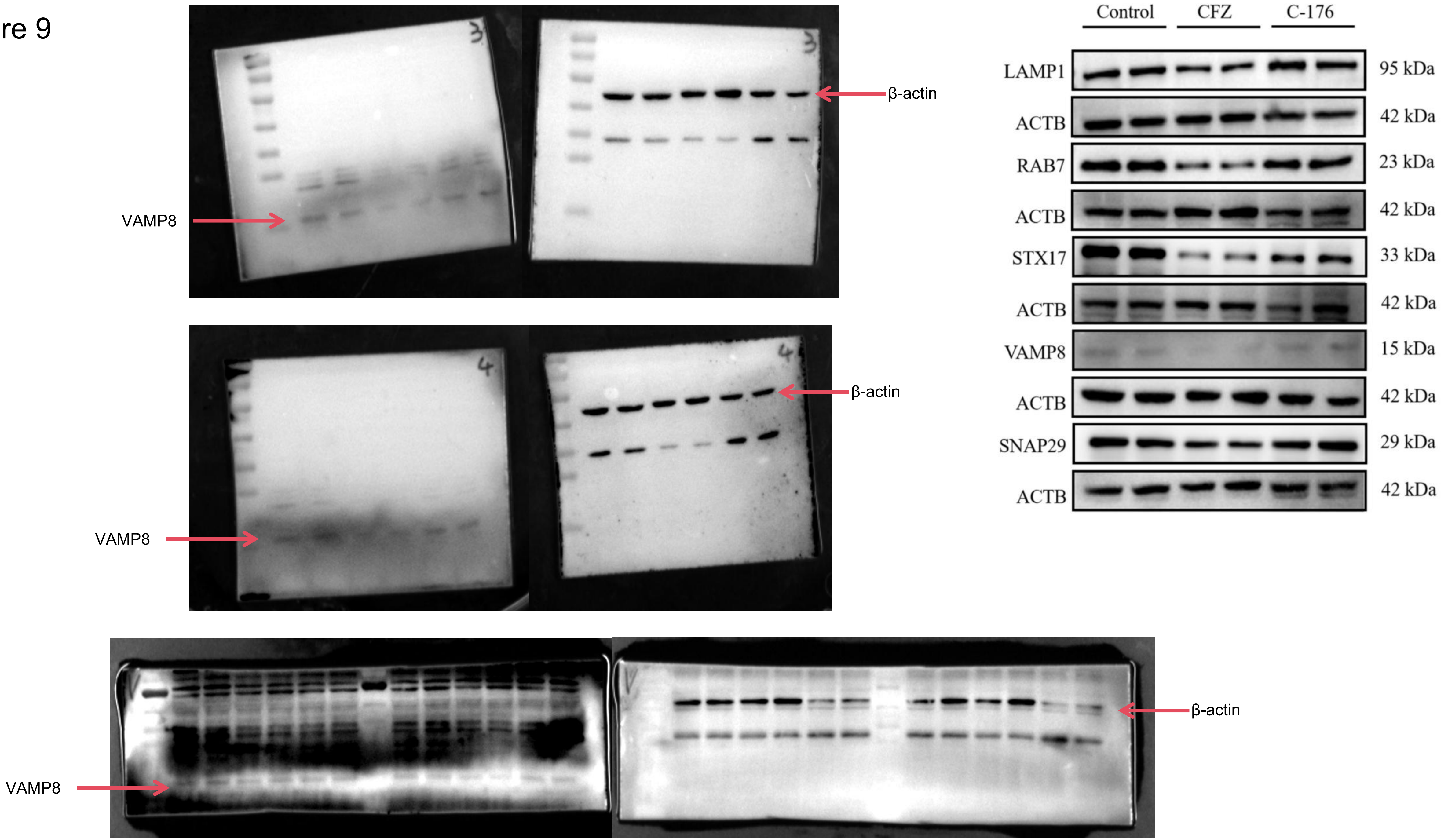
β-actin
VAMP8
β-actin
VAMP8
β-actin
VAMP8
Control
CFZ
C-176
LAMP1
95 kDa
ACTB
42 kDa
RAB7
23 kDa
ACTB
42 kDa
STX17
33 kDa
ACTB
42 kDa
VAMP8
15 kDa
ACTB
42 kDa
SNAP29
29 kDa
ACTB
42 kDa

Figure 9

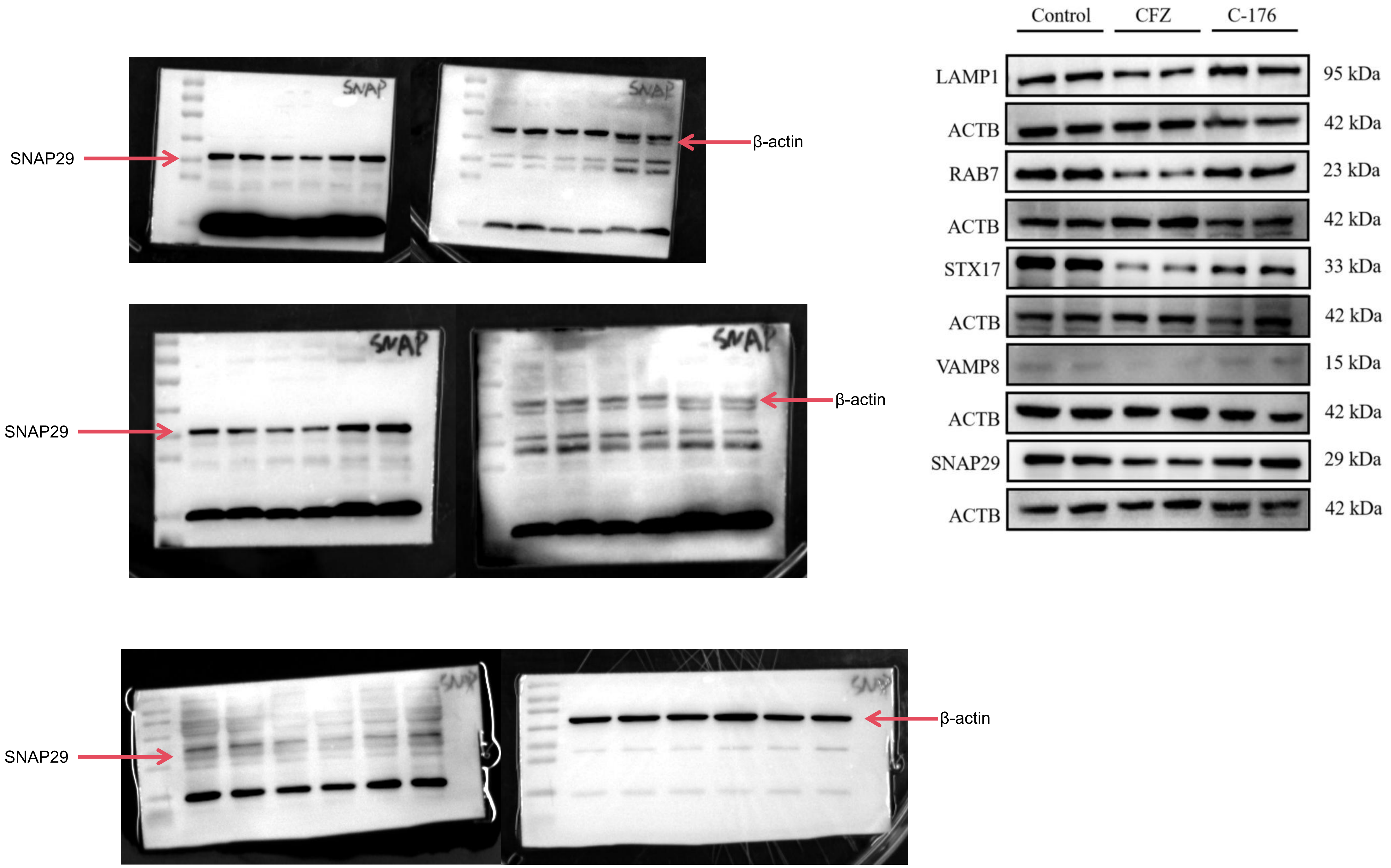
SNAP29
β-actin
SNAP29
β-actin
SNAP29
β-actin
Control
CFZ
C-176
LAMP1
ACTB
RAB7
ACTB
STX17
ACTB
VAMP8
ACTB
SNAP29
ACTB
95 kDa
42 kDa
23 kDa
42 kDa
33 kDa
42 kDa
15 kDa
42 kDa
29 kDa
42 kDa
