# Supplementary material for: Carfilzomib Induces Cardiotoxicity by Blocking Autophagic Flux Through the cGAS-STING Signaling Pathway
Source: Biomolecules. 2026 Jun 11;16(6):854. doi: 10.3390/biom16060854 (PMC13297306; doi:10.3390/biom16060854)
Supplement: Supplementary file 1 [file biomolecules-16-00854-s001.zip › biomolecules-4233931-supplementary.v3.pdf]

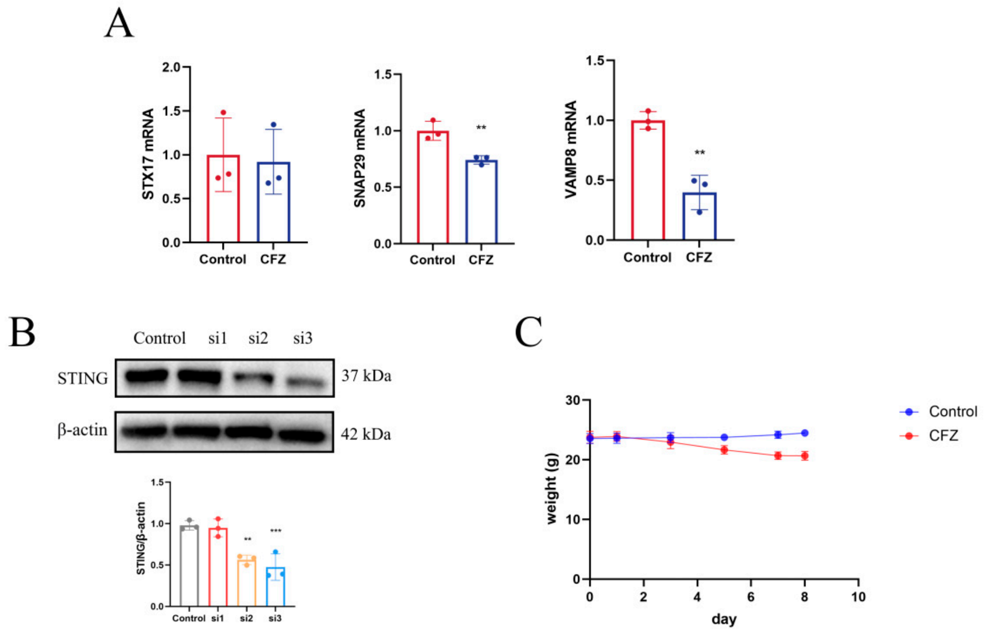

Supplementary Figure S1. (A) Statistical graph of changes in STX17, SNAP29 and VAMP8 mRNA expression. (B) Verification by Western Blot after STING knockdown (n = 3). (C) Weight changes of mice after injection of CFZ (n = 15). Data represent the mean ± SD; $^{**}P < 0.01$, $^{***}P < 0.001$ vs. the control group

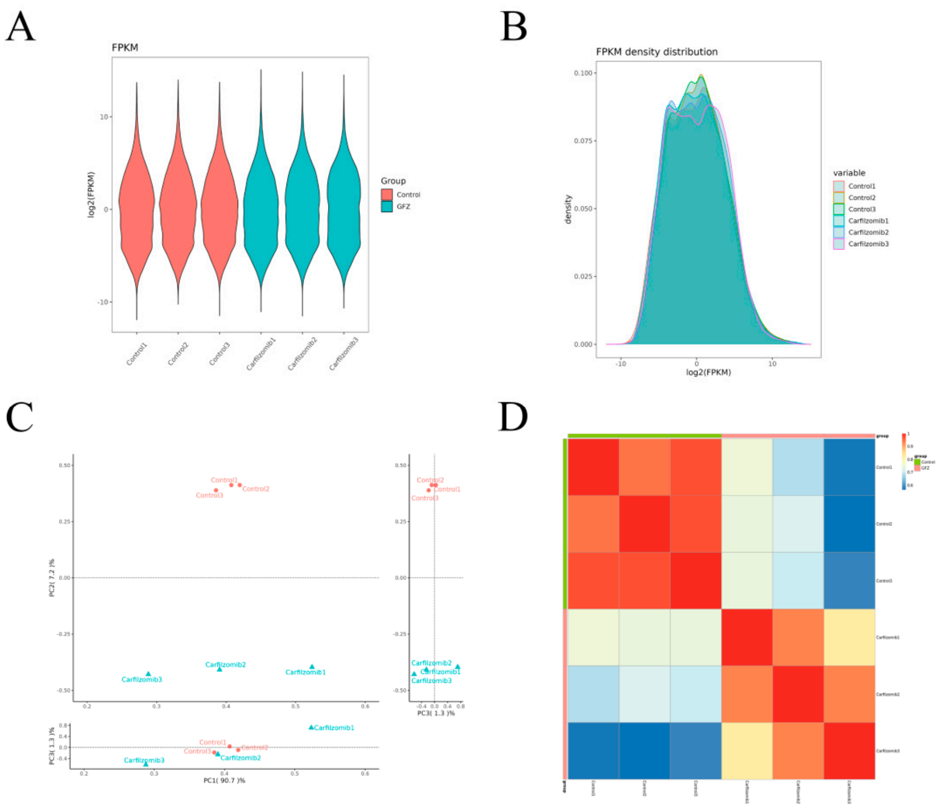

Supplementary Figure S2. (A) ViolinPlot. (B) DensityPlot. (C) PCA. (D) SampleCorrelation.groupHeatmap.
