# Supplementary figures and images for: Carfilzomib Induces Cardiotoxicity by Blocking Autophagic Flux Through the cGAS-STING Signaling Pathway
Source: Biomolecules. 2026 Jun 11;16(6):854. doi: 10.3390/biom16060854 (PMC13297306; doi:10.3390/biom16060854)

# AUTOPHAGY-ANIMAL\_52

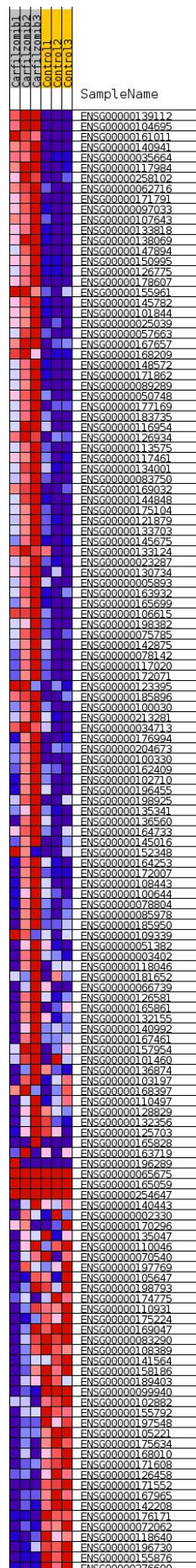

# PROTEIN\_PROCESSING\_IN\_ENDOPLASMIC\_RETICULUM\_4

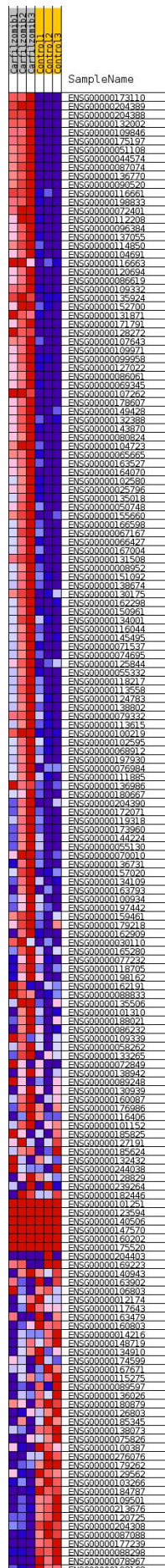

Supplement: Supplementary file 1 [file biomolecules-16-00854-s001.zip › Figure 5E.pdf]
